# The defocalizing effect of international courts: Evidence from maritime delimitation practices

**DOI:** 10.1007/s11558-024-09545-4

**Published:** 2024-06-29

**Authors:** Ezgi Yildiz, Umut Yüksel

**Affiliations:** 1 California State University, Long Beach, Long Beach, CA USA; 2 Universitat Pompeu Fabra, Barcelona, Spain; 3 Geneva Graduate Institute, Geneva, Switzerland

**Keywords:** International courts, Focal points, Regime complexity, Maritime boundaries, International Court of Justice, International law, C10, F35, F55, K33, Q34, Q35

## Abstract

Can international courts influence state policies and facilitate interstate cooperation? Existing literature argues that they can. Courts can make cooperative outcomes easier for states by formulating or endorsing rules around which state expectations and practice can converge. While it is widely assumed that court rulings may become focal points and play a role in harmonizing state practices, we know little about the conditions under which they have such an effect. We suggest that court rulings can often have an opposite, *defocalizing effect*, which may durably harm the prospects of convergence around what the law requires. We introduce defocalization as a process and discuss its possible types and implications. We argue that defocalization may be driven by incongruence of court rulings with existing treaty law and state practice and inconsistency of rulings over time. We illustrate our argument by examining the effect of key judicial rulings on the convergence of state views about the appropriate maritime delimitation rules by relying on an original dataset. Our findings show how *defocalization* unfolds and suggest that complexity can accumulate over time through legal rulings that are incongruent with existing state practice or treaty law, and can be maintained through inconsistent court decisions.

**Supplementary Information:**

The online version contains supplementary material available at 10.1007/s11558-024-09545-4.

## Introduction

International relations and international law scholarship often posit that judicial bodies facilitate interstate cooperation by clarifying international law. In particular, international courts may fill in incomplete contracts (Carrubba & Gabel, 2017; Hadfield, 2015), and more generally, articulate the state of the law (Stone Sweet, 2004; Alter & Helfer, 2010; Helfer & Alter, 2013). Relatedly, legalization scholars observe that states often delegate to judicial bodies, which are called upon to clarify the law by making ambiguous obligations precise (Abbott & Snidal, 2000; Linsenmaier et al., 2021, 520). Other accounts have shown that not only do these courts resolve the disputes submitted to them, but they also influence states that are not directly involved in those disputes (Helfer & Voeten, 2014; Kucik & Pelc, 2016). Disputing states as well as third states may follow court rulings to preempt future judicial review or sanctions (Johns, 2012, 2015; Pavone & Stiansen, 2022). They may also follow them due to courts’ “persuasive authority” and ability to influence policy discussions at the national level (Helfer & Voeten, 2014, 81–82). Existing literature thus expects that courts influence state behavior at large, which may help foster policy harmonization and cooperation between states.

The link between judicial decisions and subsequent policy harmonization and interstate cooperation often hinges on the effect of judicial decisions on third states, that is, states other than those that are directly part of proceedings before a court. The expressive theory of international law has long suggested that legal rules and judgments could help actors coordinate their expectations and behavior even in the absence of any threat of sanction or any possibility of enforcement (McAdams, 2005; see also Huth et al., 2013). The law made and clarified by judicial processes helps third states come to similar views by making certain solutions stand out as *focal*^1^ and “sharpening common understandings as to what formal and informal rules require” (McAdams, 2005, 1080). Court decisions can thus reduce uncertainty and prevent policy discord by serving as focal points around which states can coordinate their policies. We call this the *focalizing effect* of courts.

This effect and the resulting focalization process have several benefits. Some of these benefits have been widely discussed in the context of recent debates on judicialization and courts’ shadow effect (Alter et al., 2019; Alter, 2021). For example, assuming that court rulings reduce uncertainty, Sara Mitchell and Andrew Owsiak contend that the prospects of adjudication motivate states to conclude out-of-court agreements (Mitchell & Owsiak, 2021). Yet the existing literature has not paid sufficient attention to the possibility that judicial lawmaking can “ambiguat[e] clear conventions” as much as it can clarify unclear ones (McAdams, 2005, 1115). When this is the case, court rulings do not only fail to serve as focal points, but instead contribute to making the law more uncertain for all states. This is what we call the *defocalizing effect* of courts.

We argue that courts can make the law more uncertain when they deliver *decisions of general application* that are *incongruent* with the treaty law and the state practice existing at the time (Franck, 2005). This uncertainty is perpetuated further if the rulings are *inconsistent* across time. Decisions that contradict existing rules and practices or earlier court decisions can enduringly harm the chances of policy convergence in the long term. Divergent state practices can proliferate as incongruent or inconsistent court judgments increase the number of alternative rules. These alternatives, in turn, undercut the chances of a single rule to be accepted and followed as the focal rule by states to preempt or resolve disputes. In short, this is how the courts’ *defocalizing effect* operates.^2^

We provide one of the first empirical examinations of the defocalizing effect of international court rulings in an area with high distributional stakes: *maritime delimitation*. Maritime delimitation refers to the process by which neighboring states delineate their respective zones of maritime jurisdiction. We especially focus on the delimitation of the *continental shelf*, a maritime zone that emerged in the post-WWII period.^3^

Neighboring states may delimit their continental shelf in two main ways: by concluding bilateral treaties or delegating delimitation to judicial bodies. Legal scholars agree that courts and tribunals have played a critical role in formulating and applying a host of different rules and methods to the continental shelf delimitation exercise (Lando, 2019; Tanaka, 2019). Their role has been especially important since the 1982 United Nations Law of the Sea Convention (UNCLOS) does not provide any clear guidance as to the precise method states should use to delimit their continental shelf boundaries (Rothwell, 2021). A major point of contention in earlier judicial decisions was the status of the *equidistance* principle, which corresponds to drawing a boundary equally distant from both states’ coasts. We exploit the fact that the most prominent international court, the International Court of Justice (ICJ), has taken different views on the status of equidistance as a default rule over the course of its maritime delimitation jurisprudence.

We expect that the ICJ’s incongruent and inconsistent views on the status of equidistance played an important role in undermining the rule’s popularity and contributed to diversifying state practice. We test these expectations using a comprehensive dataset of state positions on their preferred continental shelf delimitation methods. By limiting the scope of our analysis, we are able to trace how state understandings of the rules of maritime delimitation evolve in parallel to judicial rulings. We find some support for our expectations. Subsequent to key court rulings, equidistance is undermined to a sufficient extent such that diversity increases in state practice, consistent with what we expect to see in a *defocalization* process. The alternative factors we consider are unlikely to be the main drivers of the process, which remains difficult to explain without taking judicial input into consideration.

Our contribution fills three important gaps in the literature. First, the scholarship has worked on the assumption that judicial decisions, especially those made by permanent courts, lead to more certainty.^4^ We highlight court rulings’ *defocalizing effect* as a neglected yet plausible consequence of judicial lawmaking. Second, while the ways in which international law and institutions can provide focal points have been widely discussed, we know little about the process through which plausible focal points can fail to emerge. We suggest that *mixed messaging*—incongruence and inconsistency in the responses given to the same questions—is an important factor that hinders focalization. Third, we distinguish a particular type of complexity in world politics—one that has its origins in a single institution over time, rather than the interactions between multiple overlapping institutions (Alter & Meunier, 2009; Morin & Orsini, 2013; Raustiala & Victor, 2004). By doing so, we identify mixed messaging by a focal institution as a plausible mechanism by which complexity can be created and sustained.

## Focal rules in complex regimes

We define a *focal rule* as a rule, principle, or method that stands out to actors that are interested in policy coordination and cooperation. *Focal rules* increase the prospect for cooperation by narrowing down the range of outcomes states bargain over.^5^ In international law, many possible *focal rules* come to mind. For example, the interpretation of treaties is usually guided by the Article 31(1) of the Vienna Convention on the Law of Treaties, which states that “A treaty shall be interpreted in good faith in accordance with the ordinary meaning to be given to the terms of the treaty in their context and in the light of its object and purpose.” Although the rule leaves many parameters to be defined (for instance, “ordinary meaning” or “object and purpose”), it provides a methodical approach to interpretation.

Focal rules are especially useful in hitherto under-legalized domains that are just beginning to experience increasing interstate activity where there may be a pressing need to avoid discordant policies to achieve cooperative outcomes with more ease. Conversely, the multiplicity of alternative or rival rules are viewed as undesirable for cooperative outcomes. For example, the early regime complexity literature views multiple alternatives as a source of divergence and conflict, and hence detrimental to institutional efficiency and interstate cooperation (Alter & Meunier, 2009; Drezner, 2009; Hofmann, 2009, 2011).^6^ Regime complexity can create room for rivalry, contestation, and inefficiency, as states use institutional overlaps and inconsistencies to their advantage (Wisken & Kreuder-Sonnen, 2020, 124). What contributes to this adverse effect is the rule ambiguity promoted by overlapping regimes governing the same issue area (Alter & Meunier, 2009, 16). This is particularly problematic because, in the absence of a clear hierarchy or a central authority, this ambiguity translates into incoherence of laws and divergence of state practice (Alter & Meunier, 2009, 16–18). Kal Raustiala and David Victor argue that regime complexity is a consequence of increased legalization in world politics, where interactions between different regimes will ultimately lead to “legal inconsistencies” (Raustiala & Victor, 2004, 280, 300). Daniel Drezner similarly argues that “institutional proliferation can dilute the power of previously constructed focal points. Regime complexity inevitable (*sic*) increases the number of possible focal points around which rules and expectations can converge” (Drezner, 2009, 65).

This last observation is the epitome of the regime complexity literature’s general tendency of taking institutional multiplicity as a proxy for mixed messaging (Hillebrecht, 2019, 276). While insightful, these approaches often overlook the temporal variation of complexity and its possible production through the work of a single institution.^7^ Indeed, when multiple rules are endorsed, their collective effect hampers the potential for creating focal points. However, in some cases, the real culprit of divergence appears to be incoherent messages that may often accumulate over time rather than the multiplicity of institutions. One of our contributions is to show how even a single, authoritative institution may be the source of mixed messaging that can prevent the emergence of a focal rule.

The role of central authorities in establishing focal rules has been underlined in previous research. For example, Mette Eilstrup-Sangiovanni challenges the assumption that regime complexity always leads to conflictual outcomes and shows how previously fragmented arrangements can be ordered around focal institutions (Eilstrup-Sangiovanni, 2022). The significance of focal authorities comes out the clearest in the context of international courts and tribunals, whose proliferation is often considered to be a symptom of fragmentation of international legal order (Fischer-Lescano & Teubner, 2003). For example, Georges Abi-Saab underlines the need for a common framework, a pivot for international courts and tribunals to coordinate around. He argues that “in the present-day international legal order, only the ICJ can play this role,” and proposes that the ICJ is best positioned to serve as a reference point for the international legal order (Abi-Saab, 1998, 929). Our analysis contributes to the evolving literature on regime complexity and helps us uncover what happens when central authorities that are placed to focalize expectations do not do so.

## Towards a theory of defocalization

**Fig. 1:**
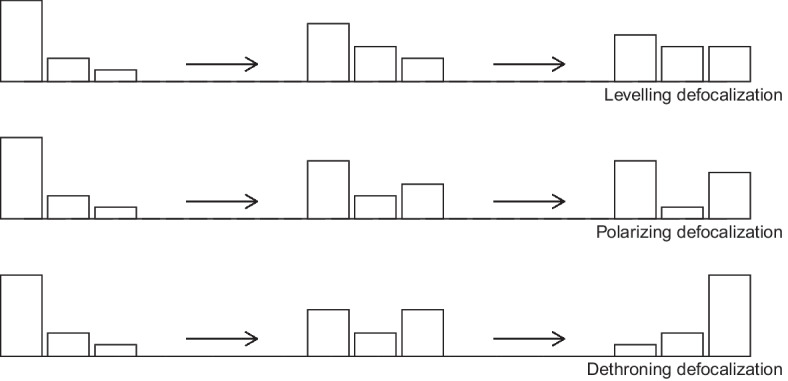
Three types of defocalization, from the perspective of the initial focal rule, the bar on the left in each panel

We suggest conceptualizing *defocalization* as the gradual loss of popularity of an existing or a plausible focal rule. What each defocalization process has in common is the initial fall in the popularity of the prior focal rule, often accompanied by the increase in the popularity of another or several other rules. After this initial stage, the process may take different shapes. To guide our thinking, we propose a typology based on the observable outcomes along the process of defocalization. For simplicity, we do this from the perspective of the prior, plausible focal rule. Figure 1 presents this typology.

*Levelling defocalization* can be conceived as a process that renders each alternative rule almost as appealing and popular as the prior focal rule. Once it runs its course, it culminates in a situation where the prior focal rule is equally viable as any of its competing alternatives. *Polarizing defocalization* refers to a process by the end of which an alternative rule rivals the prior focal rule in popularity. Two rival rules dominate the practice, with most states clustering around them. This type of defocalization can involve rival camps, made up of similar and stable numbers of states. *Dethroning defocalization* occurs when a dominant, plausibly focal rule gets overtaken by another. If an alternative rule replaces the formerly dominant rule and continues to grow in popularity, a (re)focalization process may begin around this alternative. From the perspective of the prior focal rule, however, what happens is still a form of defocalization as its once-dominant status is supplanted by another rule.^8^

While these defocalization processes can take place without the involvement of any court, they can also be triggered or influenced by courts. First, courts can provide various alternatives without favoring any single one consistently, which may lead to each alternative being seen as equally viable or acceptable. Second, courts can spur polarization by endorsing the preferred rule of one side or another, usually in an ideologically or politically contested field, which may further fuel contestation. Third, they can favor an alternative rule against a dominant rule, which may help that rule rival or overtake the dominant one. Our focus here is the initial stages of defocalization which may culminate in one or the other end point by which our typology is inspired. What is important in the initial stages of defocalization is that a plausibly focal rule is undermined, and its alternatives begin to appear more viable.

Courts can influence the viability of different solutions relative to a plausible focal rule in two main ways. First, judicial decisions may be *incongruent* with the existing law or practice. By *incongruence*, we mean a lack of fit between a court decision and existing treaty law and state practice, where practice can comprise views or legal positions expressed by state officials or acted upon by states. International courts are known to develop law and go beyond the scope of the treaties they interpret (French, 2006; Venzke, 2011), which may sometimes put them at odds with existing treaty provisions or state practice (Alter, 1998; Danner, 2006). In such instances, the new rules or understandings that the courts offer are likely to be drivers of divergence instead of convergence. This is because court rulings cannot automatically override the existing treaty law; they rather serve as sources of (or justifications for) alternative rules.^9^

Second, judicial decisions may also be *inconsistent* with each other. By *inconsistency*, we mean the lack of consistency in the messages sent by the court over time, especially when they deal with rules of general application rather than idiosyncratic solutions to specific disputes (Blum, 2009; Franck, 2005). This can come in many shapes. Inconsistency can be incremental, resulting from evolving interpretations over time. It can also be more sudden and unpredictable when courts deviate from jurisprudence in unexpected ways without a clear direction. Importantly, later decisions do not necessarily undermine the authority of earlier rulings, and the result may be an increase in the number of distinct, alternative solutions. Beyond its normative implications, such inconsistency creates practical consequences for policy coordination by offering states a set of alternatives to pick and choose from when legally arguing their claims.^10^ The important point here is that alternatives can accumulate over time as later court decisions do not “explicitly reverse jurisprudence” (Guillaume, 2011, 12).

Before we move on, some caveats are in order. First, we do not suggest that the opposite of inconsistency—consistency—necessarily has a focalizing effect. Sometimes, consistency may perpetuate existing divisions and ambiguities. Consider the rule of self-defense against non-state actors. In its 1986 *Nicaragua* judgment, the ICJ laid out a high threshold for invoking self-defense against non-state actors.^11^ Essentially, to argue for self-defense against a non-state actor, a state had to show that the actions of that non-state entity could clearly be attributed to a state. While *Nicaragua* was in line with the existing understandings at the time, several states came to view that self-defense could be invoked against non-state actors especially in the aftermath of 9/11 (Green, 2009, 47). A coalition of states, including the UK, France, Canada, Germany, Australia, the Netherlands, Poland, Belgium, and New Zealand, supported the US’s declaration that it would exercise its right to self-defense against AI-Qaeda and the Afghan Taliban (UNSC, 2001). Others, like Israel, Russia, Uganda, Kenya, and Turkey also expressed their support for this new understanding in other regional contexts, while a few countries such as Brazil and Mexico expressed opposition 12 (Martinez Esponda, 2023). Yet, the ICJ did not address these developments and issued two decisions that largely repeated the reasoning in *Nicaragua* (*Wall Advisory Opinion,*^13^ and *DRC v. Congo*^14^). 

Second, we do not suggest that consistency or congruence has some intrinsic normative value. Sometimes, it may be worth taking inconsistent decisions to reflect changing understandings or render incongruent decisions to advance rights that are not widely upheld. The European Court of Human Right’s (ECtHR)’s support for the abolishment capital punishment after issuing many decisions that stayed shy of doing so could exemplify this dynamic. While state practice progressively eliminated capital punishment, the ECtHR initially delivered cautious judgments, waiting for change through a traditional treaty amendment procedure. For example, in *Öcalan v. Turkey* (2005), the ECtHR referred to the states’ intention to abolish death penalty through the 1983 Protocol 6 (abolishing death penalty except in times of peace) and the 2002 Protocol 13 (abolishing capital punishment in all circumstances in 2002). However, it refused to issue a ruling that could bolster these trends.^15^ The ECtHR changed its position in *Al-Saadoon and Mufdhi v. the United Kingdom* (2010), where it established that nearly all member states either banned or introduced a moratorium on capital punishment.^16^ Hence this inconsistent ruling built on and provided further support to the existing state practice that was increasingly unified in its ambition to abolish death penalty.

Third, the effects of (in)congruence and (in)consistency may not always work in the same direction. An inconsistent decision that is congruent with state practice may actually foster focalization. The foregoing example on the ECtHR’s role in the abolishment of death penalty in Europe is a case in point. The ECtHR closely observed changing state practice and rendered a decision that endorsed and ultimately consolidated state practice, even though it meant issuing a ruling that is inconsistent with its established case law (Cheeseman, 2017; Yildiz, 2020). Conversely, courts’ unwillingness to adjust their approach (that is, their consistency with their own precedent) despite converging trends around a new rule in state practice may sometimes prevent focalization*.* A consistent ruling that is incongruent with emerging state practice can hamper focalization. For example, the ICJ dealt a blow to the consolidation of a new understanding of self-defense by being reluctant to endorse states’ claim for self-defense against non-state actors, despite emerging trends in that direction in the early 2000s (Green, 2009).

Finally, we do not suggest that court rulings are the only influence on state practice. State practice is naturally shaped by economic and political interests or ideology. Still, how courts deal with and respond to trends is not inconsequential. A case in point is the attempt by the Appellate Body (AB) of the World Trade Organization (WTO) to settle on a definition of state-owned enterprises (Ahn, 2021; Gao, 2021). While there is no clear definition of state-owned enterprises in the existing WTO law, there emerged two main approaches: the “meaningful control” test (narrow definition), advocated primarily by the US and the EU, and the “government function” test (broad definition), advocated by China and India to a lesser extent (Shaffer & Gao, 2018). The AB first attempted to accommodate China’s approach in the 2010 *US–Anti-Dumping and Countervailing Duties (China)* case (Ding, 2014). Then, it opted for determining the public body notion on a case-by-case basis, an approach that was described as “confusing and convoluted due to the incongruity between panels and the AB [Appellate Body]” (Ahn, 2021, 67). Later cases, especially after 2018, were somewhat deferential to the “meaningful control” test, yet the US kept insisting on its interpretation and chose to appeal even a favorable ruling just because its starting point was the “government function” approach (Ahn, 2021, 64). Hence, the AB’s inconsistency in dealing with political fault lines demarcated by the US and China rivalry did not help promote rule consolidation in this area.

State practice can also be divided along moral fault lines. We see this in the case of LGBT rights in Europe, where the concept of family has expanded to ensure the equal treatment of same-sex couples. The ECtHR played a crucial role in creating and expanding the range of rights that the LGBT community could enjoy, and focalized state practice by using majoritarian activism (Helfer & Voeten, 2014). For example, in 2010, the ECtHR issued *Schalk and Kopf v. Austria*, where the Court found that same-sex relationships could be considered as family units. Yet, it added that member states were under no obligation to protect that “family life” by allowing same-sex couples to marry.^17^ Around that time, twenty-two of forty-seven members offered legal recognition to same-sex couples (Fredman, 2019, 341–42).^18^ Trends then began favoring this broader family notion,^19^ although there is still not a full consolidation marriage rights in Eastern European or less-liberal leaning states such as Armenia, Georgia, Azerbaijan, Turkey, and Russia (before its exit) (Helfer & Ryan, 2022). Full focalization seems to be stalled along moral fault lines and ECtHR’s own case law might have provided some ammunition to the critics of expanding the  definition of family units.^20^

### Scope conditions

Our theory of defocalization is relevant to cases where more states are expected to take positions on a question of international law, and some *prior practice* exists regarding the same question such that there is a potential for converging around a common understanding. As we discuss below, we will test our expectations in such a case—maritime delimitation policies. In this case, there was a growing understanding reflected in (limited) state practice that a particular rule (*equidistance*) had a default, focal rule status. It is in such cases that we can best talk about *defocalization.* Our theory is also limited in application to cases where the international courts’ contributions can stand out as signals, for instance, because treaty rules do not provide clear guidance or we are dealing with a new field (or new issues in an existing field). Many areas of international law are like this; rules are imprecise and delegated authorities are there to make them more precise (Abbott et al., 2000; Koskenniemi, 2009).

Finally, our theory is most relevant for cases where different rules imply different *distributional outcomes* for states. This is the case in many fields such as trade, disarmament, environment, and the use of outer space. While *incongruence* and *inconsistency* create the possibility of policy discord, it is the *distributional consequences* that drive and sustain actual divergence among policies. It might be relatively easier to coordinate state policies around rules with low distributional consequences, especially when such rules benefit all states to a certain extent, by providing predictability for instance (Helfer & Wuerth, 2016, 568). On the contrary, the existence of high distributional consequences associated with alternative rules reduces the likelihood that states adopt similar policies when such alternatives are available.

### Expectations

What is common to all types of defocalization is that the plausible focal rule loses traction. All things equal, we expect rules to lose their popularity after a court favors a competing rule, rendering it a legally viable option for third states to adopt. The loss of popularity should be greater if the court decision is highly incongruent with existing practice and treaties. This is because such a decision creates a particularly hard shock at a time when most states could legitimately expect focalization around previously popular rule. Such incongruence can hamper the perceived inevitability of a potential focal rule. We propose the following hypothesis:

**Hypothesis 1** The more incongruent a court ruling is with a plausible focal rule that finds support in existing practice and treaty law, the more that rule will lose its popularity.

Another observable implication of defocalization is the increasing diversity in state practice. We expect that incongruence also leads to an increase in diversity of state positions, with diversity defined as the existence of a set of rules that are supported in similar proportions—like a *leveled* playing field where each rule has comparable attractiveness. The diversity increases as the focal rule loses its popularity, at least in the short run. The change in diversity is driven by sets of choices: First, some states will change their positions and adopt the promoted rule, while some will stick to their prior positions, presumably as they are not made better off by the new rule. Second, states without prior preferences will select either the promoted rule or one of the alternatives. Based on these considerations, we propose the following hypothesis:

**Hypothesis 2** In periods following incongruent rulings diversity is likely to increase.

Incongruence may create the conditions for focalization around newly promoted alternative rule in the long run—similar to how dethroning focalization process would look. But the prospects of this development can be undermined especially since courts are called on many times and their signals can change over time. In this case, trends in popularity for plausible focal rules and their alternatives may be punctuated by *inconsistent* court decisions, especially if the inconsistency goes beyond incremental change in interpretations. The periodic changes in the promoted rule can lead to fluctuations in the popularity of available rules, making it difficult for any alternative to become focal. With regards to inconsistency’s effect on the plausible focal rule, we suggest the following:

**Hypothesis 3** Inconsistent rulings that promote a rule other than the plausible focal rule diminish the popularity of the latter.

**Fig. 2:**
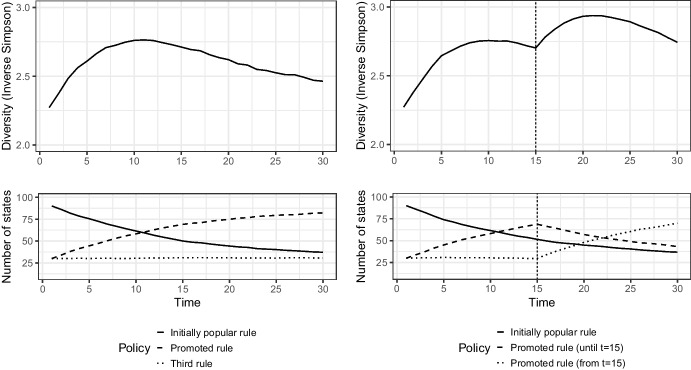
How a stylized defocalization process may look with one court signal in the panels on the left (at time 0) and two signals on the right (at times 0 and 15). The creation of these figures is explained in Appendix 4

This process also has implications for diversity. As the promoted rule changes over time, we expect diversity to experience intermittent surges after each inconsistent ruling. This is because each promotion may make that alternative relatively more popular in the subsequent period. This may lead to some states abandoning their positions to adopt the promoted alternative, at least in the short run, until the court changes its stance again. Similar to the stylized depiction in Fig. 2, inconsistency can lead to a jump in diversity. We can thus propose that inconsistency increases diversity and may keep it high due to these periodic shifts.

**Hypothesis 4** Diversity is likely to increase after periods of inconsistent rulings.

We consider that the inconsistency can come in different shapes. Interpretations can incrementally evolve over time, or the court may make sudden and particularly unexpected decisions, which casts doubts about the future direction of the jurisprudence. As corollaries to H3 and H4, we suggest that this type of inconsistency has a greater potential to undermine the plausible focal rule and increase diversity.

The illustration in Fig. 2 helps visualize the correspondence between policy distribution and diversity as they may evolve according to court signals. States can choose among three policies, and depending on their choice, a diversity score is calculated that captures the number of policies and the distribution of states across policies.^21^ In the left panel, the court provides one signal; in the right panel, it provides two distinct ones, endorsing two different policies. These stylized figures correspond to our expectations that court signals that go against an already popular rule are likely to increase diversity in the short run, although diversity can begin to decrease again if the newly promoted rule becomes dominant. When the court promotes two distinct rules at two different points in time, any possible decrease in diversity can be cut short and diversity can instead increase. 

## Case selection: Judicial decisions on maritime delimitation

Four features make the field of maritime delimitation ideal to examine the role of courts—particularly that of the ICJ—on the processes of policy harmonization. First, this is a highly legalized field, where states formulate and contest claims using legal terms. Second, the provisions on drawing common maritime boundaries, including provisions codified in treaties, are often highly *imprecise*. That is why international courts have been called upon to clarify them. Indeed, adjudication has played an important role in the development of the rules on continental shelf delimitation (Rangel, 2006, 358), with the ICJ prominently figuring as the authoritative dispute settler in this field. Third, maritime delimitation is an isolated area that is unlikely to be affected by broader political developments. It is, therefore, more plausible that any significant change in practices can be attributed to a judicial intervention on the question of delimitation itself instead of other exogenous shocks such as political or economic crises, or natural disasters. Fourth, maritime boundary-making involves rules and interpretations with potential distributional consequences. These arise from the fact that maritime delimitation has a zero-sum component: states have *exclusive* rights over the maritime zones allocated to them. The possibility for states to obtain more by following an alternative rule makes the provision of alternatives more impactful in comparison to other areas where such distributional consequences are non-existent or negligeable.

We assess all the relevant rulings issued by the ICJ and give an account of the judicial signals originating from each of them. Across practice and judicial rulings, three main methods of delimitation came to be recognized in the established literature (Lando, 2019). One of the earliest established rules was the *equidistance* principle, which essentially entailed drawing a line that is equally distant from the relevant coasts of each neighboring state. This method was codified under Article 6 of the 1958 Continental Shelf Convention (CSC) as the default rule, because it was the rule that would impose itself in practice unless states agreed otherwise. What made equidistance attractive was the fact that it was a simple method with a straightforward application.^22^ Following equidistance, technical experts could draw most of the boundaries in the word without needing political negotiations or courts’ involvement.

This method was rivalled by two other approaches that crystalized over time. One of them was *equitable principles*, which allowed parties to take many factors into account in reaching a division that is equitable. Yet, this vague and highly subjective approach left many parameters to be negotiated, rendering the delimitation practice rather complicated. This could accommodate a combination of methods that do not require the drawing of an equidistance line, which is why we call this group of methods *nonequidistance.* A third approach, *modified equidistance*, combined elements from these two methods. This method requires delimitation exercise to start from a provisional equidistance line, and to make adjustments considering geographical factors (e.g., existence of islands, lengths of coasts). Modified equidistance is compatible with the 1958 CSC, which prescribes that equidistance line can be adjusted in light of special circumstances when needed, while emphasizing that, in the absence of an agreement, the default rule is equidistance.

Table 1 lists all the relevant rulings concerning continental shelf delimitation and the solutions offered by the ICJ, arbitral tribunals, and International Tribunal for the Law of the Sea (ITLOS).^23^

**Table 1:**
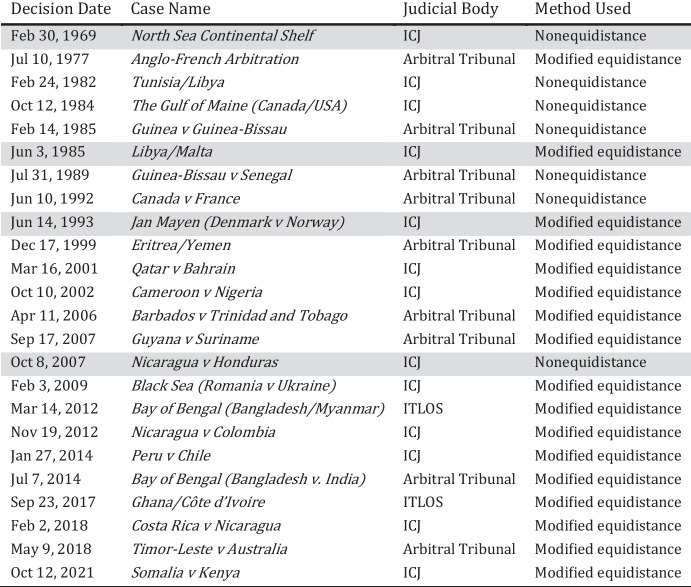
Judgments and awards on continental shelf delimitation

The four decisions that we pay special attention to are highlighted

As Table 1 shows, courts’, especially the ICJ’s, approach changed over time. In its earlier decisions, until 1985, the ICJ favored nonequidistance, and then it pivoted towards favoring modified equidistance. Yet, there were a few unexpected turns along the way, including one as recently as 2007 in *Nicaragua v Honduras*,^24^ which experts describe as “a major departure from the previous case law” (Tanaka, 2008, 338), and a sign that the Court “might allow more flexibility in the choice of method in future delimitations” (Lathrop, 2008, 118).

We identify four key rulings that provide new information about the continental shelf delimitation rule. As expected, such new information may be released when the court issues unexpected rulings that are incongruent with existing law and state practice and when they are inconsistent with earlier decisions. These four key rulings are: *North Sea Continental Shelf*, *Libya/Malta, Jan Mayen,* and *Nicaragua v Honduras*. They represent different levels of incongruence and inconsistency and are thus particularly good candidates for assessing the effects of these factors on any possible defocalization process.^25^

Two caveats are in order regarding these cut-off points. First, we have decided not to consider the decisions of other courts and tribunals as viable cut-off points. Some possible cut-off points could have included the 1977 Anglo-French Tribunal ruling, which precedes ICJ’s 1985 and 1993 rulings in promoting modified equidistance, and ITLOS rulings. The 1977 ruling was pronounced by an *ad hoc* tribunal that was called upon to delimit a specific boundary and not to offer generalizable understandings on the state of the law (Blecher, 1979, 65). Moreover, the reasoning in the Anglo-French Tribunal decision is overall not inconsistent with the ICJ’s *North Sea* decision. The Tribunal attempted to find a compromise between the 1958 Convention and the *North Sea* ruling by adhering to equitable principles (Blecher, 1979, 69). Second, we have also decided not to consider ITLOS rulings as cut-off points, because this body has closely followed the ICJ’s case law, particularly in the field of maritime delimitation (Wolfrum, 2013). None of their decisions present a break from the most recent prior ICJ rulings. In other issue areas, future studies may consider if multiplicity of actors are relevant. This would typically be the case if multiple courts either do or at least can be expected to follow different methodologies, reach different outcomes, and send different signals. This was not the case here.

In discussing these cases and the periods that they demark, we assess the level of congruence as well as inconsistency types.

### North sea continental shelf cases (1969): High incongruence

In this case, Denmark and the Netherlands argued for equidistance whereas Germany opposed it. Hearing both sides, the ICJ essentially sided with Germany and emphatically refused equidistance’s customary status, instead holding that delimitation had to follow *equitable principles*. This ruling thus endorsed an alternative rule, setting equidistance aside in an unexpected way. As Leonard Legault and Blair Hankey (1993, 205) note, “until the 1969 judgment in the *North Sea* cases, most policymakers assumed the existence of a binding legal presumption in favor of the equidistance method, whether under treaty law or customary law.” Our analysis of the state practice at the time supports this claim. In 1969, about 75% of states that had expressed a preference supported equidistance. The ruling is thus ideal to assess the impact of incongruence, as it was a clear case of a highly incongruent ruling that went against treaty law (Article 6, the CSC) and the existing state practice at the time that favored equidistance.

We also expect that this ruling had far-reaching consequences. For instance, it became a reference point for many states during UNCLOS negotiations.^26^ Encouraged by this ruling, many states argued that the equidistance principle had no preferential status, and that the delimitation exercise should be carried out following equitable principles. These states were opposed by others that sought to include equidistance as the default method.^27^ These disagreements continued throughout the negotiations resulting in a minimal agreement around a vague formula under Article 74(1) and 83(1) of the UNCLOS.^28^ This formulation carefully avoided controversial terms such as *equidistance* or *equitable principles,* instead speaking of an *equitable solution*. While this result-oriented compromise appeased the opposing camps, it provided little concrete guidance for continental shelf delimitation. States could practically use any method to reach an equitable solution.

### Libya/Malta (1985): Moderate incongruence and incremental inconsistency

In *Libya/Malta*, the ICJ explained that state practice “falls short of proving the existence of a rule prescribing the use of equidistance, or indeed of any method, as obligatory.”^29^ Yet, it still chose an equidistance-based method that it deemed appropriate for that specific case. It started with an equidistance (median) line between Libyan and Maltese coasts and adjusted it at the expense of Malta justifying it on the basis of their significantly different coastal lengths.

We consider that the inconsistency between the 1969 ruling and *Libya/Malta* is rather incremental though it gives states new information about the appropriateness of equidistance as a starting point in some contexts. This is because the decision reiterated the rule established in the 1969 decision in principle, while in practice it arrived at a solution that used equidistance as a starting point. We deem incongruence to be moderate in this period, as the continued opposition between existing treaty law (the 1958 CSC—the UNCLOS was adopted but not in force) and the ICJ’s solution persisted. Moreover, there was still a sizeable proportion of states supporting equidistance at the time of the decision—about 63%.

### Jan Mayen (1993): Low incongruence and incremental inconsistency

In the 1993 *Jan Mayen* judgment, the ICJ declared that “[p]rima facie, a median line delimitation between opposite coasts results in general in an equitable solution.”^30^ By clearly linking the use of a provisional *equidistance* line to the achievement of an *equitable result*, the ICJ appeared to broaden the application of equidistance as a starting point. This strengthened the approach which started in *Libya/Malta*, and further promoted modified equidistance as an appropriate method. This also was the first of a series of decisions that reaffirmed the use of equidistance as the first step. *Qatar v Bahrain*^31^ and subsequent rulings reiterated this method of beginning with a provisional equidistance line and “equity-correcting” it (Tanaka, 2019, 205).

*Jan Mayen* built on the previous *Libya/Malta* ruling incrementally, strengthening the role of equidistance on the path towards an equitable solution. This came to be called the two-stage methodology, where the court starts with a provisional equidistance line in the first stage and modifies it in the second stage to reach an equitable solution. We consider this decision to have a relatively lower level of incongruence, because the 1958 CSC (that had equidistance as a default rule) had lost most of its relevance, with the UNCLOS entering into force a year after this ruling.

### Nicaragua v Honduras (2007): Low incongruence and unexpected inconsistency

In its 2007 *Nicaragua v Honduras* decision, the ICJ drew a nonequidistant line without even considering equidistance as a first step. Granted, neither of the parties had asked for the use of equidistance. However, the ICJ’s decision not to begin with equidistance nevertheless was unexpected. The *ad hoc* judge Torres Bernárdez went as far as to argue in his dissenting opinion that this ruling dealt a blow to settled jurisprudential trends, and represented a “return to the idea of *sui generis* solutions for each delimitation, in other words a relapse into pragmatism and subjectivity.”^32^

We consider this case to be an example of unexpected inconsistency. While the subsequent rulings brought provisional equidistance line back as early as in 2009, we still expect this decision to have effects going beyond that year for the break it represented from an otherwise linear trajectory towards always using equidistance as the starting point. In terms of incongruence, we again consider this to be lower compared to the first few periods, mainly because the UNCLOS is vague enough to accommodate the method used by the ICJ.

## Data and analysis

 We rely on original data and a series of statistical tests to examine how *incongruence* and *inconsistency* affect the prospects of a plausible focal rule. We also consider how diversity in state practice evolves across periods marked by incongruent and inconsistent rulings.

### A dataset of state positions on the delimitation rule

To assess our expectations, we examine state positions on the appropriate rule for continental shelf delimitation. To identify these, we rely on a corpus composed of a variety of documents with information on such positions. The bulk of our corpus was built by scraping and digitizing all 1341 documents related to maritime boundary delimitation from the website of the UN Division of Ocean Affairs (DOALOS). This included domestic legislation, unilateral declarations, as well as bilateral agreements. When necessary, we used the International Maritime Boundaries (IMB) volumes to obtain information about and the full text of the bilateral agreements that were omitted in DOALOS.^33^ We have complemented our corpus with any other relevant sources indicative of state preferences.

For classification of state positions, we rely on Legault and Hankey (1993), who distinguish between the following three categories: (1) “Equidistance Strict or Simplified,” (2) “Modified Equidistance,” and (3) “Non-equidistance”. The whole corpus was labelled manually according to these categories by three coders. As the corpus was machine-readable, we could use a baseline algorithm to highlight specific words and phrases which could indicate a particular delimitation method.^34^ For instance, we looked for references to “equidistance” or “median line” for classifying documents as holding *strict or simplified equidistance* as the preferred method. When the keywords in context did not allow easy classification—for example, when words indicative of more than one method co-appeared in the same document or when no keyword was found—we have read through the document to categorize it. Each coding decision was reviewed by at least one other coder. When the text did not help us identify the policy conclusively, we used maps and commentaries provided by the IMB volumes as well as the Sovereign Limits database (https://sovereignlimits.com). We also used Legault and Hankey’s existing coding until 1993 to verify our coding decisions about bilateral treaties from that period.

Two further sources were used to determine policy changes. First, we used memorials submitted by states to international courts and tribunals, insofar as the memorials contained information about state positions on continental shelf delimitation. Second, we used multilateral treaty ratification record when the treaty in question favored a specific method. Only the 1958 CSC met this criterion, as it clearly included equidistance as the default method. We coded states that ratified the said convention as preferring equidistance as the delimitation method from the year of their ratification. For example, Denmark ratified the 1958 CSC on 12 June 1963; hence it is coded to have opted for strict or simplified equidistance from 1963 onwards until it adopted a different policy in 1970. We code a state as favoring a particular policy starting from the year in which we observe an act that expresses such a preference and until the year a different preference is expressed. The final data set runs from 1960 to 2019 and codes state preferences on an annual basis.

### Analysis strategy

#### Dependent variables

We use three main dependent variables, corresponding to different sets of tests. First, we assess the popularity of equidistance, the plausible focal rule when the ICJ began rendering its decisions, using a variable that takes a value of *1* if a state made a new policy in which it expressed a preference for equidistance. We use logistic regression to model the probability that equidistance will be the choice when a state expresses a preference for a policy.

Second, we take new policy type as the dependent variable to compare the popularity of other promoted rules to that of equidistance over time. This is a categorical variable that can take three values: *equidistance*, *modified equidistance*, and *nonequidistance*. We use a multinomial logistic regression to compare the probability of choosing one of the other policies over equidistance.

Third, the dependent variable we use to assess the diversification of state policies is diversity, which captures the degree to which state policies are heterogeneous. We operationalize it with the inverse Simpson index, which is already illustrated in Fig. 2 above and calculated as follows:

$$D = \frac{1}{{\sum\nolimits_{i}^{k} {p_{i}^{2} } }}$$

where

*k*: is the number of groups,
*p*_*i*_: is the proportion of each group *i*

With three mutually exclusive policies, the minimum score that can be achieved is 1, corresponding to the situation where one policy is favored by all states and the other two policies are not favored by any state. The maximum score is 3, which occurs when each policy is favored by exactly one third of the states. The inverse Simpson index is measured annually, and depends on the distribution of favored policies each year within our period of observation, that is, between 1960 and 2019.      

We also calculate what we call the *unit-counterfactual contribution to diversity* for each state that expressed a preference for a policy in a given year. This is simply the hypothetical change in diversity that would have been occasioned by a particular state’s move *if that state had been the only one* making a new policy in that year. This measure simply computes a difference in diversity scores that captures the contribution to diversity of that new policy that would have been generated by a state $S$ making a policy $P$ in that year if $S$ had been the only one making a policy that year. This counterfactual score can be presented as follows:

$$\Delta^{*} D_{p} = D - D^{*}_{ - p}$$

where

$\Delta^\ast D_p$ is the counterfactual score, $D$ is the actual diversity score calculated for a given year, and $D_{-p}^\ast$ is the diversity score calculated when we ignore the new policy made by a state in the same year.

We run a series of OLS models to understand the extent to which changes in diversity are related to our independent variables.

### Independent and control variables

Our main independent variables are *incongruence* and *inconsistency*. We operationalize these by periodizing such that each period is defined by a particular level of incongruence and type of inconsistency. We refer to our discussion above and simply summarize our coding in Table 2. As each period represents a unique combination of incongruence level and inconsistency type, we use periods to stand for these combinations in our tests.

**Table 2:** Classification of periods

Cut-off decision year	Prev. 3-year average Prop(EQ) at year of decision	Prop(EQ) at year of decision	Default rule by treaty	Rule used by the ICJ	Incongruence of the decision	Inconsistency of the decision	Period [P]/ Year range for analysis
			EQ				P1 1960–1969
1969	0.81	0.76	EQ	NONEQ	High		P2 1970–1985
1985	0.61	0.63	EQ/None	MODEQ	Moderate	Incremental	P3 1986–1993
1993	0.56	0.60	None	MODEQ	Low	Incremental	P4 1994–2007
2007	0.59	0.59	None	NONEQ	Low	Unexpected	P5 2008–2019

Key: *EQ* Equidistance; *MODEQ* Modified equidistance; *NONEQ* Nonequidistance; *Prop(EQ)* Proportion of states with equidistance as their last policy among all states with a preference. Default rule by treaty captures  equidistance’s default status in treaty law (1958 CSC), and loss of this status when UNCLOS is signed (1982), and especially, when it enters into force (1994)

We use a series of control variables in our tests, especially in the analyses that focus on the popularity of equidistance, the most plausible focal rule. If we can find that equidistance decreases in popularity through incongruent and inconsistent decisions, we will still want to rule out that this decrease can be explained by processes other than those plausibly driven by the court decisions. For this, we control for a series of political, economic, and boundary-related variables that can help explain varying uses of equidistance over time. First, we control for whether the state’s last policy was equidistance. Then, we control for their legal system, whether they ratified UNCLOS, their GDP per capita, and their political regime. Finally, we also control for whether the state has been subject to an ICJ decision or is found in a region where others went to adjudication over their maritime boundaries. We discuss the coding of these variables in Appendix 1 and present summary statistics in Appendix 2.

### Descriptive patterns

**Fig. 3:**
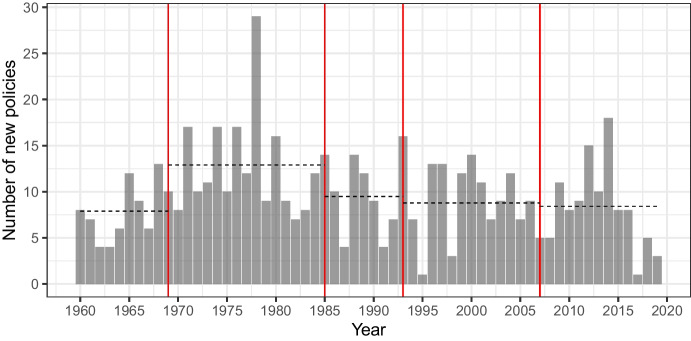
Number of new policies over time. The red, vertical lines indicate key ICJ rulings. The dashed, horizontal lines indicate average number of policies per year in each period

We begin by presenting the number of policies over time. When a state expresses a view—through domestic legislation, bilateral treaty activity, memorials submitted to courts or tribunals, or any other declaration with a preference for a method—we consider this to be a *new policy*. Making a new policy does not mean that the state’s preference changes. With a new policy, states can reiterate their support for a method they have last used by using it again. Figure 3 shows that most of the activity takes place in the second period, following the ICJ’s 1969 ruling. There were about 13 (12.9) policies per year in that period, whereas the average rate of activity is similar across the other periods, ranging from 7.9 (period 1) to 9.5 (period 3).

**Fig. 4:**
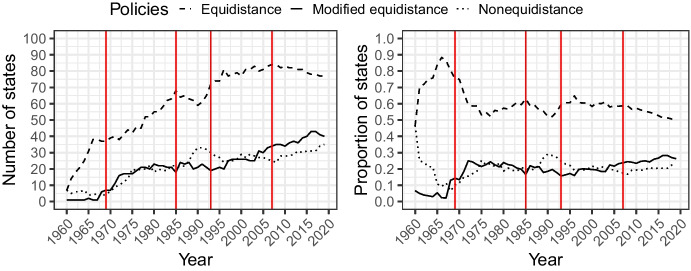
The evolution of state policies regarding continental shelf delimitation (1960–2019) in absolute numbers (left) and in proportions (right). The vertical dotted lines mark 1969, 1985, 1993, and 2007, corresponding to the four key rulings discussed above

Figure 4 presents the distribution of state policies over time, focusing on states that have clearly articulated a preference with a policy. The figures give us a first sense of the relative popularity of these three methods, both in absolute numbers and proportions.^35^

We observe that the period following the 1969 *North Sea* decision (indicated by the first vertical line), *strict or simplified equidistance* stagnates and attracts states in fewer proportions than before that decision. *Nonequidistance*, and to a lesser extent, *modified equidistance* experience an opposite trend in the same period. Both rules are promoted by the ICJ at the expense of equidistance in 1969 and 2007 (*nonequidistance*) on the one hand, and 1985 and 1993 (and more recently, from 2009 onwards) (*modified equidistance*) on the other hand. However, these do not overtake *equidistance* in popularity*,* which continues to be supported by at least half of the states with expressed policies*.* States continued to follow *equidistance* in their bilateral treaties and reiterate their support through domestic acts, even after court decisions promoting *nonequidistance* and *modified equidistance*.

**Fig. 5:**
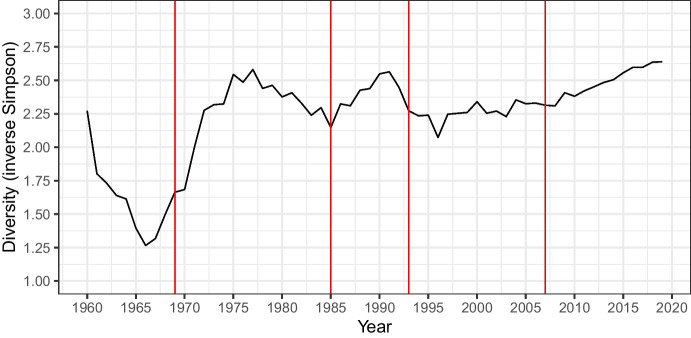
Diversity of state policies over time, as measured by the inverse Simpson index. The vertical lines mark 1969, 1985, 1993, and 2007, corresponding to the above-mentioned four key rulings

How does diversity evolve, given these distributions? Figure 5 illustrates the evolution of diversity over time, measured by the inverse Simpson index.

Diversity appears to decrease until the late 1960s/early 1970s, after which it increases significantly and stays high throughout the time period under study.^36^ Together, Figs. 4 and 5 are consistent with a defocalization process marked by the weakening of the plausible focal rule (*equidistance*) and the increase in diversity, which remains high compared to the first period.

With respect to our typology of defocalization, depicted in Fig. 1, the process does not appear to have culminated in polarization or one rule overtaking another. Instead, we observe some levelling in the sense that it is as likely for a state to have equidistance as its preferred rule as it is to have one of the two other rules. With respect to our stylized defocalization process, depicted in Fig. 2, we can note that the rules promoted by the ICJ instead of the focal rule do not manage to overtake equidistance in a way that could begin to reduce diversity. This may be an indication that court decisions are not followed as much as our stylized process assumes. A contributing factor to this may be the fact that the ICJ did not consistently promote an alternative method against equidistance but instead changed its stance on at least three key occasions, with different degrees of departure from its prior case law.

In the next section, we test our expectations about the role that incongruence and inconsistency may have played in this process as the ICJ failed to endorse a plausible focal rule, instead endorsing different alternative rules at different points in time.

### Regression analysis

**Fig. 6:**
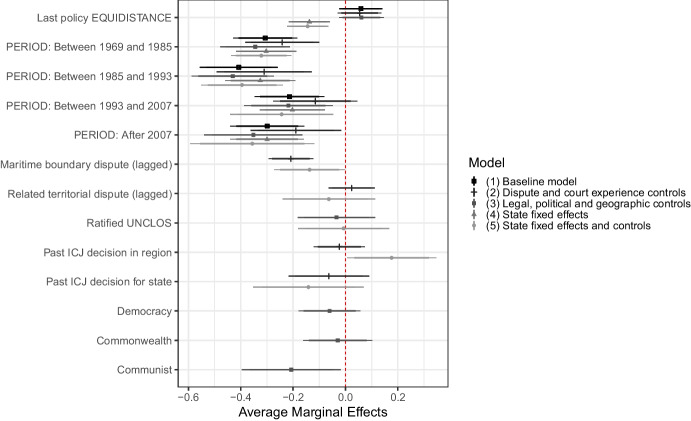
Average marginal effects calculated using five logistic regressions, whose output are reported in Appendix 5

We begin with modelling the probability of states expressing a preference for equidistance, the most plausible focal rule, across time. We run five logistic regressions that focus on state-years in which there is a new expression of a policy preference. The marginal effects calculated based on our logistic regression models are presented in Fig. 6. In each model, we interact our period variables with a dummy variable that takes the value of *1* if the state’s last expressed policy was equidistance. This is because we expect that those that have already used equidistance may be particularly advantaged by it, and may be more reluctant to change, regardless of what the court promotes.

Figure 6 shows that the probability of expressing a preference for equidistance is lower in each period after the first ICJ ruling in 1969, and the expected change in the popularity of equidistance seems to be particularly steep in the two periods following this highly incongruent decision. The difference is statistically significant and substantially important. The probability of choosing equidistance in Periods 2 and 3 are respectively about 30% and 40% less than the probability of choosing it in Period 1. We are careful not to attribute the particularly significant drop in Period 3 only to the 1985 decision. The shock of 1969, that initiates Period 2, may have effects reaching well into Period 3. The only period that is hospitable to equidistance is Period 4 (1993–2007), marked by lower levels of incongruence. Thus, it appears that higher levels of incongruence (after 1969 and 1985) are associated with an important decrease in the probability of opting for equidistance as a delimitation method (H1).

These patterns hold in the presence of various controls and state-fixed effects, except for the case of Period 2 when we control for the existence of a maritime boundary dispute. We note that the existence of a maritime boundary dispute is associated with a lower probability of expressing a preference for equidistance.^37^ Therefore, some of the decline in preferences for equidistance can be captured by the reluctance of countries with a dispute to use equidistance in Period 2. Related territorial disputes or UNCLOS ratification do not seem to have any association with the viability of equidistance. Finally, it is interesting to note that having equidistance as the last policy is associated with a lower probability of picking equidistance again in the models with fixed effects. This may be because the fixed-effects models consider only those countries that have used a policy other than equidistance at some point in time, and they are likely to switch to that policy from equidistance. We carry out a series of robustness tests with different subsets of countries, and our main results regarding equidistance still stand.^38^ Furthermore, we run two selection models with factors that can affect the making of a new policy (e.g., GDP per capita and the number of remaining boundaries to delimit) included in the selection equation, and other factors that can affect the probability of states choosing equidistance in the outcome equation. We find no evidence of selection bias.^39^

We now turn to our hypotheses about inconsistency. Going back to Fig. 6, we note that equidistance seems to be particularly less likely after an unexpectedly inconsistent decision in Period 5. While the decrease in popularity is also steep in Period 2, it is hard to rule out that incongruence or the lasting effect of 1969 is partly responsible for this. This is consistent with the idea that inconsistent decisions, especially the unexpected ones, that promote another method undermine the popularity of the plausible focal rule (H3).

**Fig. 7:**
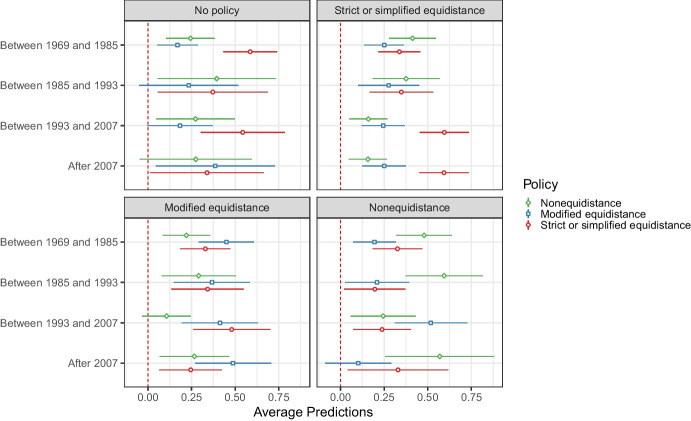
Multinomial logistic regression with interaction between the chosen policy type and previous policy

H3 also suggests that the popularity of equidistance will decrease through the following mechanism: inconsistent decisions create moves away from the plausible focal rule in favor of the promoted rule. Both modified equidistance and nonequidistance were promoted in turn by the ICJ, and this might create an increase in their attractiveness compared to equidistance in the periods following their promotion. To get at this, we run multinomial logistic regressions that have a categorical variable indicating the policy choice (equidistance, modified equidistance, and nonequidistance) as the dependent variable. The models control for whether the state in question has an ongoing maritime boundary dispute and the region that it is found in. We also consider whether states’ previous policies condition their choices. The predictions based on these models are depicted in Fig. 7.

In Fig. 7, we observe a certain pre-disposition to repeat the same policies, although this is not always the case for each period or each existing policy. Indeed, a state that reaches the last two periods with equidistance as their last policy are very unlikely to use another policy, despite contrary judicial signals. Yet there is also evidence of sensitivity to judicial signals in a way that contributes to undermining equidistance as suggested by H3. For countries with no prior policies, for instance, the rule promoted by the court seems to shape preferences. In the period between 1969 and 1985, nonequidistance appears to be more popular for countries without prior policies. This popularity extends to the period beginning in 1985, which is an in-between period where the court reiterated the importance of nonequidistance while using modified equidistance in practice. For the same group, state preferences in subsequent periods (1993–2007 and post-2007) also seem to be aligned with the court’s signals with an emphasis on modified equidistance and nonequidistance, respectively.

**Fig. 8:**
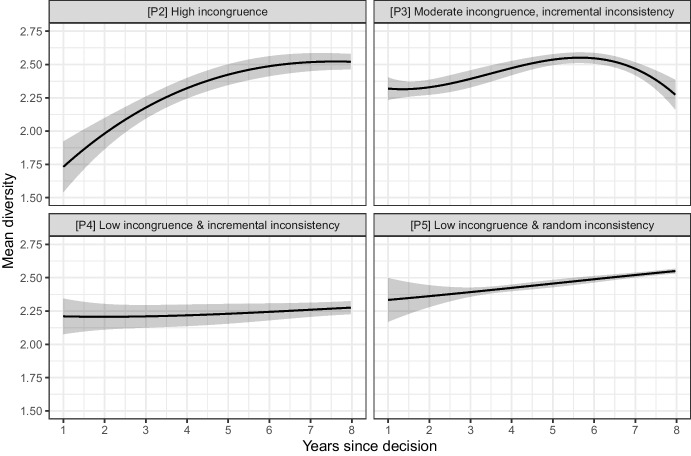
Evolution of diversity in years following each key decision. See Appendix 9 for the regression output

We now turn to the *evolution of diversity* after each key ruling, relevant to H2 and H4. We model *diversity *$\left( D \right)$ in the time periods we identified, corresponding to different combinations of incongruence and inconsistency, in interaction with the number of years passed after the ruling and its cubic polynomial. Our predictions are presented in Fig. 8. We observe that the greatest moves towards higher diversity are seen in the period after the decision that is marked by a particularly high level of incongruence (the 1969 *North Sea* ruling). Moreover, at similar incongruence levels, inconsistency that is unexpected rather than an incremental is followed by a period with a clearer increase in diversity. That being said, given our limited data points and empirical strategy, we are careful not to draw causal conclusions about these types of court interventions nor about the possible differential effects depending on the different types of inconsistency.

The patterns of policymaking in the field of continental shelf delimitation have allowed us to probe some of our expectations related to the defocalization processes. We have sought to assess the extent to which incongruence and inconsistency are related to the loss of popularity of a plausible focal rule, and found some support for this expectation in the case of incongruence. Especially high incongruence appears to be associated with a reduction in the popularity of a focal rule that had until then enjoyed a default-rule status both in treaty law and state practice. However, we find limited support for the expectations that link inconsistency to jumps in diversity through the promotion of different rules. Diversity does stay high overall, but we do not see an unambigous pattern of increase in popularity for rules right after they are promoted by inconsistent court rulings. This may be because inconsistency developed incrementally until later dates, and the effect of the initial incongruence may weigh greater than any gradual change in the case law. Yet, patterns of alternative rule adoption that accompany the recession of the plausible focal rule are more consistent with judicial signals when we control for states’ prior policy choices.

### Epilogue: Type of defocalization and the state of diversity

**Fig. 9:**
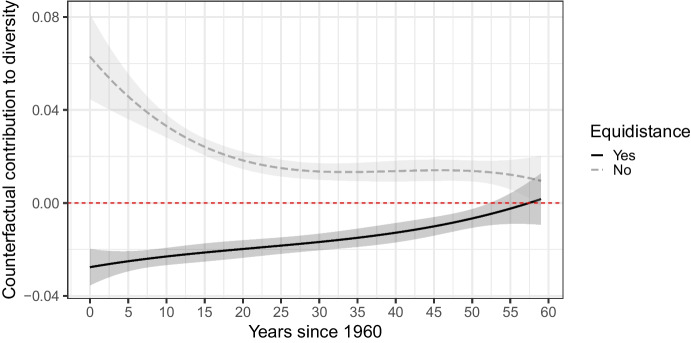
Counterfactual contribution to diversity in state policies modelled by the interaction between equidistance as a new policy and cubic polynomial for time since 1960. The regression output is reported in Appendix 9

As a final descriptive illustration, we consider whether the defocalization process culminated into a stable end point. As long as equidistance is the most popular rule, choosing it should be associated with a decrease in diversity. Once it loses its prominent status, choosing equidistance is expected to increase diversity. To probe this, we consider the counterfactual effect of different types of policies states can enact throughout the period under study. We run an OLS regression including the counterfactual contribution to diversity as the dependent variable and the choice of equidistance as a policy as the independent variable in interaction with the cubic polynomial for time since 1960. Predictions based on this model are depicted in Fig. 9.

We observe that the effect of adopting equidistance is always associated with a decrease in diversity until the very end of the period, when the policies become divided evenly across *pro-equidistance* and *pro-other methods* camps. This suggests that the defocalization process has culminated into a point that is either in the process of being *levelled* or is *polarized*, depending on whether we can group modified equidistance and nonequidistance together. We can conclude that the focal status of equidistance appears greatly undermined: by the end of our observation period, the state practice is practically divided 50–50. Moreover, diversity seems to have reached some stability—any gain made by equidistance or others can reinforce them without significantly increasing diversity.

In essence, our case shows that a defocalization process can stabilize and persist, with no policy becoming dominant, as various court signals promoted alternative policies. While our plausible focal rule was not overtaken by any other method, it remains counterbalanced by the combination of two alternative methods that came to be prefered by the ICJ and chosen by a sufficient number of states. What may also make defocalization persist is the observation that earlier policies condition later ones and some states will no longer need to make new policies, for instance, because they will have delimited all their boundaries. What is clear is that the ICJ has not said its last word, as maritime delimitation cases continue to arrive at the court’s docket.

### Endogeneity issues and alternative explanations

Our story can be challenged on endogeneity grounds: diversity in state practice may move the court rather than the other way around. Endogeneity may arise if the court issues seemingly incongruent and inconsistent decisions because of existing diversity in state practice or because it adopts a deviant view proposed by one of the parties in dispute. We believe this is not the case. First, the ICJ does not have means to survey broader state practice other than relying on its own decisions, UN resolutions, and treaty ratification information (Bradley, 2016; Choi & Gulati, 2016). Mark Weisburd notes that “[i]n some cases, [the ICJ] has reached decisions clearly inconsistent with significant and relevant state practice; in others, it has proclaimed doctrines unsupported by state behavior as rules of law” (Weisburd, 2009, 295). Second, the court is not bound to choose an argument one of the parties raises. It can find a solution that sidesteps some or all of the arguments or proposed solutions submitted by the parties.

Moreover, both of these scenarios overlook the court’s agency. Even if the court could reliably survey broader state practice, it would still be up to the court to decide how much it wants to base its decisions on that practice. The court can as easily render and justify its decisions that follow trends in state practice as those that depart from them. Similarly, the court has agency as it entertains the arguments submitted to it by parties to a dispute. Even if the court chooses to endorse one party’s position, it does not make that party responsible for the court’s decision or its consequences. At most, we can say that diversity that the court sees in state practice as well as in the arguments of parties to a dispute gives the court an *opportunity* to render incongruent and inconsistent decisions. At the end of the day, any defocalizing effect that the ruling may have is due to the choices of the court that enjoys considerable discretion.

Our account can also be challenged through alternative explanations. An obvious set of alternative explanations are provided by the UNCLOS negotiation history, with key events occurring at similar moments as court decisions. Another alternative explanation may be the joining of new states to the system, mainly throughout the 1960s and 1970s, when the most striking variation in diversity occurs. We entertain these in turn.

The process surrounding the UNCLOS negotiations could provide similar cutoff points to what we have. In 1967, the UN General Assembly called for setting up an Ad Hoc Committee to Study the Peaceful Uses of the Sea-Bed and the Ocean Floor beyond the Limits of National Jurisdiction (the Seabed Committee). Two years later, in 1969, the General Assembly asked the Secretary General to survey state views on the need for an international conference on the law of the sea. At the end of 1970, the General Assembly decided to convene such a conference. The conference convened in 1973 and lasted until 1982, when UNCLOS was signed. UNCLOS entered into force in 1994 (United Nations, 2008). Most of these dates fall very close to our suggested cut-off points.

With regards to the preparation period before 1973, the work of the Seabed Committee and the announcement of a possible conference may have increased the salience of maritime boundary making, thereby driving unilateral positions, boundary agreements, and reactions thereto. It is plausible that this increased activity drives some deviations from strict equidistance and fosters diversity. However, delimitation rules were not a key issue in the preparatory work undertaken by the Seabed Committee. Its reports through 1972 do not involve any specific discussion of delimitation methods, instead focusing on the definition of territorial sea and high seas, and the seabed floor’s possible uses.^40^ Thus, it is doubtful that the particular work of the Seabed Committee drove states to adopt new policies that could help explain our results.

Regarding the negotiations themselves (1973-1982), they may have led to diversity by encouraging states to position themselves vis-à-vis other states and available (informal or official) draft treaty texts. This may well be the case, but there are reasons to doubt that negotiations on their own could drive this process. This is primarily because they did not take place in a vacuum, but in the context of the prior treaty law (the 1958 CSC) and a court decision that contradicted it (the ICJ’s 1969 *North Sea* decision). States did not come up with additional delimitation rules other than the alternatives thoroughly entertained by the ICJ in *North Sea*. Commentators agree that the main contention during the negotiations on delimitation rules was about whether equidistance should have a default status (like in 1958) or equity should be the ruling principle, with equidistance considered as just an option (like in 1969) (Buzan, 1981; Lando, 2019; Tanaka, 2019).

Moreover, it is hard to explain why the particular divisions emerged during the negotiations without considering the ICJ’s key role. Concerning the continental shelf, in particular, which was discussed by Negotiation Group 7, two factions emerged with roughly equal force, namely “median line states” of 20 participants and “equitable principle states” of about 22 to 27 participants (Buzan, 1980, 204). It would not be a stretch to suggest that the 1969 *North Sea* ruling provided states a justified basis to argue against giving equidistance a default rule status. The influence of that ruling on the negotiations and the developments after the UNCLOS’s adoption is well noted by scholars and practitioners alike (Blecher, 1979; Buzan, 1980; Fawcett, 1977). In fact, many negotiators were legal professionals who were well-versed in the ICJ jurisprudence.^41^

Still, we cannot rule out the possibility that the negotiation setup might have exposed more states to these alternatives and led them to make choices that increased diversity. For instance, noting that pro-equidistance and pro-equitable principles camps were almost equally divided, states without a clear position may have considered that either was acceptable, which may have further eroded the almost-default status that equidistance once enjoyed. Also likely is the possibility that negotiatons accelerated the process of states’ policy-making and the changes in diversity. The negotiation process is thus likely to have contributed to defocalization, without being its main instigator.

Another alternative explanation may be the fact that several new states joined the system over the years that are under study here, and they may have had something to do with the changing popularity of equidistance and associated upward move in diversity. These states may have contributed to increasing diversity especially in the 1960s and 1970s when countries such as the UK, France, and Portugal saw most of their former colonies and dependencies become independent. If these newly independent states adopt policies different from their former colonizers or parent states, and if the latter group favored equidistance during the same periods, the popularity of equidistance may decrease, and diversity could increase in a way very close to what we observe.^42^

We address this alternative explanation in Appendix 7. We find that regardless of the policy held by their parent state at the time of their independence, newly independent states appear more likely to adopt equidistance as their preferred policy. Insofar as the interventions of the ICJ make equidistance less popular and increase diversity, newly independent states make it more difficult for us to observe this effect because they adopt equidistance more frequently than other states. Thus, the main patterns we observe about the reduction of equidistance’s popularity and the increase in diversity cannot be due to the work of new states. If anything, these new states appear to have slowed down the drop in the popularity of equidistance.

While other factors—such as earlier signs of diversity and divisions during treaty-making negotiations—may have played a role, we believe that the defocalization process we observe cannot be explained without a consideration of the ICJ’s key role, especially in setting the stage for the rivalry between equidistance and equity. Had the ICJ not intervened in the way it did, states may have still abandoned equidistance and diversity may have increased. Yet, especially had it ruled that equidistance was the default rule in *North Sea,* the state practice may have eventually converged around equidistance as a default rule, which could have resulted in a more homogeneous state practice, despite the existence of some possible holdouts. Our contention is that, having the unique authority to declare what the law is, whenever it is asked, is a key factor endowing courts with a power to shape expectations and policies in a way one-off multilateral conventions or isolated trends in state practice cannot (Krisch & Yildiz, 2023, 13–14).

## Conclusion

In this article, we propose *defocalization* as a new concept that captures the erosion of a plausible focal rule and the resulting increase in policy diversity. In particular, we highlight the role international courts play in this process. While courts are commonly assumed to have a key role in providing focal rules, we suggest that they are equally well positioned to do the opposite: providing alternative rules that dilute the attractiveness of any possible focal rule and impeding policy convergence around it. We suggest that courts may have a *defocalizing effect* when they render decisions that are incongruent with existing treaty law and practice and inconsistent with their earlier decisions.

We test our expectations about the operation of this effect in the field of the continental shelf delimitation. We identify four key rulings, each representing a different combination of incongruence level and inconsistency type. The clearest association we have is that between high incongruence on the one hand and important drops in the popularity of the focal rule and increase in diversity on the other hand. Inconsistency, for its part, may play some role in maintaining diversity at high levels. While the evidence is overall consistent with our expectations, our methods do not allow us to attribute a causal effect to court rulings.

The ICJ strongly went against equidistance in 1969, which is followed by a period when equidistance’s popularity significantly dropped. In its subsequent decisions, the ICJ continued to promote other rules at the expense of equidistance, sometimes taking unexpected turns. We believe that these rulings have played a role in chipping away at equidistance’s popularity and preventing it from becoming dominant. Surprisingly, equidistance continued to attract about half of the states as the process unfolded. There is some evidence that the popularity of rules is sensitive to the ICJ’s signals, although prior policies also appear to condition future ones. While the ICJ’s effect is hard to untangle from other contemporary developments—such as UNCLOS negotiations— its role is hard to overlook in defining the contours of the division in state practice and giving legal justification for deviating from equidistance.

A key limitation in our study is that we lack data points that would have allowed us to better establish the influence of different levels of incongruence and different types of inconsistency in the defocalization process. Although we had different levels of incongruence, we do not have enough cases as clear examples of sudden or unexpected inconsistency. A valuable avenue for future research is in areas where the case law is sufficiently large to make valid inferences concerning the possibly varying effects of different types of inconsistency.

Nevertheless, our study makes several key contributions to the existing literature on international courts and regime complexity. First, we offer *defocalization* as a new concept to understand the way in which international courts may ambiguate rules and contribute to the accumulation of complexity over time. This concept helps us assess international courts’ varied impact on world politics and better distill conditions under which courts can be expected to foster discord rather than policy harmonization. In addition, we introduce a typology of defocalization identifying its various possible manifestations. We believe that this concept and the accompanying typology can serve to build intuition about the causes and consequences of policy discordance and complexity in other fields. Building on these, future studies can attempt to uncover the net effect of courts through both process tracing and appropriate methods for causal inference.

Second, our key finding regarding incongruence’s plausible effect on defocalization has important implications for the politics of international courts. While many may be more concerned about courts’ consistency, it is those rulings that are incongruent that can be more and durably damaging for policy harmonization. Conversely, this suggests that the path to focalization can be more successful if the court is able to assess the state of state practice and contribute to converging trends. Therefore, courts’ main attributed task to clarify the law and resolve disputes should not be detached from the utility they may find in gauging broader trends in state practice. One caveat is that focalization around emerging trends may not always be morally desirable, especially in the context of authoritarian influences on international law (Ginsburg, 2020).

Third, we identify mixed messaging, caused by *incongruence* and *inconsistency* over time, as a plausible driver for building and sustaining complexity. While it is argued that regime complexity does not need to “portend disorder” (Alter, 2022, 393), much of this scholarship shares the implicit assumption that policy overlap inevitably opens the door for different institutions to offer diverse solutions in order to obtain advantages in situations of inter-institutional competition. We suggest here that the important factor is not so much the multiplicity of institutions, rather the multiplicity of policy solutions that can be the work of a single institution over time.

Although we test and illustrate our theory in the domain of continental shelf delimitation, our argument has implications for other judicialized fields, such as trade, use of force, and human rights. As cases international courts treat are varied, it is always possible that a ruling will deviate from the existing treaty law or established precedent. We argue that the fundamental question is that of elucidating the conditions under which elements of complexity lead to policy discordance rather than reinforcing each other to provide more predictability and certainty. This question is also of clear policy relevance at a time when increasingly growing regime complexes are called onto address global cooperation problems concerning, for instance, climate change and global health.

## Supplementary Information

Below is the link to the electronic supplementary material.

Supplementary file1 (PDF 334 kb)

Supplementary file2 (ZIP 112225 kb)

## Data Availability

The data and code used to generate the findings of this paper are available online (see, Supplementary Information).

## Footnotes

1 Thomas Schelling introduced the concept of *focal point* to capture the idea that some solutions impose themselves as the obvious ones for actors to coordinate their behavior. A focal point works if it is “prominent,” “conspicuous,” and “unique” enough that no other solution can be as easily justified; it works even when the resulting particular outcome is more advantageous to one side than the other (Schelling, 1960).

2 While a focal rule may emerge and be recognized as such independently from any judicial activity, courts have been said to be ideally placed to facilitate the formation of focal rules. Our argument, however, is that courts similarly possess a unique ability to dilute emerging focal rules by endorsing alternative rules. We make no claim as to whether courts act with an *intention* to focalize or defocalize. It may be that judges come with different convictions, and the unexpected turns we may observe could be due to changes on the bench (Kauppi & Madsen, 2014; Yildiz & Yüksel, 2024). Alternatively, the mixed messaging could be a side-effect of the interaction between judicial lawmaking and the treaty-making processes. Courts might sometimes attempt to create exceptions to rigid treaty rules (Búzás & Graham, 2020) and sometimes might fill in the gaps left in treaties (Carrubba & Gabel, 2017). We simply focus on the *effects* of court decisions, regardless of judges’ objectives (see, Verdier & Voeten, 2014, 420), leaving future studies to tackle this important question.

3 Continental shelf as a maritime zone is a relatively recent creation—claimed for the first time by the United States with the 1945 Truman Proclamation, before becoming a common practice and getting codified in the 1958 Continental Shelf Convention (CSC).

4 Inconsistency has previously been raised as an issue for *ad hoc* tribunals, such as those dealing with investor-state disputes (Franck, 2005).

5 We find it useful to distinguish between *focal rules* and *focal points*. Our distinction follows Baker (1993, 164–65), where *focal rules* refer to simple rules that can help actors reach a coordinated equilibrium even in the absence of explicit bargaining. *Focal points* refer to a specific solution that stands out to the actors for coordinating their behavior. The actors may use the *focal rule* to get there, but do not need to. A *focal rule* (for example, *uti possidetis iuris*, whereby borders between newly independent states follow old administrative borders) may give prominence to certain outcomes that are consistent with it but may often not be enough to establish a unique *focal point* (to continue our example, if the administrative borders have changed, or are drawn differently on maps held by different states).

6 International lawyers echo this sentiment in the fragmentation/legal pluralism debate (Berman, 2012; Koskenniemi, 2006).

7 There are few exceptions; see, e.g., Eilstrup-Sangiovanni (2022), Fioretos & Hofmann (2024) and Hofmann & Yeo (2024).

8 We use this typology to build intuition around this new concept of focalization. In the empirical section, we refer to it mainly in a descriptive manner. We believe that the typology can usefully guide future studies that trace potential episodes of defocalization across different cases to assess when and why each process takes the course and culminates in an outcome related to one or the other type of defocalization.

9 Court rulings are among the subsidiary sources of international law under Article 38 of the ICJ Statute.

10 The type of inconsistency goes beyond the different applications of a rule due to factual differences surrounding cases. We specifically focus on the inconsistent takes on principles of general application rather than apparent inconsistencies in decisions resulting from factual idiosyncrasies.

11 Case Concerning Military and Paramilitary Activities in and Against Nicaragua [Judgment] (1986), para. 195.

12 Now the voices denouncing self-defense against non-state actors increased with the African Union’s recent announcement of its common position, which maintains that the right of self-defense can only be invoked when responding to an armed attack attributable to a state (Helal, 2024).

13 *Legal Consequences of the Construction of a Wall in the Occupied Palestinian Territory* [Advisory Opinion] [2004], para. 139.

14 *Armed Activities on the Territory of the Congo (Democratic Republic of the Congo v. Uganda)* [Judgment] [2005], paras. 146, 147.

15 *Öcalan v. Turkey*, application no. 46221/99, ECHR[GC] (12 May 2005), para. 165.

16 *Al-Saadoon and Mufdhi v. the United Kingdom*, application no. 61498/08, ECHR (2 March 2010), para. 120.

17 *Schalk and Kopf v. Austria*, application no. 30141/04, ECHR (June 24, 2010).

18 Only in 2015, in *Oliari v. Italy*, the ECtHR pronounced that states should offer some sort of legal recognition of same-sex marriage*,* though this issue was quite contextualized. *Oliari v. Italy*, application nos. 18766/11, 36,030/11, ECHR (July 21, 2015).

19 At the time of a more recent case, eighteen states (Austria, Belgium, Denmark, Finland, France, Germany, Iceland, Ireland, Luxembourg, Malta, the Netherlands, Norway, Portugal, Slovenia, Spain, Sweden, Switzerland, and the United Kingdom) allowed marriage between persons of the same sex, while twelve other states (Andorra, Croatia, Cyprus, the Czech Republic, Estonia, Greece, Hungary, Italy, Liechtenstein, Monaco, Montenegro, and San Marino) at the time allowed alternative forms of partnership to marriage for same-sex couples. *Fedotova and Others v. Russia*, application nos. 40792/10, 30538/14, 43439/14, ECHR[GC] (January 17, 2023), para 66.

20 See, for example, dissents of the Polish and Russian judges in *Fedotova and Others v. Russia* (2023), using *Schalk and Kopf v. Austria* to argue that Russia cannot be faulted for not recognizing same-sex couples.

21 We explain the construction of this measure—the inverse Simpson index—further below.

22 *North Sea Continental Shelf (Germany/Denmark the Netherlands)* (Judgment) [1969] ICJ Report 3.

23 We exclude two cases where the Court did not reach a decision regarding the delimitation method: *Aegean Sea Continental Shelf Case (Greece v Turkey)*, where the ICJ ruled that it lacked jurisdiction; and *Croatia v Slovenia,* where an arbitral tribunal made no assessment regarding the delimitation method.

24 *Nicaragua v Honduras* (Judgment) [2007] ICJ Report 2.

25 These decisions are recapitulated below, in Table 2, where we present our operationalization of incongruence and inconsistency.

26 We discuss these further in the context of UNCLOS negotiations as an alternative driver of diversity in state practice.

27 The *pro-equidistance* group included Bahamas, Barbados, Canada, Colombia, Cyprus, Denmark, Gambia, Greece, Guyana, Italy, Japan, Kuwait, Malta, Norway, South Yemen, Spain, Sweden, the United Arab Emirates, the United Kingdom, and Yugoslavia. The *pro-equitable principles* group comprised Algeria, Argentina, Bangladesh, Benin, Congo, France, Iraq, Ireland, Ivory Coast, Kenya, Liberia, Libya, Madagascar, Mali, Mauritania, Morocco, Nicaragua, Nigeria, Pakistan, Papua New Guinea, Poland, Romania, Senegal, Syria, Somalia, Turkey, and Venezuela. Official records of the UNCLOS III, A/CONF.62/RCNG/1 (1978), 124–125.

28 According to Article 83(1), continental shelf delimitation is to be “effected by agreement on the basis of international law, as referred to in Article 38 of the Statute of the ICJ, in order to achieve an equitable solution.”

29 *Libya/Malta* (Judgment) [1985] ICJ Report 13, para. 44.

30 *Jan Mayen (Denmark v Norway)* (Judgment) [1993] ICJ Report 38.

31 *Qatar v Bahrain* (Judgment) [2001] ICJ Report 40, para. 175–176. This model was then developed further in *Black Sea (Romania v Ukraine)* (Judgment) [2009] ICJ Report 61, para. 116.

32 *Nicaragua v Honduras* (Judgment) [2007] (Dissenting Opinion of Judge Torres Bernárdez, para 122).

33 To the best of our knowledge, DOALOS does not host treaties that are not in force.

34 For *equidistance*, we had “equidistance”, “equidistant”, “at equal distance”, “median line”, “midpoint”, “median”, “middle line”; for *modified equidistance*, we looked for “proportionality”, “relevant circumstances”, “special circumstances”; and for *nonequidistance*, we used “equity”, “equitable”, “fair”, “just”, “parallel of latitude”, “bisector”, “natural prolongation”. Our algorithm also highlighted terms like “azimuth” as potentially indicative of a delimitation method. For more, see (Yildiz & Yüksel, 2022).

35 The figure ignores country-years where we have not been able to identify any recent preference. In years without a new policy statement, state’s policy is the most recently adopted one.

36 It is possible that global diversity scores mask a possible homogeneity of practices at the regional level. If regional practices coalesce around specific rules, diversity may be low at the regional level but still appear high globally. We assess this possibility in Appendix 3, which allows us to rule it out.

37 The reluctance or inability of states in dispute to choose strict equidistance is not surprising. If the states could draw their boundary following equidistance, they probably would not have been in a dispute in the first place.

38 These are reported in Appendix 6.

39 See Appendix 8.

40 E.g., UNGA, Report of the Committee on the Peaceful Uses of the Seabed and the Ocean Floor Beyond the Limits of National Jurisdiction, No. 22 (A/7622) (1969); UNGA, Report of the Committee on the Peaceful Uses of the Seabed and the Ocean Floor Beyond the Limits of National Jurisdiction, No. 21 (A/8721) (1972).

41 For example, one of the delegates that Federal Republic of Germany sent was Günther Jaenicke, who was none other than the agent of that state in the *North Sea* case (United Nations, 2012, 39).

42 We are grateful to a reviewer for pointing out this possibility.
