# Supplementary material for: The defocalizing effect of international courts: Evidence from maritime delimitation practices
Source: Rev Int Organ. 2024 Jun 29;20(4):825–61. doi: 10.1007/s11558-024-09545-4 (PMC12727788; doi:10.1007/s11558-024-09545-4)
Supplement: Supplementary file 1 — Supplementary file1 (PDF 334 kb) [file 11558_2024_9545_MOESM1_ESM.pdf]

Online Appendix

The Defocalizing Effect of International Courts: Evidence from Maritime Delimitation Practices

Author(s)*

January 2024

*Affiliation(s)

1 Coding of control variables

The information on maritime boundary disputes and resource (hydrocarbon) activity is obtained by aggregating information based on each state's dyadic maritime activity, using a dataset of maritime boundary relations with dyad-year observations (Yüksel 2019). *Maritime boundary disputes* are actively pursued disagreements between two states over the location of their common maritime boundary. *Related territorial disputes* are disputes over land territory with clear implications for the maritime boundary that could be drawn between two states. These two variables take a value of 1 if a state concerned is involved in a maritime boundary dispute or a related territorial dispute in at least one of its dyadic relations in a given year. From the same dataset, we calculate the *number of dyadic boundaries* (the total number of states a state has to negotiate to draw common maritime boundaries), the *number of fully delimited dyadic boundaries*, and the *proportion of dyadic boundaries that are fully delimited.*

For UNCLOS ratification dates, we rely on data made public by the UN.[1] A state is coded as having ratified UNCLOS from the year in which ratification takes place and until the end of the time period. Our *Democracy*, *Commonwealth*, and *Communist/Socialist state* variables are taken from (Bjørnskov and Rode 2020). Finally, our *GDP per capita* variable comes from the World Bank's World Development Indicators. We have obtained the *Democracy*, *Commonwealth*, *Communist/Socialist state*, and *GDP per capita variables* from the Quality of Governance time-series data (Teorell et al. 2023). The data on the *legal system* comes from (Powell and Mitchell 2007).

2 Descriptive statistics

Table 1 presents summary statistics for the numerical variables that appear in our main tests.

Statistic	N	Mean	St. Dev.	Min	Max
New policy	8,915	0.066	0.248	0	1
Last policy EQUIDISTANCE (lagged)	6,254	0.584	0.493	0	1
Commonwealth	8,193	0.295	0.456	0	1
Communist	8,193	0.085	0.279	0	1
Democracy	8,193	0.514	0.500	0	1
Ratified UNCLOS	8,915	0.419	0.493	0	1
Number of total dyadic boundaries	7,627	4.423	3.741	1	36
Number of fully delimited dyadic boundaries	7,627	0.842	1.540	0	15
Proportion of fully delimited dyadic boundaries	7,627	0.182	0.280	0.000	1.000
GDP per capita (current US dollar)	7,438	7,973.636	15,270.520	12.787	203,266.900
GDP per capita (current US dollar) (logged)	7,438	7.731	1.670	2.548	12.222
Years since last new policy	8,751	14.159	12.607	1	59
Any maritime boundary dispute	7,628	0.385	0.487	0	1
Any related territorial dispute	7,628	0.445	0.497	0	1
Any ICJ decision for the state	8,915	0.066	0.249	0	1
Any ICJ decision for the region	8,915	0.495	0.500	0	1
When New policy = 1					
EQUIDISTANCE as new policy	585	0.526	0.500	0	1
Diversity (inverse Simpson index)	585	2.249	0.316	1.266	2.639
Annual change in diversity (inverse Simpson index)	575	0.009	0.130	−0.471	0.314
Counterfactual contribution to diversity (inverse Simpson index)	585	0.0002	0.027	−0.140	0.080

Table 1: Descriptive statistics.

[1] See here: https://treaties.un.org/pages/ViewDetailsIII.aspx?src=TREATY&mtdsg_no=XXI-6&chapter=21&Temp=mtdsg3&clang=_en.

3 Regions and region-based figures for distribution of policies and diversity

The regions used are based on the regional division found in the Authoritarian Regimes Dataset (Hadenius and Teorell 2007; Teorell et al. 2023), whose classification groups together geographically proximate states except for Australia and New Zealand that are grouped together under “Western Europe and North America”. We have significantly modified this classification so that it follows maritime proximity more closely.

We began by merging “Latin America” and “The Caribbean” into “The Americas”, “East Asia”, “South-East Asia”, “South Asia”, and “The Pacific” into “Asia and the Pacific”. We left “Sub-Saharan Africa” as is. We then divided up “Western Europe and North America”, assigning Australia and New Zealand to “Asia and the Pacific”, the United States and Canada to “The Americas”, and the rest of the members to a newly created group called “Europe, incl. post-Soviet”, which also included all the members of the “Eastern Europe and post-Soviet”. Then, we moved states that bordered the Mediterranean or the Adriatic to a group called “Mediterranean and the Middle East”, which, in addition to the European states bordering the Mediterranean, included all the members of the original “North Africa and the Middle East” group.

Finally, we assigned states that were unassigned in the original dataset as follows:

- Cook Islands and Niue to “Asia and the Pacific”;
- German Democratic Republic and Soviet Union to “Europe, incl. post-Soviet Union”;
- Yugoslavia to “Mediterranean and the Middle East”;
- and South Africa to “Sub-Saharan Africa”.

The result is a more or less even distribution of states across regions, as Figure 1 below depicts:

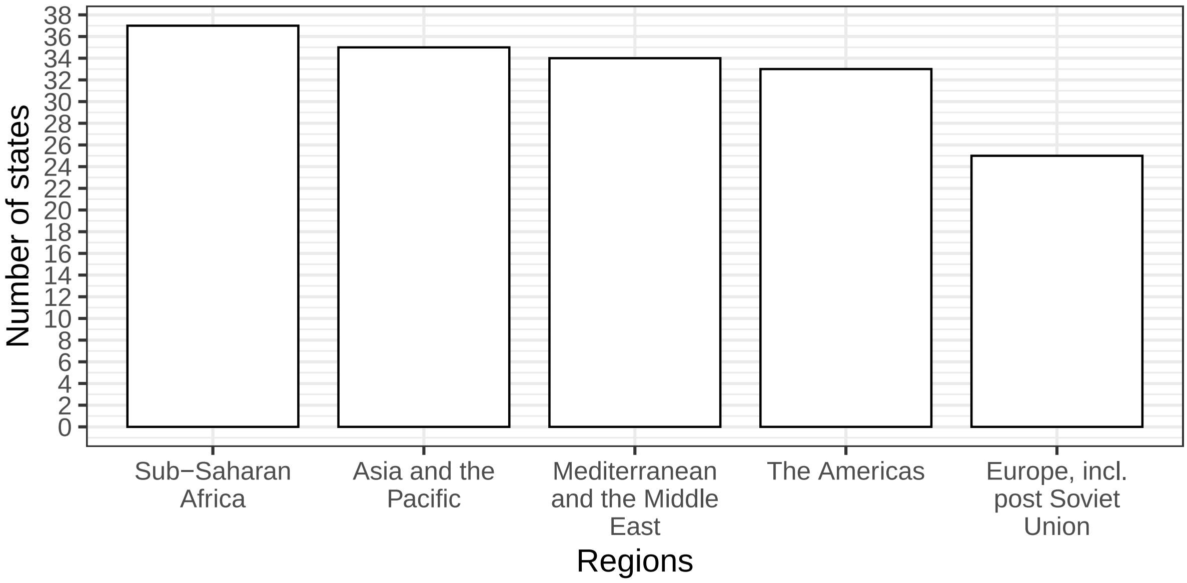

Figure 1: Number of states across modified geographic regions.

In Figures 2 and 3 below we reproduce our main descriptive figures with the distribution of policies and the corresponding diversity scores broken down by regions.

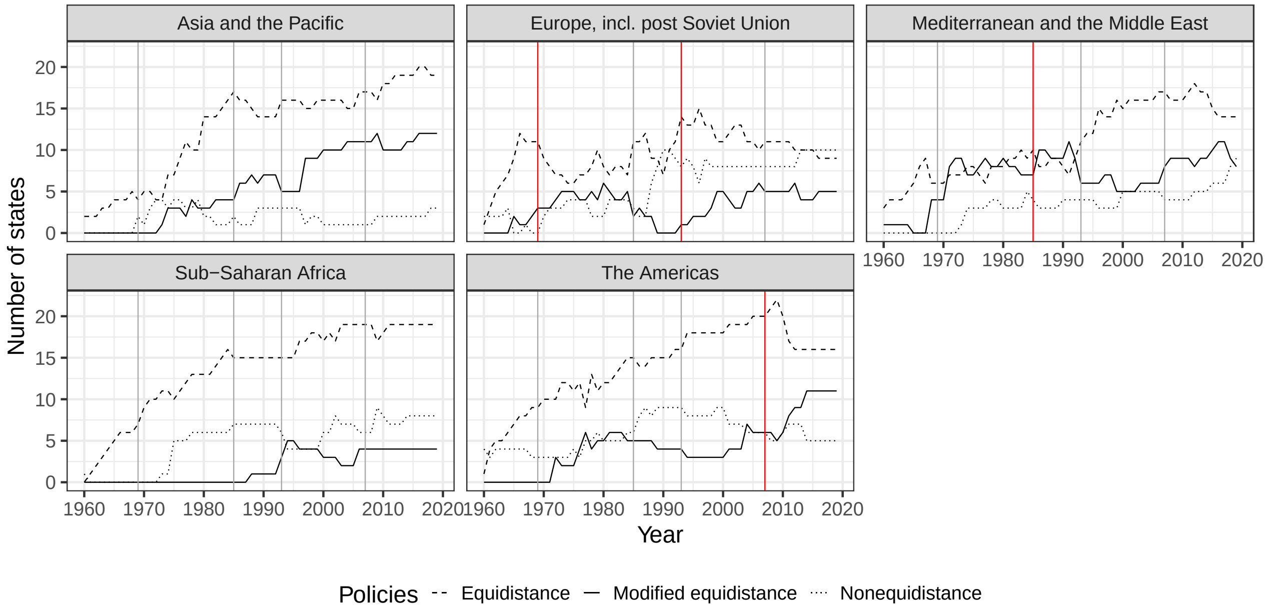

Figure 2: Distribution of policies over time by region. Vertical lines indicate key ICJ decisions. When the decision concerns states in a particular region, the lines are in red.

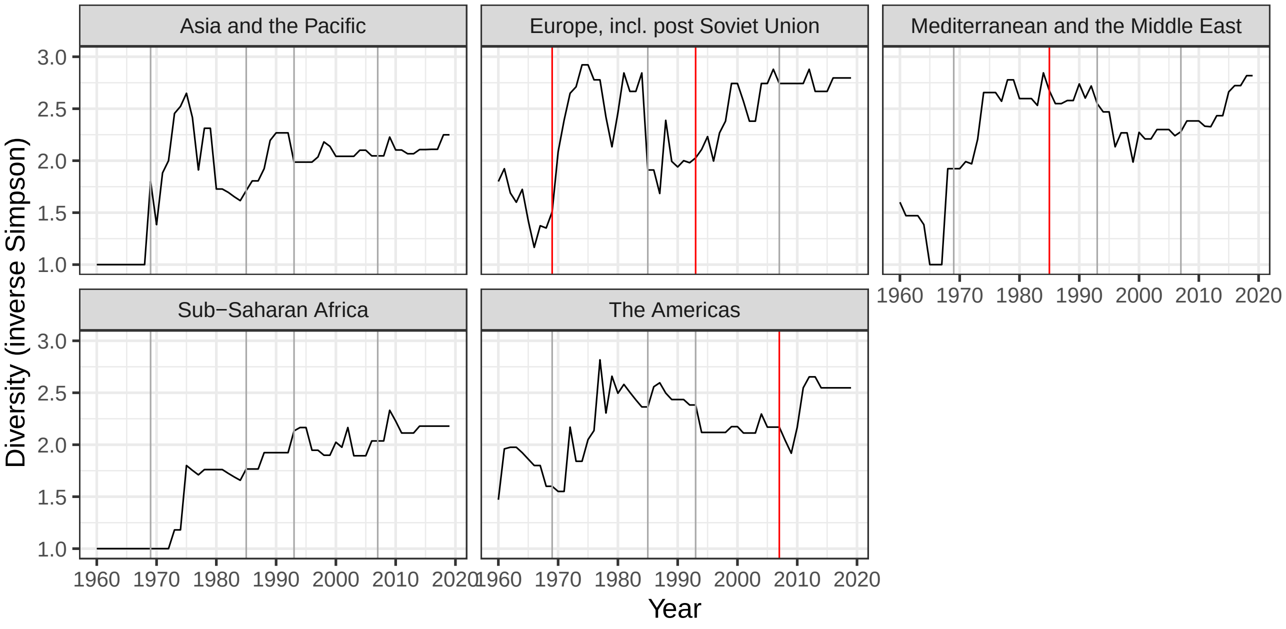

Figure 3: Evolution of diversity over time by region. Vertical lines indicate key ICJ decisions. When the decision concerns states in a particular region, the lines are in red.

4 Stylized incongruence and inconsistency

We create our stylized figures of incongruence and inconsistency with simulations that rely on an initial distribution of policies of 90 for the most popular rule and 30 each for the two others. In one scenario, the court endorses one of the rules with 30 followers at time 1 and does nothing thereafter. In the other scenario, the court endorses one of the rules with 30 followers at time 1, and the other with about 30 followers (i.e., the least popular one) at time 15. The changes in the distribution of policies are obtained by running a simulation that has a portion of the actors decide on a new policy each year (with each actor having about a 7% probability of putting forward a new policy), and the choice of the policy is affected only by their current policy and the policy promoted by the court. We defined the probabilities of various options as follows:

- If the state has already adopted the policy promoted by the court:
  - Repeat current policy: 60%
  - Switch to another policy: 20%, 20%
- If the state has not adopted the policy promoted by the court:
  - Repeat current policy: 20%
  - Switch to the policy promoted by the court: 60%
  - Switch to the third policy: 20%

We have kept the probability at which any actor adopts the policy promoted by the court quite high for illustrative purposes. The higher this is, the quicker the changes in distributions (one policy overtaking another, or diversity stabilizing after an inconsistent court decision) should be. We illustrate this in a scenario where the probabilities are defined as follows:

- If already adopted the policy promoted by the court: Repeat current policy: 84%, Switch to another policy: 8%, 8%
- If not adopted the policy promoted by the court: Repeat current policy: 8%; Switch to the policy promoted by the court: 84%; Switch to the third policy: 8%

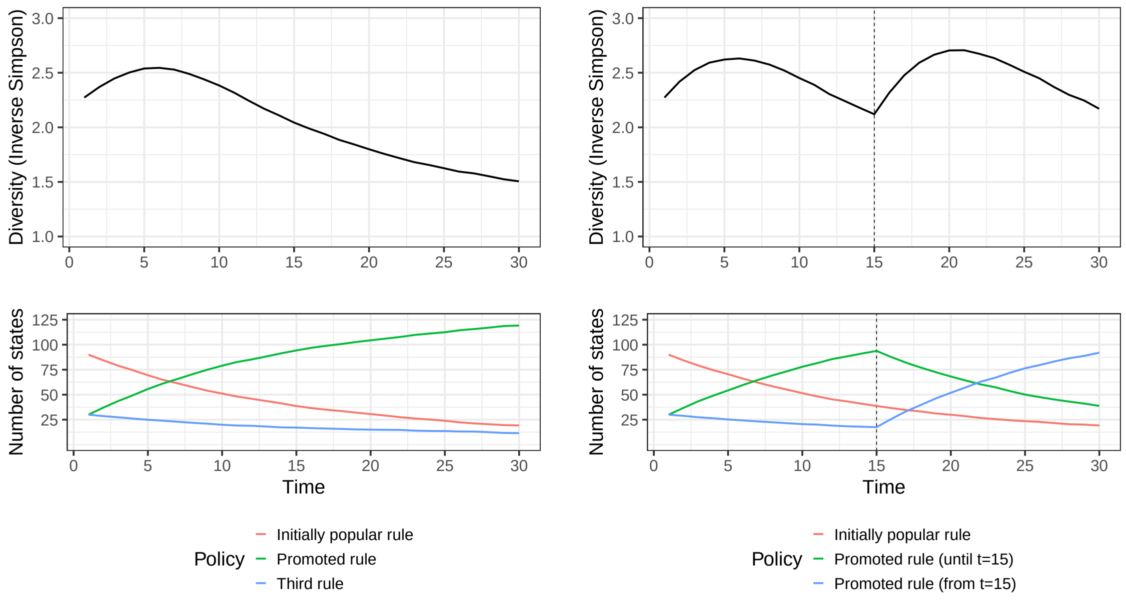

Figure 4: Alternative stylized figures showing the relationship between distribution of policies and diversity as well as how distributions and diversity score may evolve over time if states are particularly likely to adopt the policy promoted by the court.

5 Regression output for the main results

Regression output for the main models are reported in Table 2. In models 4 and 5, where state-fixed effects are used, we do not report the fixed-effects coefficients.

	Dependent variable:				
	Equidistance				
	(1)	(2)	(3)	(4)	(5)
Last policy EQUIDISTANCE	−0.804	−0.423	−0.765	−1.136	−1.105
	(0.584)	(0.615)	(0.683)	(0.829)	(0.891)
PERIOD: Between 1969 and 1985 [P2] (ref. Before 1969)	−1.602***	−1.142**	−1.867***	−2.283***	−2.537***
	(0.429)	(0.444)	(0.494)	(0.818)	(0.937)
PERIOD: Between 1985 and 1993 [P3]	−2.221***	−1.677***	−2.571***	−2.552***	−3.256***
	(0.495)	(0.540)	(0.573)	(0.881)	(1.061)
PERIOD: Between 1993 and 2007 [P4]	−1.619***	−0.974*	−1.628***	−1.653*	−2.097*
	(0.464)	(0.501)	(0.577)	(0.847)	(1.182)
PERIOD: After 2007 [P5]	−2.289***	−1.494***	−2.556***	−2.488***	−2.930**
	(0.520)	(0.571)	(0.658)	(0.944)	(1.396)
Last policy EQUIDISTANCE*P2	0.517	0.168	0.528	−0.029	−0.147
	(0.658)	(0.690)	(0.757)	(0.937)	(0.982)
Last policy EQUIDISTANCE*P3	0.998	0.715	1.312	0.221	0.264
	(0.773)	(0.815)	(0.876)	(1.114)	(1.163)
Last policy EQUIDISTANCE*P4	1.480**	1.078	1.283	0.124	0.111
	(0.694)	(0.728)	(0.785)	(1.009)	(1.053)
Last policy EQUIDISTANCE*P5	2.253***	1.551**	2.109**	0.502	0.196
	(0.730)	(0.780)	(0.824)	(1.063)	(1.158)
Maritime boundary dispute (lagged)		−0.952***			−0.991*
		(0.215)			(0.512)
Related territorial dispute (lagged)		0.107			−0.460
		(0.205)			(0.653)
Past ICJ decision for state		−0.290			−1.024
		(0.360)			(0.786)
Past ICJ decision in region		−0.106			1.273**
		(0.227)			(0.641)
Ratified UNCLOS			−0.156		−0.053
			(0.344)		(0.640)
LEGAL SYSTEM: Common (ref. Civil)			0.616*		
			(0.328)		
LEGAL SYSTEM: Islamic			−0.815**		
			(0.406)		
LEGAL SYSTEM: Mixed			−0.165		
			(0.407)		
Democracy			−0.278		
			(0.277)		
Commonwealth			−0.135		
			(0.306)		
Communist			−0.947**		
			(0.447)		
REGION: Europe, incl post-Soviet Union (ref. Asia and the Pacific)			−0.360		
			(0.345)		
REGION: Mediterranean and the Middle East			0.342		
			(0.358)		
REGION: Sub-Saharan Africa			0.803**		
			(0.381)		
REGION: The Americas			0.004		
			(0.314)		
Dyad-fixed effects	No	No	No	Yes	Yes
Observations	575	556	544	575	556
Log Likelihood	-373.231	-349.051	-340.820	-237.476	-228.258
Akaike Inf. Crit.	766.461	726.101	723.640	796.952	768.515
Note:	*p<0.1; **p<0.05; ***p<0.01 Robust standard errors clustered by state are in parentheses.				

Table 2: Logistic regression models with expressing a preference for equidistance as the binary response variable. Only state-years where a state makes a new policy are considered.

6 Robustness checks with subsets of states

We report in Table 3 the results of a series of robustness checks that focus on the interaction between last policy being equidistance and the period, with expressing support for equidistance as the response variable.

Models 1 and 4 include only those states that have made at least one new policy before 1969. Models 2 and 5 include states that made at least one policy other than equidistance. Models 3 and 6 exclude outliers in terms of policy activity, which are defined to be those states that made more than 11 policies.

	Dependent variable:					
	Equidistance					
	(1)	(2)	(3)	(4)	(5)	(6)
Last policy EQUIDISTANCE	−0.804	−0.463	−1.580**	−1.091	−1.136	−2.344**
	(0.589)	(0.594)	(0.684)	(0.763)	(0.805)	(1.041)
PERIOD: Between 1969 and 1985 [P2]	−2.125***	−1.573***	−1.545***	−2.492***	−2.283***	−2.231**
	(0.484)	(0.448)	(0.460)	(0.782)	(0.795)	(0.974)
PERIOD: Between 1985 and 1993 [P3]	−2.557***	−2.148***	−2.391***	−2.623***	−2.552***	−2.513**
	(0.599)	(0.524)	(0.547)	(0.875)	(0.856)	(1.050)
PERIOD: Between 1993 and 2007 [P4]	−1.846***	−1.652***	−1.580***	−2.051**	−1.653**	−1.367
	(0.598)	(0.492)	(0.499)	(0.814)	(0.824)	(1.012)
PERIOD: After 2007 [P5]	−2.944***	−2.114***	−2.107***	−3.193***	−2.488***	−2.054*
	(0.897)	(0.544)	(0.549)	(1.184)	(0.917)	(1.191)
Last policy EQUIDISTANCE*P2	1.019	0.046	1.258	0.540	−0.029	0.676
	(0.719)	(0.687)	(0.767)	(0.908)	(0.910)	(1.138)
Last policy EQUIDISTANCE*P3	0.704	0.819	2.286**	−0.073	0.221	1.537
	(0.946)	(0.800)	(0.898)	(1.220)	(1.082)	(1.418)
Last policy EQUIDISTANCE*P4	1.891**	0.847	2.115***	1.163	0.124	0.139
	(0.846)	(0.731)	(0.798)	(1.016)	(0.980)	(1.279)
Last policy EQUIDISTANCE*P5	2.979***	1.361*	2.800***	2.355*	0.502	0.746
	(1.138)	(0.766)	(0.824)	(1.407)	(1.033)	(1.316)
State fixed effects	No	No	No	Yes	Yes	Yes
Observations	293	480	467	293	480	467
Log Likelihood	-177.464	-309.510	-304.702	-144.511	-237.476	-166.686
Akaike Inf. Crit.	374.929	639.020	629.403	397.022	702.952	643.372

Note: *p<0.1; **p<0.05; ***p<0.01
Robust standard errors clustered by state are in parentheses.

Table 3: Logistic regression models with making a new policy that expresses a preference for equidistance as the binary response variable. Only state-years where a state makes a new policy are considered.

Figure 5 depicts the distribution of states using a box plot with those states marked as outliers.

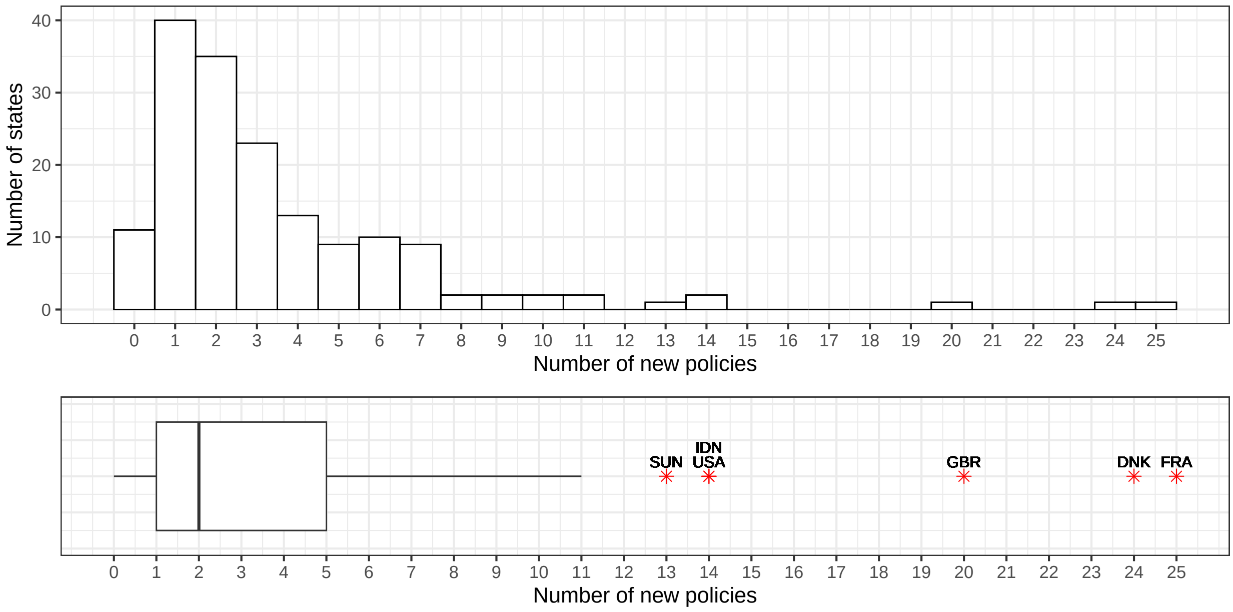

Figure 5: Distribution of state policies. Outliers indicated in red (from right to left): France (FRA), Denmark (DNK), the UK (GBR), Indonesia (IDN), the United States (USA), and the Soviet Union (SUN).

7 Alternative explanation: decolonization

We consider the possibility that the patterns we observe are in part due to newly independent states joining the system during decolonization and after the end of the Cold War. These new states may be more likely to adopt policies other than equidistance or different from those states that they once formed part of (”parent states”). Especially if the parent states were proponents of equidistance, the processes of decolonization and independence in the post-Cold War period may be driving *both* the reduction in equidistance and the increase in diversity in the periods we consider.

We identify the newly independent states in two steps. First, we consider those states that were former colonies. We use data from Teorell et al. (2023) that classifies countries according to their former colonies. From this list, we exclude those countries that gained their independence before 1945, such as the former Spanish and Portuguese colonies in Latin America. We also assign Vanuatu as a British colony, although it was ruled by a British-French condominium. Second, we consider states that gained their independence after the end of the Cold War, such as the Baltic states as well as the former Yugoslav republics. We complement this list with a few cases of independence that do not fit in these criteria (Eritrea, Singapore, Namibia, Nauru, etc.). We list the parent states and new states in 4 below.

Independence from	New states	n
United Kingdom	Antigua and Barbuda, Bahamas, Bahrain, Barbados, Belize, Brunei, Dominica Eswatini, Fiji, Gambia, Grenada, Guyana, Jamaica, Kenya, Kiribati, Kuwait, Lesotho, Malawi, Maldives, Malta, Mauritius, Qatar, Saint Kitts and Nevis, Saint Lucia, Saint Vincent and the Grenadines, Samoa, Seychelles, Sierra Leone, Solomon Islands, Tanzania, Tonga, Trinidad and Tobago, Tuvalu, Uganda, United Arab Emirates, Vanuatu	36
Portugal	Angola, Cabo Verde, Guinea-Bissau, Mozambique, Sao Tome and Principe	5
USSR	Estonia, Georgia, Latvia, Lithuania, Ukraine	5
United States	Marshall Islands, Micronesia, Palau	3
Yugoslavia	Bosnia and Herzegovina, Croatia, Slovenia	3
France	Algeria, Comoros, Djibouti	3
Australia	Nauru, Papua New Guinea	2
Pakistan	Bangladesh	1
Spain	Equatorial Guinea	1
Ethiopia	Eritrea	1
Indonesia	Timor-Leste	1
The Netherlands	Suriname	1
Malaysia	Singapore	1
South Africa	Namibia	1

Table 4: Newly independent states and the states from which they last separated.

Most of these countries became independent in the 1960s and 1970s (during which time global diversity became particularly high), with another wave in the 1990s. The distribution of independence years are depicted in Figure 6.

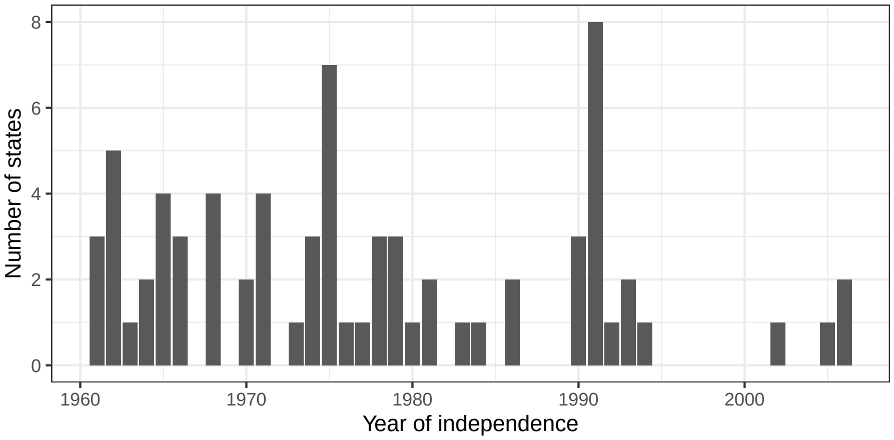

Figure 6: Distribution of states that became independent after 1960 according to their years of independence.

Focusing on the year of independence of each state, we can note that the most popular parent state policy was equidistance with 29 instances, followed by modified equidistance with 21 and nonequdisitance with 11 cases of new independence.

No policy	Spain (1), Pakistan (1)
EQ	United Kingdom (19), Portugal (5), Australia (1), France (1), Malaysia (1), United States (1), South Africa (1)
MODEQ	United Kingdom (17), Yugoslavia (3), Indonesia (1)
NONEQ	USSR (5), France (2) United States (2), Australia (1), The Netherlands (1)

Table 5: The policies of parent states at the time of the independence of new states. In parenthesis, we indicate how many newly independent states are concerned. Key: EQ - Strict or simplified equidistance, MODEQ - Modified equidistance, NONEQ - Nonequidistance.

Finally, let us consider what the newly independent states did upon independence. Do we observe any sort of reaction against parent states' policies, especially when the policy in question is equidistance, in a way that can drive diversity up? Table 6 presents what policies these newly independent states adopted during the first 10 years of their independence.

	Policy of newly independent state		
Policy of parent state at time of independence	Strict or simplified equidistance	Modified equidistance	Nonequidistance
Strict or simplified equidistance	15 (5)	1 (0)	0 (0)
Modified equidistance	16 (16)	2 (2)	1 (1)
Nonequidistance	4 (4)	5 (5)	3 (3)

Table 6: The policies adopted by newly independent states within the first ten years of their independence. In parenthesis, we exclude those states that became independent before the first court decision, 1969.

Instead of reacting against their parent states' policies, newly independent states appear to be particulalry attracted to equidistance. This is especially the case if the parent state's policy was also equidistance; but note as well how those states that could "inherit" modified equidistnace from their parent states also overwhelmingly chose equidistance. Only when the parent state's policy was nonequidistance do we see a more even distribution of independent state choices. This means that, rather than being responsible for driving down the popularity of equidistance, newly independent states actually kept equidistance popular.

The following regressions attest to this.

	Dependent variable:		
	eq		
	(1)	(2)	(3)
Newly independent	0.724***	14.410***	14.244***
	(0.196)	(0.473)	(0.560)
PERIOD: Between 1969 and 1985 [P2]		−1.647***	−1.742***
		(0.324)	(0.450)
PERIOD: Between 1985 and 1993 [P3]		−1.822***	−1.976***
		(0.400)	(0.534)
PERIOD: Between 1993 and 1907 [P4]		−1.032***	−1.650***
		(0.359)	(0.512)
PERIOD: After 2007 [P5]		−1.265***	−2.148***
		(0.391)	(0.613)
Last policy EQUIDISTANCE			−0.568
			(0.595)
Newly independent*P2		−12.399***	−11.831***
		(0.654)	(0.863)
Newly independent*P3		−14.149***	−14.284***
		(0.719)	(0.907)
Newly independent*P4		−14.088***	−13.548***
		(0.615)	(0.779)
Newly independent*P5		−13.859***	−14.024***
		(0.626)	(0.891)
Newly independent*Last policy EQUIDISTANCE			0.545
			(0.899)
P2*Last policy EQUIDISTANCE			0.349
			(0.690)
P3*Last policy EQUIDISTANCE			0.529
			(0.856)
P4*Last policy EQUIDISTANCE			1.550**
			(0.757)
P5*Last policy EQUIDISTANCE			1.774**
			(0.837)
Newly independent*P2*Last policy EQUIDISTANCE			−1.220
			(1.335)
Newly independent*P3*Last policy EQUIDISTANCE			0.187
			(1.446)
Newly independent*P4*Last policy EQUIDISTANCE			−1.356
			(1.207)
Constant	−0.089	1.156***	1.322***
	(0.097)	(0.281)	(0.405)
Observations	582	582	572
Log Likelihood	-395.654	-369.205	-354.039
Akaike Inf. Crit.	795.308	758.411	746.079
Note:			*p<0.1; **p<0.05; ***p<0.01

Table 7: Regression results for simple models including newly independent states interaction with periods and equidistance as last policy.

In Figure 7 and 8, we observe that newly independent states are in fact more likely to adopt equidistance compared to other states. Surprisingly, this is especially the case in the second period, right after equidistance was shunned by the ICJ in its 1969 *North Sea* decision.

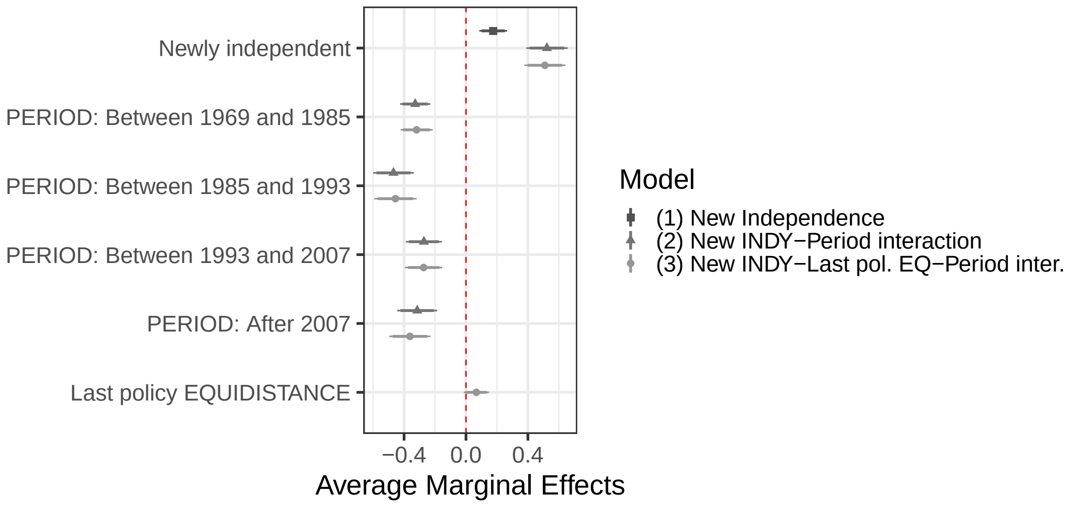

Figure 7: Average marginal effects for models testing the association between newly independent states and the probability of equidistance.

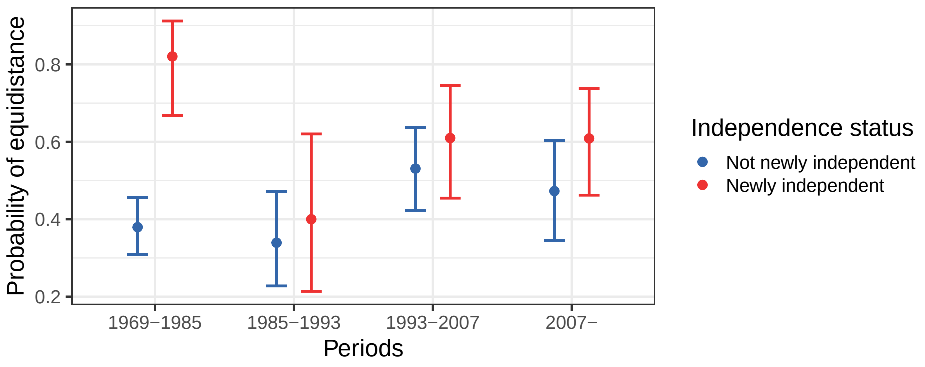

Figure 8: Predictions based on the interaction between new independence and equidistance based on Model 2, ignoring the first period where no newly independent state did something other than equidistance.

What all this shows is that decolonization is not a plausible explanation for increasing diversity. On the contrary, new states make policies that serve to decrease diversity or at least to keep it stable. Newly independent states tend to adopt equidistance at the expense of other methods, for many possible reasons. These states may have geographies and coastal configurations that make equidistance an appropriate method, if not the most obvious one. This may be the case of small island states in the Pacific and the Caribbean that become independent and have common boundaries to delimit with states of similar size and geographies. Between these states, it may be hard to justify opting for anything other than equidistance. Another reason may be that even when states want to deviate from equidistance, they may not have the resources to engage in negotiations to agree on a different delimitation method. By comparison, drawing an equidistant boundary is a relatively simple and technical exercise.

In our case, then, the increase in the number of states could not have created something akin to the defocalizing effect we discuss, as these states reinforced, rather than abandoned the focal rule. In other situations where the simple solution that newly independent states adopt is not the plausible focal rule, their preference for the simple solution may contribute to diversity; this is not the case in ours.

8 Selection models

We fit two selection models. In the selection step, we include variables that can plausibly affect selection but not the outcome, such as GDP per capita (logged), number of dyadic boundary relations, and proportion of fully delimited boundaries. We also include variables that may affect both, such as the existence of a maritime boundary dispute or a related territorial dispute. As the correlation between the errors of the two parts of the selection equation (rho) is not statistically different from zero, the selection and outcome equations can be estimated separately.

	(1)		(2)	
	Selection	Outcome	Selection	Outcome
Maritime boundary dispute (lagged)	0.247***	−0.556***	0.241***	−0.487***
	(0.058)	(0.182)	(0.062)	(0.186)
Related territorial dispute (lagged)	−0.110*	0.113	−0.088	0.114
	(0.061)	(0.143)	(0.061)	(0.139)
GDP per capita (logged)	0.058**		0.423*	
	(0.024)		(0.024)	
Number of dyadic boundary relations (lagged)	0.043***		0.040***	
	(0.006)		(0.005)	
Proportion of fully delimited boundaries (lagged)	−0.545***		−0.519***	
	(0.102)		(0.108)	
Time since last new policy	−0.057***		−0.052***	
	(0.014)		(0.014)	
Time since last new policy squared	0.002**		0.002**	
	(0.001)		(0.001)	
Time since last new policy cubed	−0.00002		0.00001	
	(0.00001)		(0.00001)	
Ratified UNCLOS			−0.081	−0.081
			(0.052)	(0.232)
Democracy			0.186***	−0.118
			(0.070)	(0.180)
LEGAL SYSTEM: Common Law			−0.067	0.255
			(0.068)	(0.170)
LEGAL SYSTEM: Islamic			−0.100	−0.200
			(0.085)	(0.230)
LEGAL SYSTEM: Mixed			−0.080	−0.008
			(0.117)	(0.261)
Last policy EQUIDISTANCE		0.235**		−0.044
		(0.118)		(0.453)
PERIOD: Between 1969 and 1985 [P2]		−0.725**		−0.747**
		(0.289)		(0.354)
PERIOD: Between 1985 and 1993 [P3]		−0.981***		−1.264***
		(0.331)		(0.424)
PERIOD: Between 1993 and 2007 [P4]		−0.462*		−0.600
		(0.279)		(0.389)
PERIOD: After 2007 [P5]		−0.604**		−0.983**
		(0.294)		(0.447)
Last policy EQUIDISTANCE*P2				−0.095
				(0.475)
Last policy EQUIDISTANCE*P3				0.577
				(0.584)
Last policy EQUIDISTANCE*P4				0.423
				(0.518)
Last policy EQUIDISTANCE*P5				0.717
				(0.534)
Constant	−1.766***	0.715	−1.714***	0.760
	(0.159)	(0.500)	(0.172)	(0.670)
Observations	6,717	491	6,717	491
Log pseudolikelihood	-1,934.847		-1,919.121	
ρ	0.032 (0.236)		0.087 (0.300)	
Note:				*p<0.1; **p<0.05; ***p<0.01
				Robust standard errors clustered by state are in parentheses.

Table 8: Probit models with sample selection. For each model, the selection equation models the probability of making a policy, and the outcome equation is that of expressing a preference for equidistance with that policy.

Figure 9 present predictive margins for the probability of choosing equidistance given that the state makes a new policy, based on the second model. The results are consistent with our main findings about the changes in the popularity of equidistance across the periods.

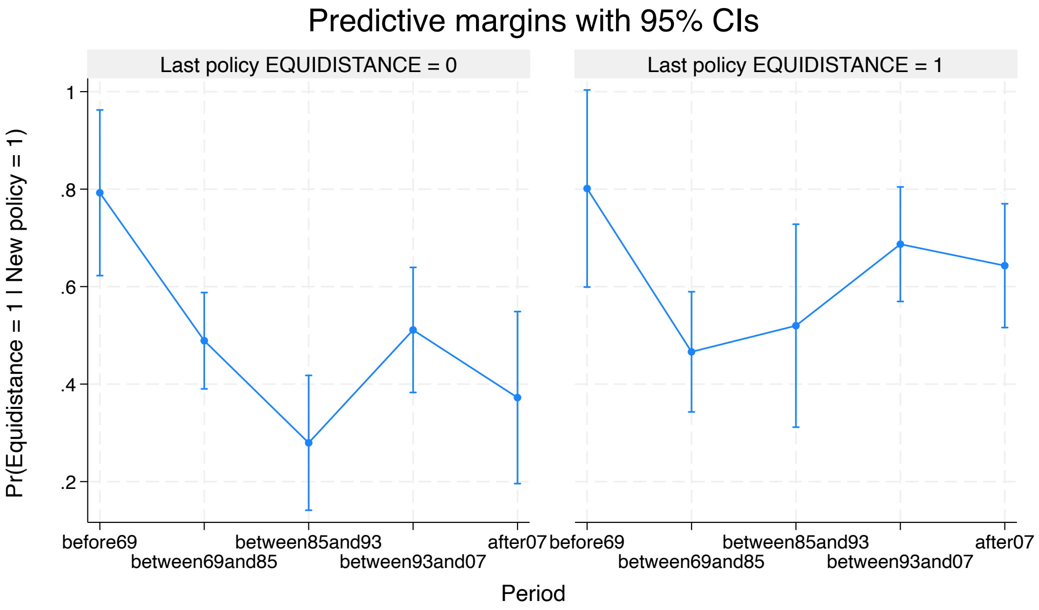

Figure 9: Average predictions for equidistance conditional on making a policy, estimated using the selection model with interaction between period and a dummy indicating whether the state's last policy was equidistance.

9 Time since decision and types of incongruence/inconsistency

Table 9 presents the output of an OLS regression that takes diversity as its dependent variables and includes and interaction between different combinations of levels of incongruence and types of inconsistency (proxied by our four periods) and the time since the adoption of the four key ICJ decisions.

	Dependent variable:
	Diversity score (Inverse Simpson index)
Moderate incongruence & Incremental inconsistency [P3]	-1.054^{***}
	(0.134)
Low incongruence & Incremental inconsistency [P4]	-0.050^{*}
	(0.026)
Low incongruence & Random inconsistency [P5]	0.200^{***}
	(0.020)
Years since decision [Y]	0.825^{***}
	(0.107)
Years since decision^2 [Y^2]	-1.323^{***}
	(0.106)
Years since decision^3 [Y^2]	0.436^{***}
	(0.103)
P3*Y	-13.375^{***}
	(1.441)
P4*Y	-0.544^{***}
	(0.133)
P5*Y	-0.060
	(0.139)
P3*Y^2	-8.143^{***}
	(1.175)
P4*Y^2	1.276^{***}
	(0.156)
P5*Y^2	1.205^{***}
	(0.140)
P3*Y^3	-3.291^{***}
	(0.494)
P4*Y^3	-0.528^{***}
	(0.170)
P5*Y^3	-0.490^{***}
	(0.129)
Constant	2.309^{***}
	(0.018)
Observations	50
R^2	0.937
Adjusted R^2	0.909
Residual Std. Error	0.052 (df = 34)
F Statistic	33.625^{***} (df = 15; 34)
Note:	*p<0.1; **p<0.05; ***p<0.01

Table 9: The evolution of diversity after key decisions representing different levels of incongruence and types of inconsistency. The reference category is P2: High incongruence.

10 Multinomial logistic regressions

The regression output in Table 10 captures three multinomial logistic regression models that have the choice of policy as their dependent variable.

	Dependent variable:					
	Modified equidistance	Nonequidistance	Modified equidistance	Nonequidistance	Modified equidistance	Nonequidistance
	(1)	(2)	(3)	(4)	(5)	(6)
Between 1969 and 1985 [P2]	−0.093	0.122	−0.144	0.067	−0.123	−0.011
	(0.209)	(0.212)	(0.217)	(0.223)	(0.169)	(0.167)
Between 1985 and 1993 [P3]	0.089	0.398	0.081	0.349	0.040	0.221
	(0.281)	(0.275)	(0.283)	(0.287)	(0.226)	(0.219)
Between 1993 and 2007 [P4]	−0.224	-0.956^{***}	−0.034	-1.008^{***}	−0.052	-0.709^{***}
	(0.212)	(0.274)	(0.231)	(0.291)	(0.181)	(0.233)
After 2007 [P5]	-0.395^{*}	-0.590^{**}	−0.165	−0.338	−0.259	−0.130
	(0.221)	(0.250)	(0.234)	(0.267)	(0.272)	(0.225)
Last policy EQ			-0.601^{***}	-0.782^{***}	-0.480^{***}	-0.632^{***}
			(0.178)	(0.200)	(0.151)	(0.159)
Last policy MODEQ			0.250	-0.506^{**}	0.199	-0.387^{*}
			(0.207)	(0.249)	(0.174)	(0.208)
Last policy NONEQ			0.089	0.358	−0.112	0.389^{**}
			(0.236)	(0.235)	(0.238)	(0.198)
Marit. bound. dispute (lagged) >0	0.602^{**}	1.025^{***}	0.507^{*}	0.845^{***}	0.537^{*}	0.910^{***}
	(0.261)	(0.298)	(0.278)	(0.313)	(0.274)	(0.309)
Region: Europe+post-Soviet			0.0003	0.910^{**}		
			(0.384)	(0.438)		
Region: Med. and ME			−0.246	0.114		
			(0.387)	(0.475)		
Region: Sub-Saharan Africa			-1.324^{**}	0.320		
			(0.617)	(0.564)		
Region: The Americas			−0.163	0.405		
			(0.383)	(0.447)		
Last policy EQ*P2					0.111	0.311
					(0.289)	(0.286)
Last policy MODEQ*P2					0.056	−0.065
					(0.304)	(0.347)
Last policy NONEQ*P2					−0.289	−0.257
					(0.365)	(0.311)
Last policy EQ*P3					0.201	0.111
					(0.373)	(0.374)
Last policy MODEQ*P3					−0.165	−0.120
					(0.394)	(0.428)
Last policy NONEQ*P3					0.004	0.230
					(0.497)	(0.423)
Last policy EQ*P4					-0.560^{*}	−0.269
					(0.290)	(0.359)
Last policy MODEQ*P4					−0.322	−0.562
					(0.355)	(0.522)
Last policy NONEQ*P4					0.831^{**}	0.121
					(0.385)	(0.433)
Last policy EQ*P5					−0.232	-0.785^{**}
					(0.343)	(0.342)
Last policy MODEQ*P5					0.630	0.360
					(0.417)	(0.441)
Last policy NONEQ*P5					−0.658	0.295
					(0.723)	(0.495)
Constant	-0.623^{***}	-1.026^{***}	−0.261	-0.930^{***}	-0.394^{***}	-0.630^{***}
	(0.165)	(0.199)	(0.186)	(0.244)	(0.144)	(0.161)
Akaike Inf. Crit.	754.706	754.706	744.093	744.093	752.958	752.958
Note:						*p<0.1; **p<0.05; ***p<0.01

Table 10: Multinomial logistic regressions, with equidistance as the baseline category. The output of first model is marked (1) and (2), the second as (3) and (4), and the third as (5) and (6).

11 Counterfactual contribution to diversity

Table 11 presents regression output for an OLS model including the counterfactual contribution to diversity of a policy as the dependent variable.

	Dependent variable:
	Counterfactual contribution to diversity
Time [T1]	-0.224^{***}
	(0.053)
Time squared [T2]	0.157^{**}
	(0.063)
Time cubed [T3]	-0.075
	(0.055)
Equidistance	-0.037^{***}
	(0.002)
Equidistance*T1	0.389^{***}
	(0.058)
Equidistance*T2	-0.133^{*}
	(0.068)
Equidistance*T3	0.092
	(0.063)
Constant	0.021^{***}
	(0.002)
Observations	585
R^2	0.487
Adjusted R^2	0.480
Residual Std. Error	0.020 (df = 577)
F Statistic	78.159^{***} (df = 7; 577)
Note:	*p<0.1; **p<0.05; ***p<0.01

Table 11: Counterfactual contribution to diversity of having equdisitance as a new policy over time.

References

Bjørnskov, C. and M. Rode (2020). Regime types and regime change: A new dataset on democracy, coups, and political institutions. *The Review of International Organizations 15*(2), 531–551.

Hadenius, A. and J. Teorell (2007). Pathways from Authoritarianism. *Journal of Democracy 18*(1), 143–156.

Powell, E. J. and S. M. Mitchell (2007). The International Court of Justice and the World's Three Legal Systems. *The Journal of Politics 69*(2), 397–415. Publisher: [The University of Chicago Press, Southern Political Science Association].

Teorell, J., A. Sundström, S. Holmberg, B. Rothstein, N. Alvarado Pachon, C. M. Dalli, and Y. Meijers (2023). The Quality of Governance Standard Dataset.

Yüksel, A. U. (2019). Bargaining over maritime boundaries in times of legal uncertainty. Place: Geneva Publisher: Graduate Institute of International and Development Studies.
