# Supplementary material for: The defocalizing effect of international courts: Evidence from maritime delimitation practices
Source: Rev Int Organ. 2024 Jun 29;20(4):825–61. doi: 10.1007/s11558-024-09545-4 (PMC12727788; doi:10.1007/s11558-024-09545-4)
Supplement: Supplementary file 2 — Supplementary file2 (ZIP 112225 kb) [file 11558_2024_9545_MOESM2_ESM.zip › The Defocalizing Effect - Replication/2 Analysis/2.1 R/Figures/Not Reported AMEs.pdf]

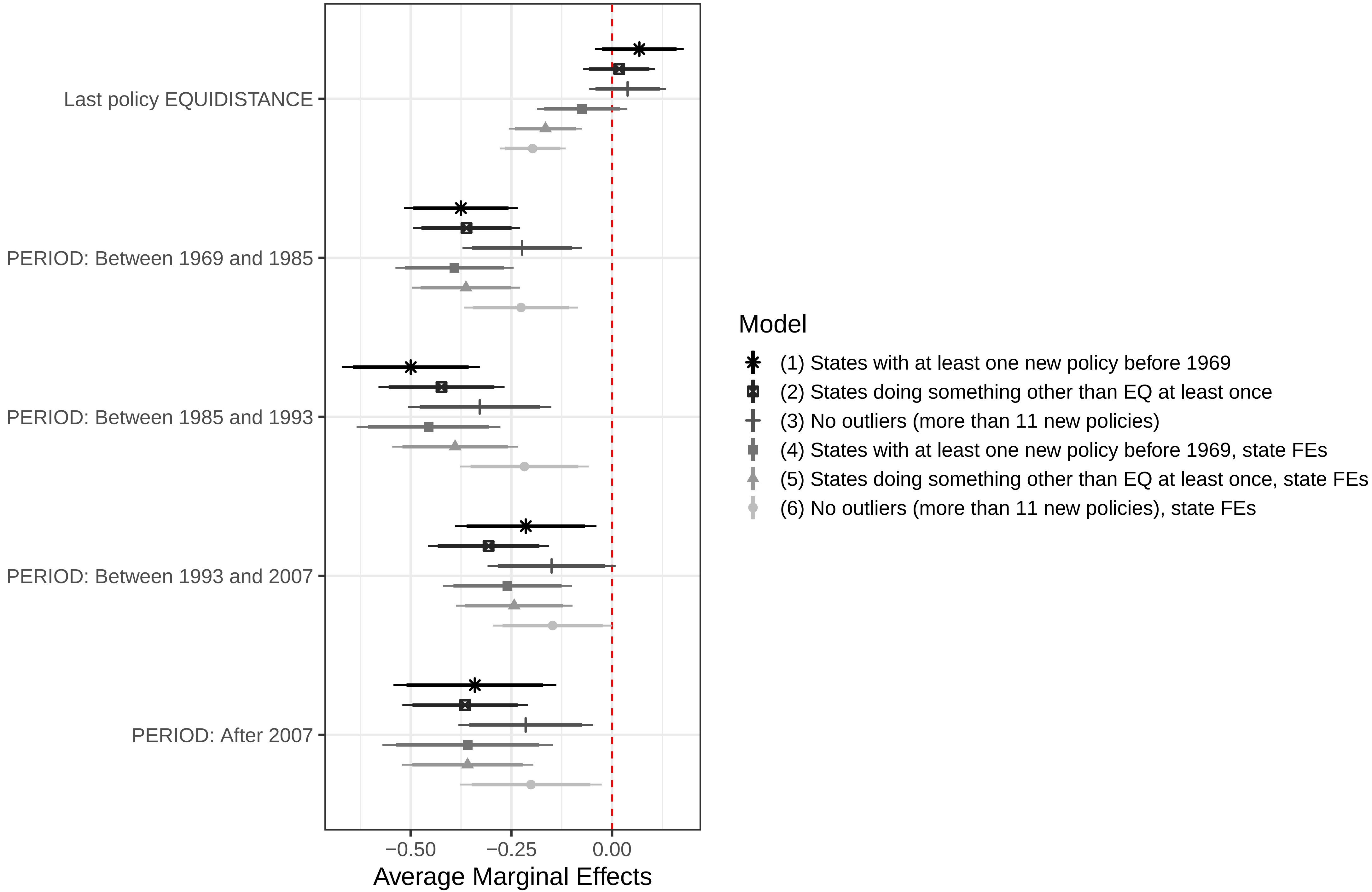
Last policy EQUIDISTANCE
PERIOD: Between 1969 and 1985
PERIOD: Between 1985 and 1993
PERIOD: Between 1993 and 2007
PERIOD: After 2007
−0.50
−0.25
0.00
Average Marginal Effects
Model
(1) States with at least one new policy before 1969
(2) States doing something other than EQ at least once
(3) No outliers (more than 11 new policies)
(4) States with at least one new policy before 1969, state FEs
(5) States doing something other than EQ at least once, state FEs
(6) No outliers (more than 11 new policies), state FEs
