# Supplementary material for: The defocalizing effect of international courts: Evidence from maritime delimitation practices
Source: Rev Int Organ. 2024 Jun 29;20(4):825–61. doi: 10.1007/s11558-024-09545-4 (PMC12727788; doi:10.1007/s11558-024-09545-4)
Supplement: Supplementary file 2 — Supplementary file2 (ZIP 112225 kb) [file 11558_2024_9545_MOESM2_ESM.zip › The Defocalizing Effect - Replication/2 Analysis/2.1 R/Figures/Appendix Figure 2.pdf]

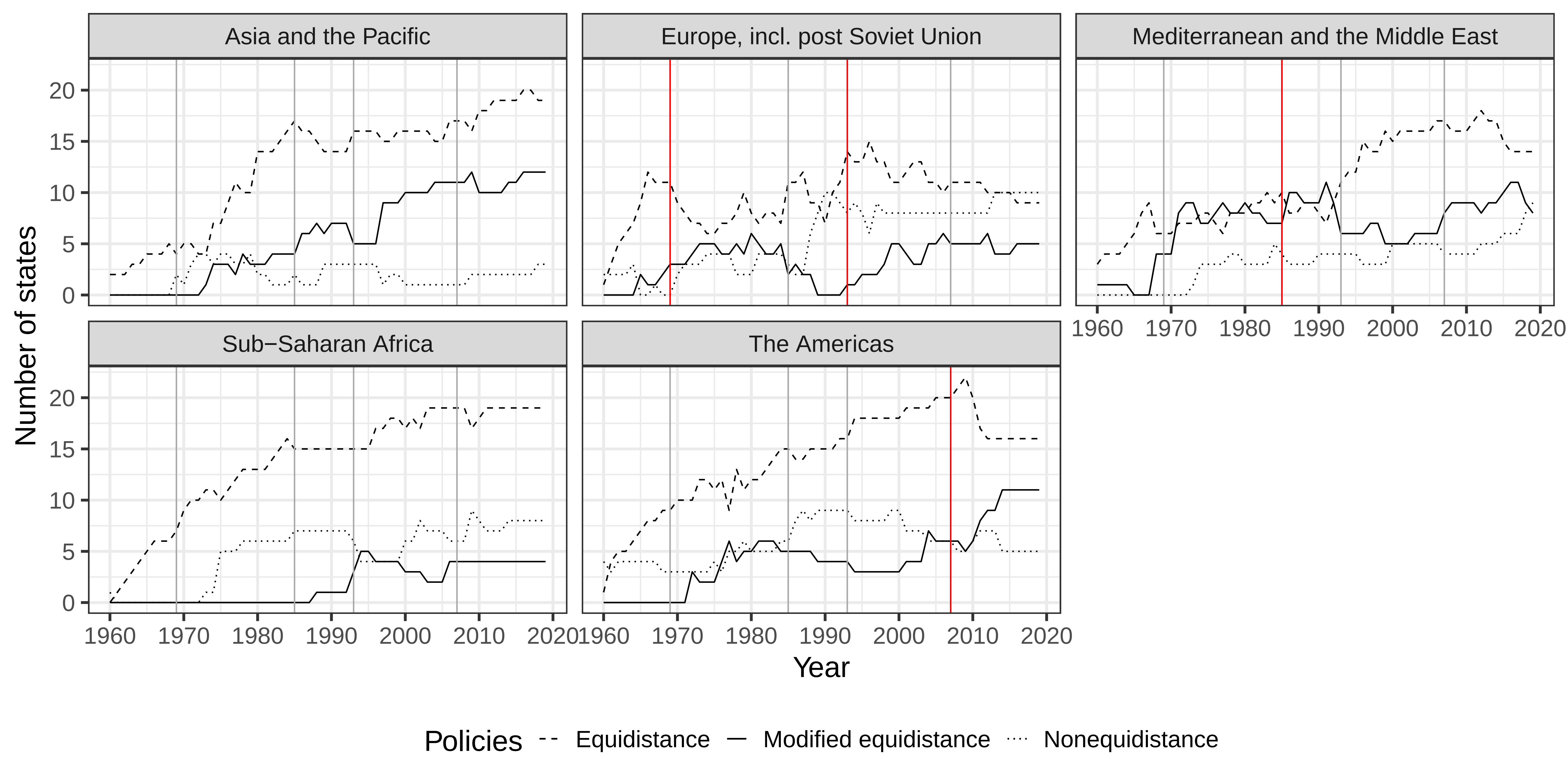

Asia and the Pacific
Europe, incl. post Soviet Union
Mediterranean and the Middle East
Sub−Saharan Africa
The Americas
Number of states
0
5
10
15
20
1960
1970
1980
1990
2000
2010
2020
Year
Policies
Equidistance
Modified equidistance
Nonequidistance
