# Supplementary material for: The defocalizing effect of international courts: Evidence from maritime delimitation practices
Source: Rev Int Organ. 2024 Jun 29;20(4):825–61. doi: 10.1007/s11558-024-09545-4 (PMC12727788; doi:10.1007/s11558-024-09545-4)
Supplement: Supplementary file 2 — Supplementary file2 (ZIP 112225 kb) [file 11558_2024_9545_MOESM2_ESM.zip › The Defocalizing Effect - Replication/2 Analysis/2.2 STATA/heckprobit.doc]

	(1)	(2)	(3)	
VARIABLES	eq	new_policy	/	
				
1.any_amdisp_lag	-0.556***	0.247***		
	(0.182)	(0.0584)		
1.any_atdisp_lag	0.113	-0.110*		
	(0.143)	(0.0605)		
wdi_gdpcapcur_log		0.0581**		
		(0.0235)		
nrelc_aggr_lag		0.0429***		
		(0.00581)		
prop_nF0_nrelc_aggr_lag		-0.545***		
		(0.102)		
policy_cntr		-0.0573***		
		(0.0141)		
policy_cntr_sq		0.00174**		
		(0.000687)		
policy_cntr_cubed		-1.60e-05*		
		(9.25e-06)		
2.at_eq_lag	0.235**			
	(0.118)			
2.period5	-0.725**			
	(0.289)			
3.period5	-0.981***			
	(0.331)			
4.period5	-0.462*			
	(0.279)			
5.period5	-0.604**			
	(0.294)			
athrho			0.0316	
			(0.236)	
Constant	0.715	-1.766***		
	(0.500)	(0.159)		
				
Observations	6,717	6,717	6,717	
Robust standard errors in parentheses
*** p<0.01, ** p<0.05, * p<0.1
