# Supplementary material for: The defocalizing effect of international courts: Evidence from maritime delimitation practices
Source: Rev Int Organ. 2024 Jun 29;20(4):825–61. doi: 10.1007/s11558-024-09545-4 (PMC12727788; doi:10.1007/s11558-024-09545-4)
Supplement: Supplementary file 2 — Supplementary file2 (ZIP 112225 kb) [file 11558_2024_9545_MOESM2_ESM.zip › The Defocalizing Effect - Replication/2 Analysis/2.1 R/Figures/Main Figure 8.pdf]

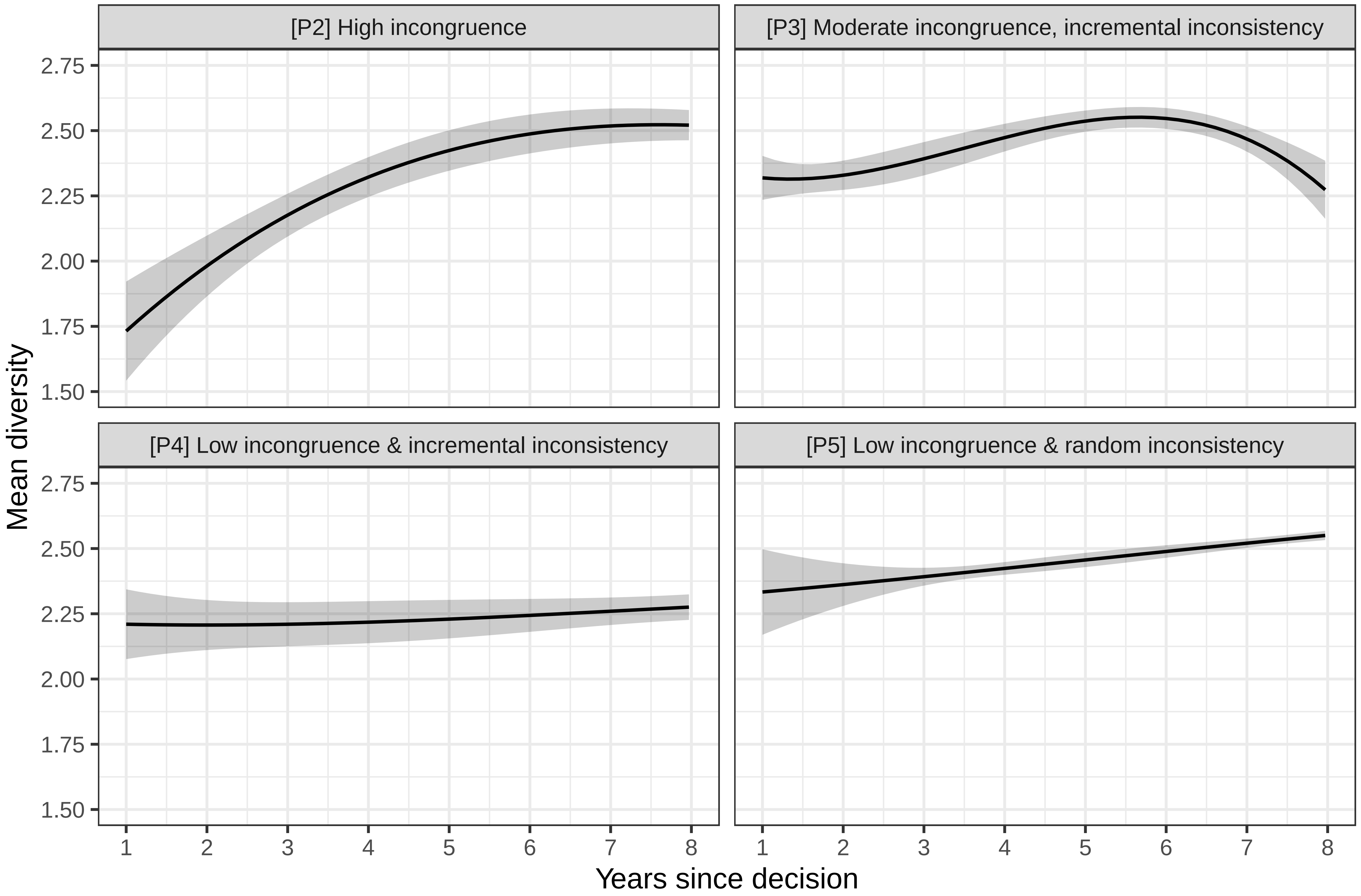
[P2] High incongruence
[P3] Moderate incongruence, incremental inconsistency
[P4] Low incongruence & incremental inconsistency
[P5] Low incongruence & random inconsistency
Mean diversity
2.75
2.50
2.25
2.00
1.75
1.50
1 2 3 4 5 6 7 8
Years since decision
