# Supplementary material for: The defocalizing effect of international courts: Evidence from maritime delimitation practices
Source: Rev Int Organ. 2024 Jun 29;20(4):825–61. doi: 10.1007/s11558-024-09545-4 (PMC12727788; doi:10.1007/s11558-024-09545-4)
Supplement: Supplementary file 2 — Supplementary file2 (ZIP 112225 kb) [file 11558_2024_9545_MOESM2_ESM.zip › The Defocalizing Effect - Replication/2 Analysis/2.2 STATA/heckprobit2.doc]

	(1)	(2)	(3)	
VARIABLES	eq	new_policy	/	
				
2.at_eq_lag	-0.0442			
	(0.453)			
2.period5	-0.747**			
	(0.354)			
3.period5	-1.264***			
	(0.424)			
4.period5	-0.600			
	(0.389)			
5.period5	-0.983**			
	(0.447)			
1b.at_eq_lag#1b.period5	0			
	(0)			
1b.at_eq_lag#2o.period5	0			
	(0)			
1b.at_eq_lag#3o.period5	0			
	(0)			
1b.at_eq_lag#4o.period5	0			
	(0)			
1b.at_eq_lag#5o.period5	0			
	(0)			
2o.at_eq_lag#1b.period5	0			
	(0)			
2.at_eq_lag#2.period5	-0.0951			
	(0.475)			
2.at_eq_lag#3.period5	0.577			
	(0.584)			
2.at_eq_lag#4.period5	0.423			
	(0.518)			
2.at_eq_lag#5.period5	0.717			
	(0.534)			
1.any_amdisp_lag	-0.487***	0.241***		
	(0.186)	(0.0621)		
1.any_atdisp_lag	0.114	-0.0876		
	(0.139)	(0.0607)		
unclos_filled	-0.0807	-0.0805		
	(0.232)	(0.0523)		
br_dem	-0.118	0.186***		
	(0.180)	(0.0698)		
2.legal_system	0.255	-0.0669		
	(0.170)	(0.0683)		
3.legal_system	-0.200	-0.100		
	(0.230)	(0.0853)		
4.legal_system	-0.00785	-0.0801		
	(0.261)	(0.117)		
wdi_gdpcapcur_log		0.0423*		
		(0.0244)		
nrelc_aggr_lag		0.0403***		
		(0.00545)		
prop_nF0_nrelc_aggr_lag		-0.519***		
		(0.108)		
policy_cntr		-0.0515***		
		(0.0139)		
policy_cntr_sq		0.00158**		
		(0.000683)		
policy_cntr_cubed		-1.44e-05		
		(9.23e-06)		
athrho			0.0868	
			(0.300)	
Constant	0.760	-1.714***		
	(0.670)	(0.172)		
				
Observations	6,717	6,717	6,717	
Robust standard errors in parentheses
*** p<0.01, ** p<0.05, * p<0.1
