# Supplementary material for: The defocalizing effect of international courts: Evidence from maritime delimitation practices
Source: Rev Int Organ. 2024 Jun 29;20(4):825–61. doi: 10.1007/s11558-024-09545-4 (PMC12727788; doi:10.1007/s11558-024-09545-4)
Supplement: Supplementary file 2 — Supplementary file2 (ZIP 112225 kb) [file 11558_2024_9545_MOESM2_ESM.zip › The Defocalizing Effect - Replication/2 Analysis/2.1 R/Figures/Appendix Figure 7.pdf]

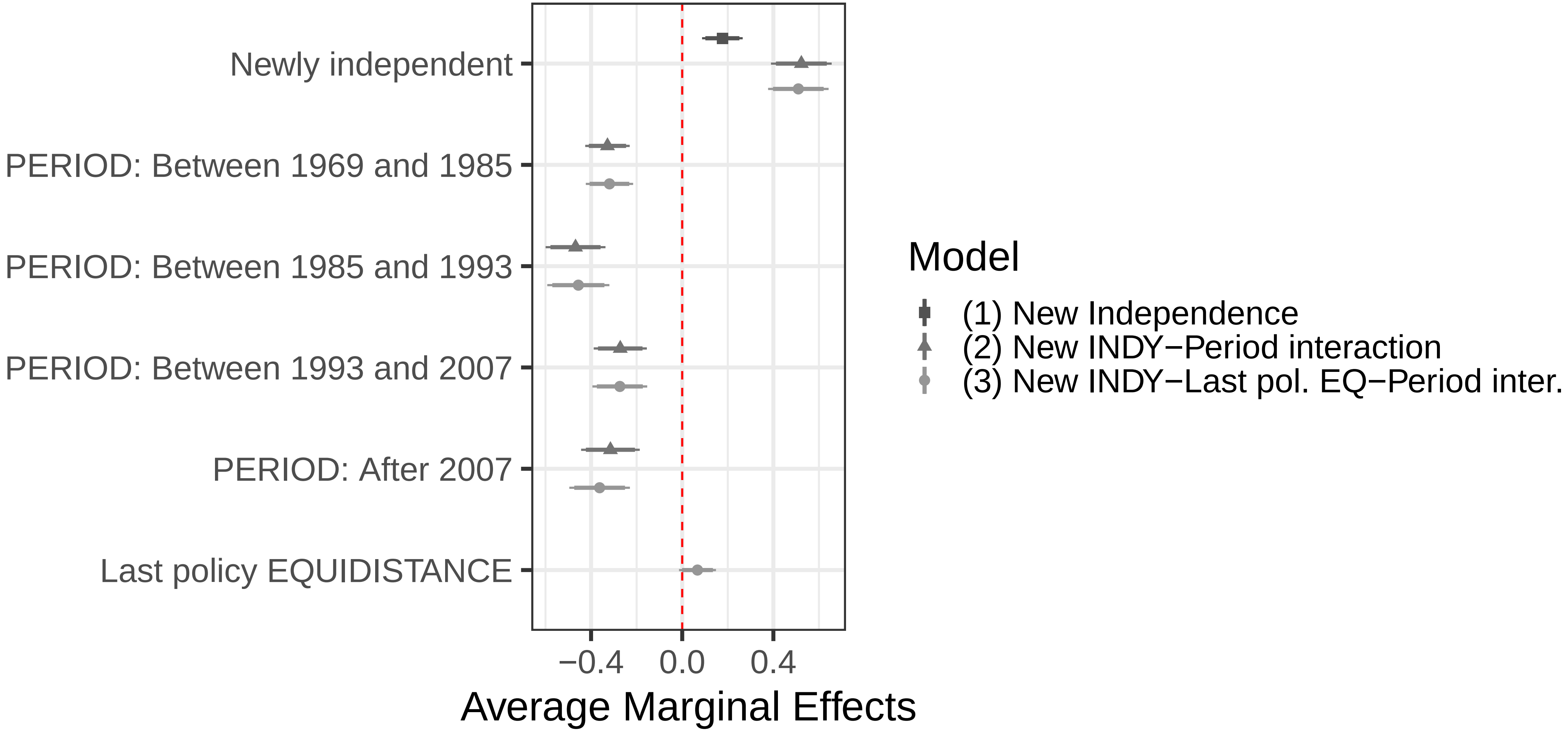

Newly independent
PERIOD: Between 1969 and 1985
PERIOD: Between 1985 and 1993
PERIOD: Between 1993 and 2007
PERIOD: After 2007
Last policy EQUIDISTANCE
−0.4
0.0
0.4
Average Marginal Effects
Model
(1) New Independence
(2) New INDY−Period interaction
(3) New INDY−Last pol. EQ−Period inter.
