# Supplementary figures and images for: The defocalizing effect of international courts: Evidence from maritime delimitation practices
Source: Rev Int Organ. 2024 Jun 29;20(4):825–61. doi: 10.1007/s11558-024-09545-4 (PMC12727788; doi:10.1007/s11558-024-09545-4)

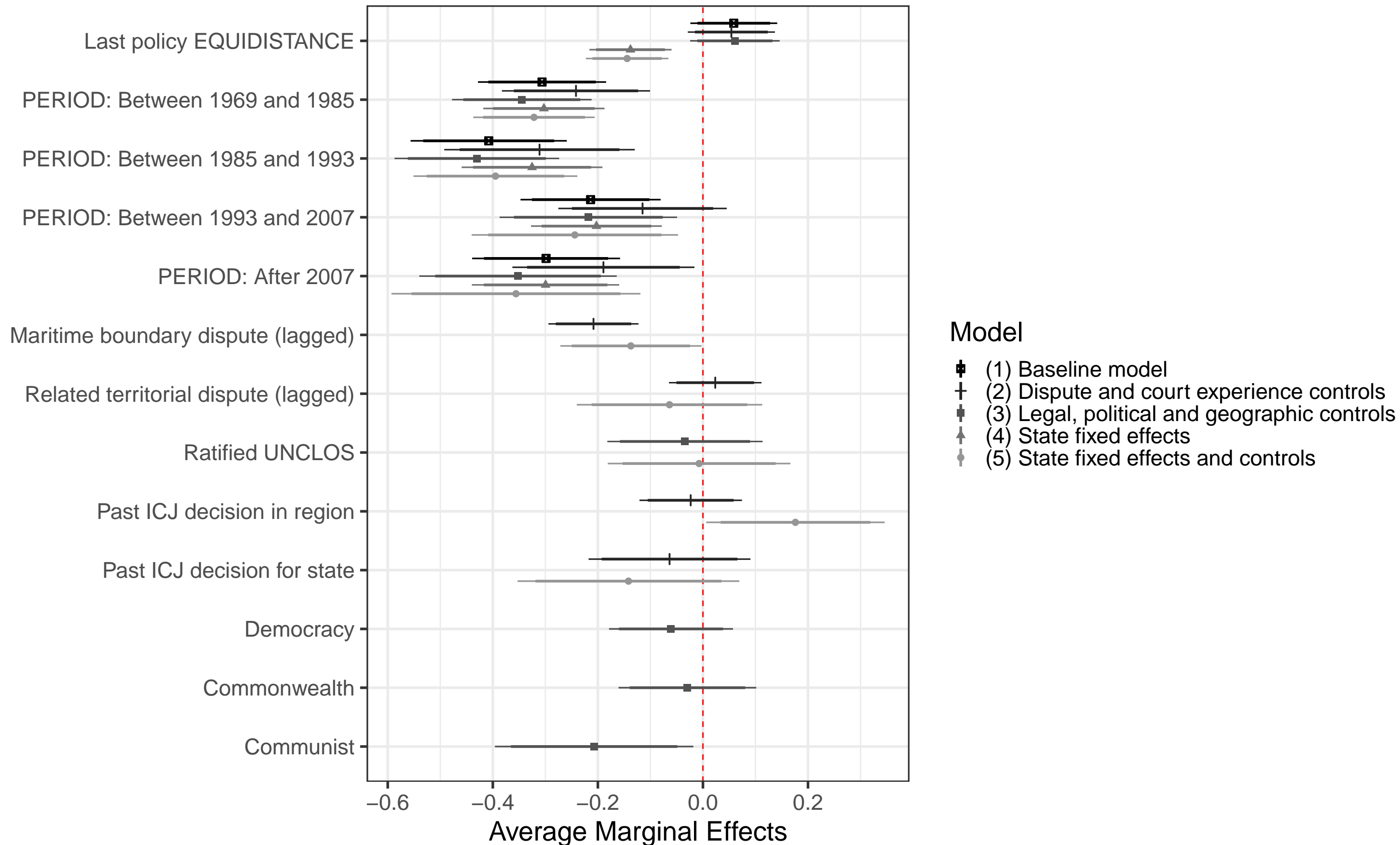

Supplement: Supplementary file 2 — Supplementary file2 (ZIP 112225 kb) [file 11558_2024_9545_MOESM2_ESM.zip › The Defocalizing Effect - Replication/2 Analysis/2.1 R/Figures/Main Figure 6.pdf]

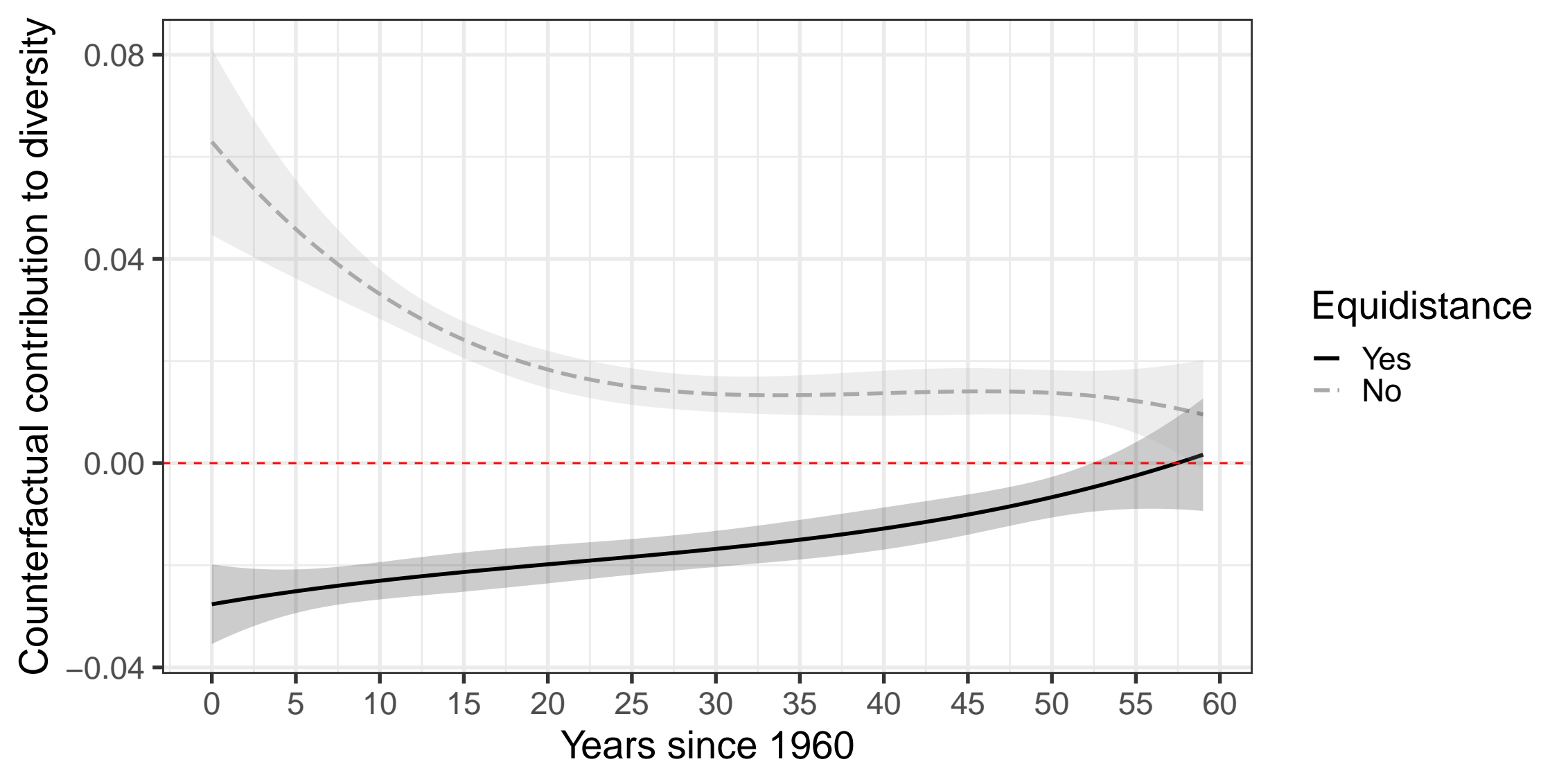

Supplement: Supplementary file 2 — Supplementary file2 (ZIP 112225 kb) [file 11558_2024_9545_MOESM2_ESM.zip › The Defocalizing Effect - Replication/2 Analysis/2.1 R/Figures/Main Figure 9.pdf]

Policies - · Equidistance — Modified equidistance ··· Nonequidistance

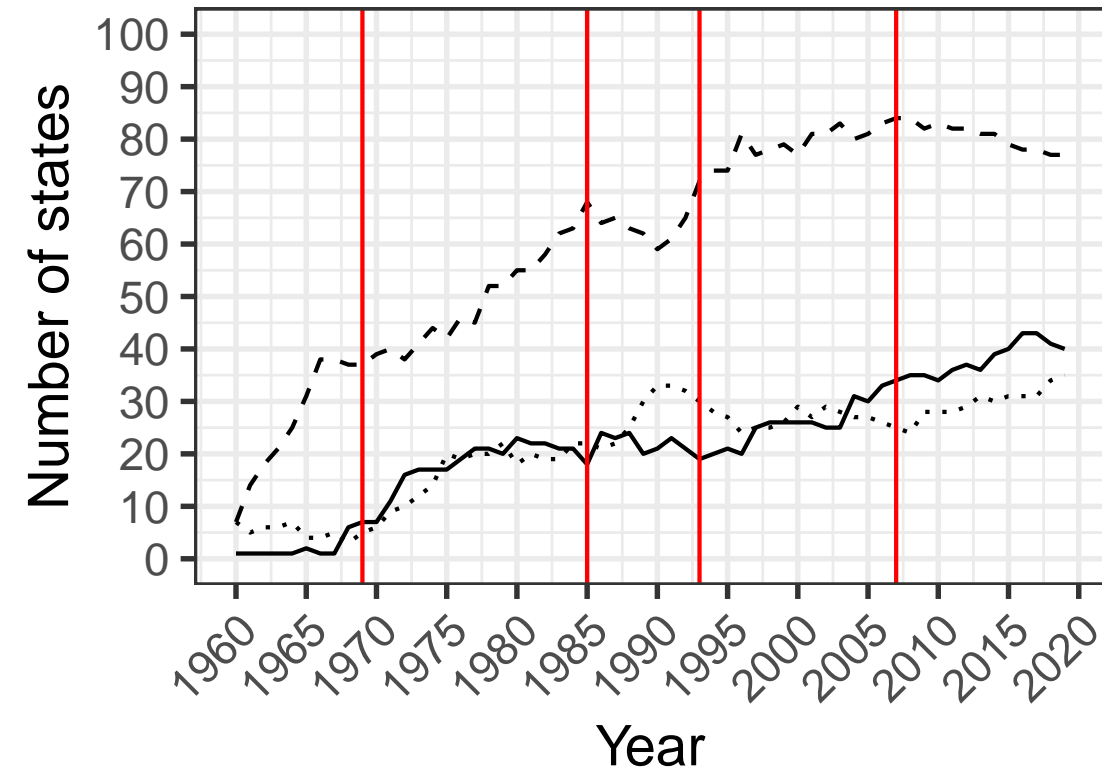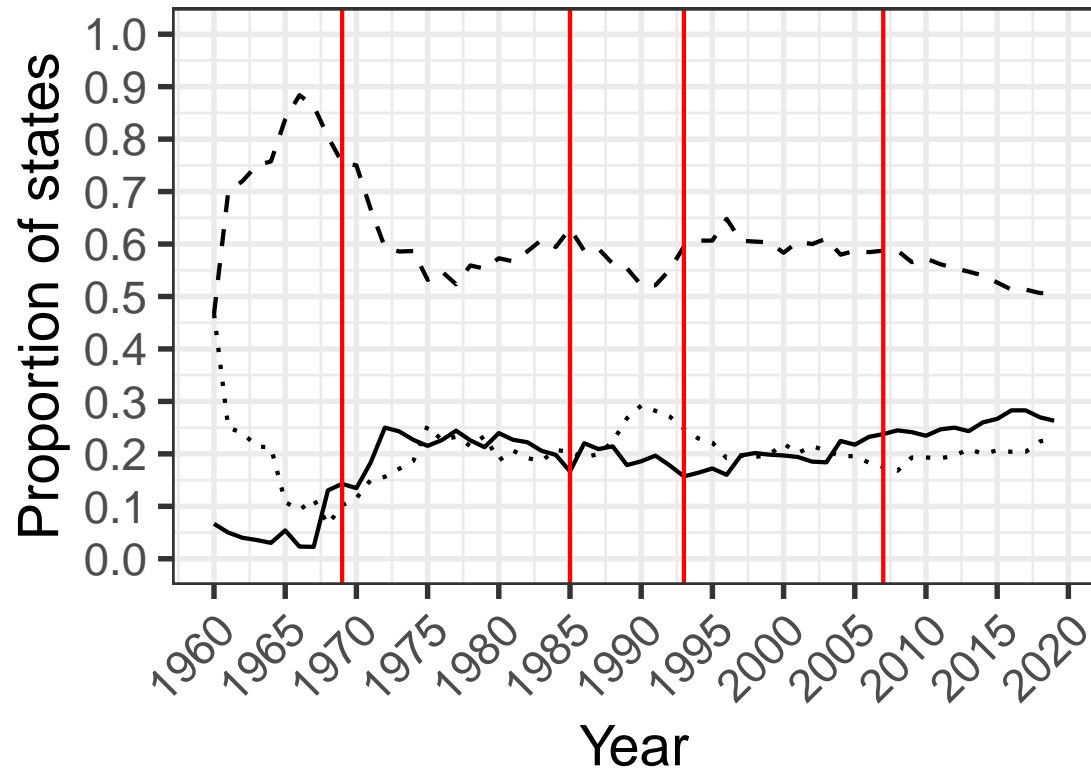

Supplement: Supplementary file 2 — Supplementary file2 (ZIP 112225 kb) [file 11558_2024_9545_MOESM2_ESM.zip › The Defocalizing Effect - Replication/2 Analysis/2.1 R/Figures/Main Figure 4.pdf]

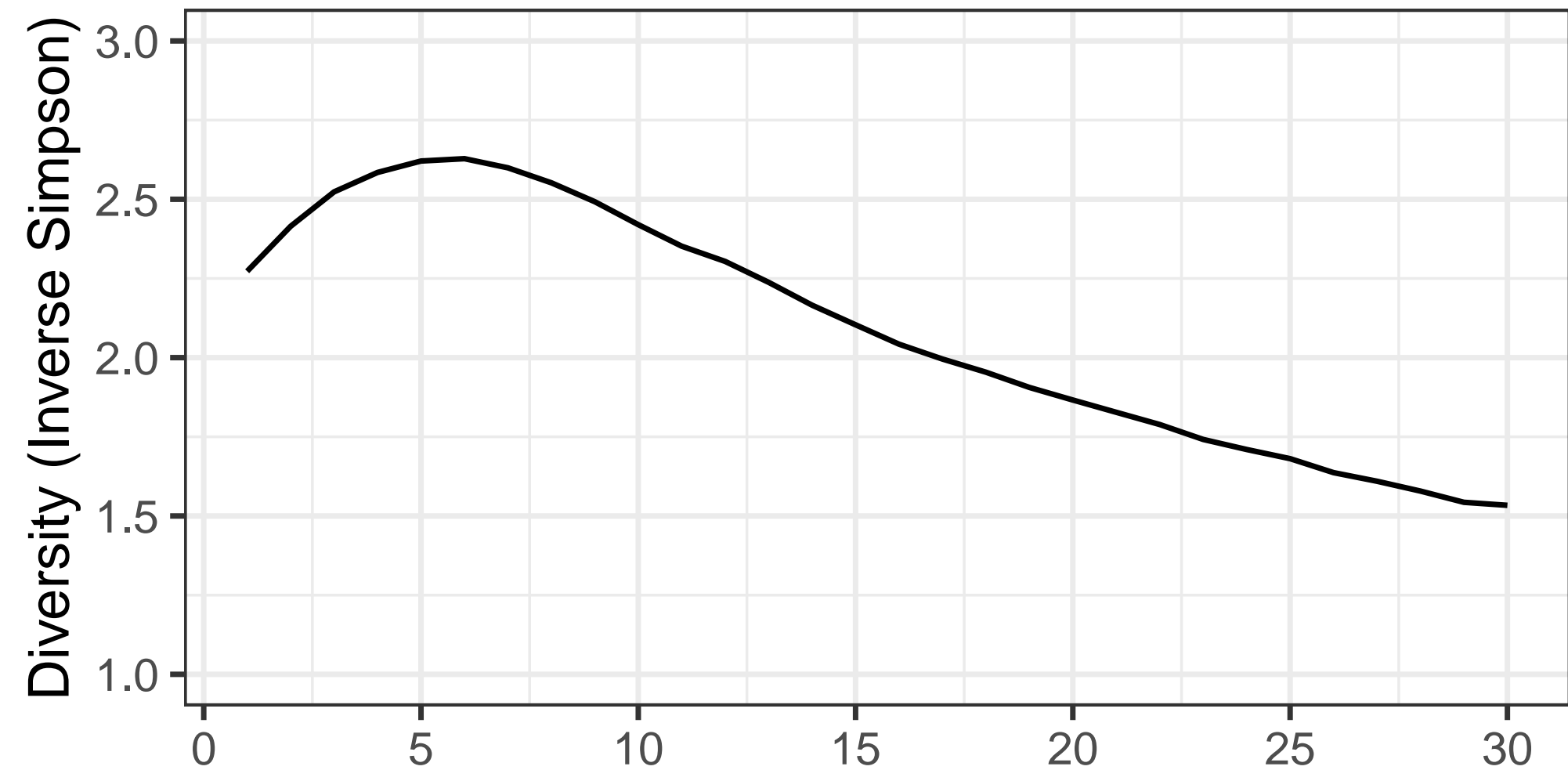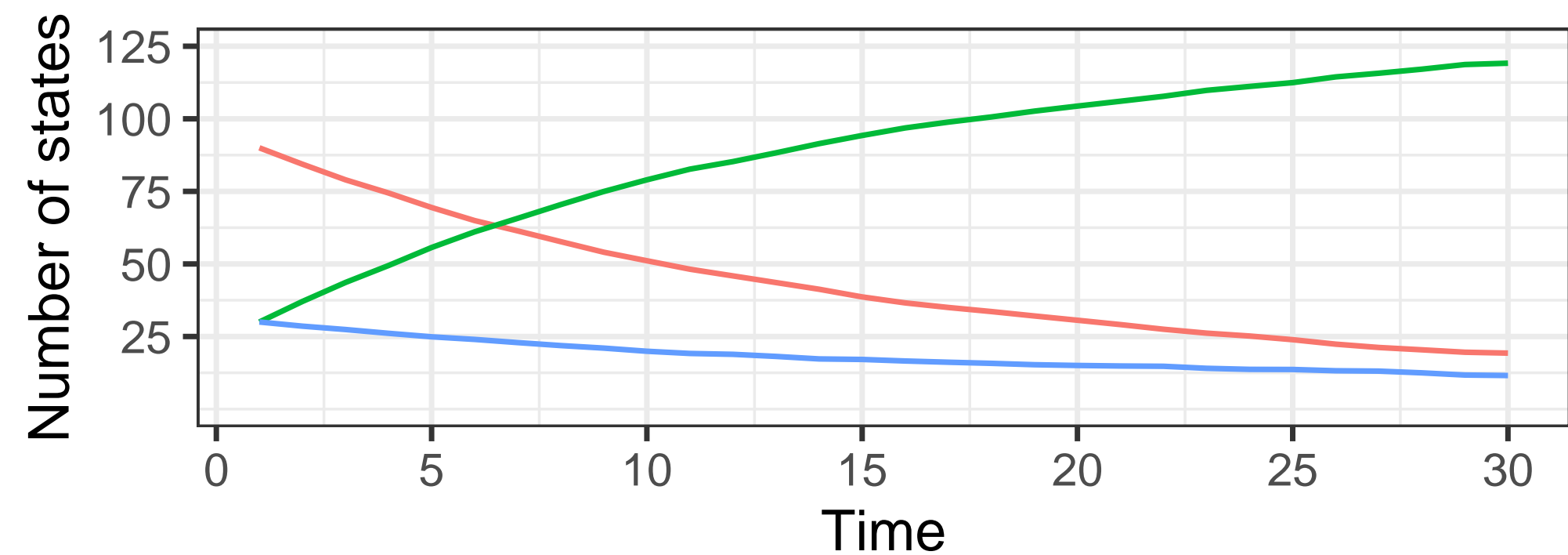

Policy

- Initially popular rule
- Promoted rule
- Third rule

Supplement: Supplementary file 2 — Supplementary file2 (ZIP 112225 kb) [file 11558_2024_9545_MOESM2_ESM.zip › The Defocalizing Effect - Replication/2 Analysis/2.1 R/Figures/Appendix Figure 4a.pdf]

Diversity (inverse Simpson)

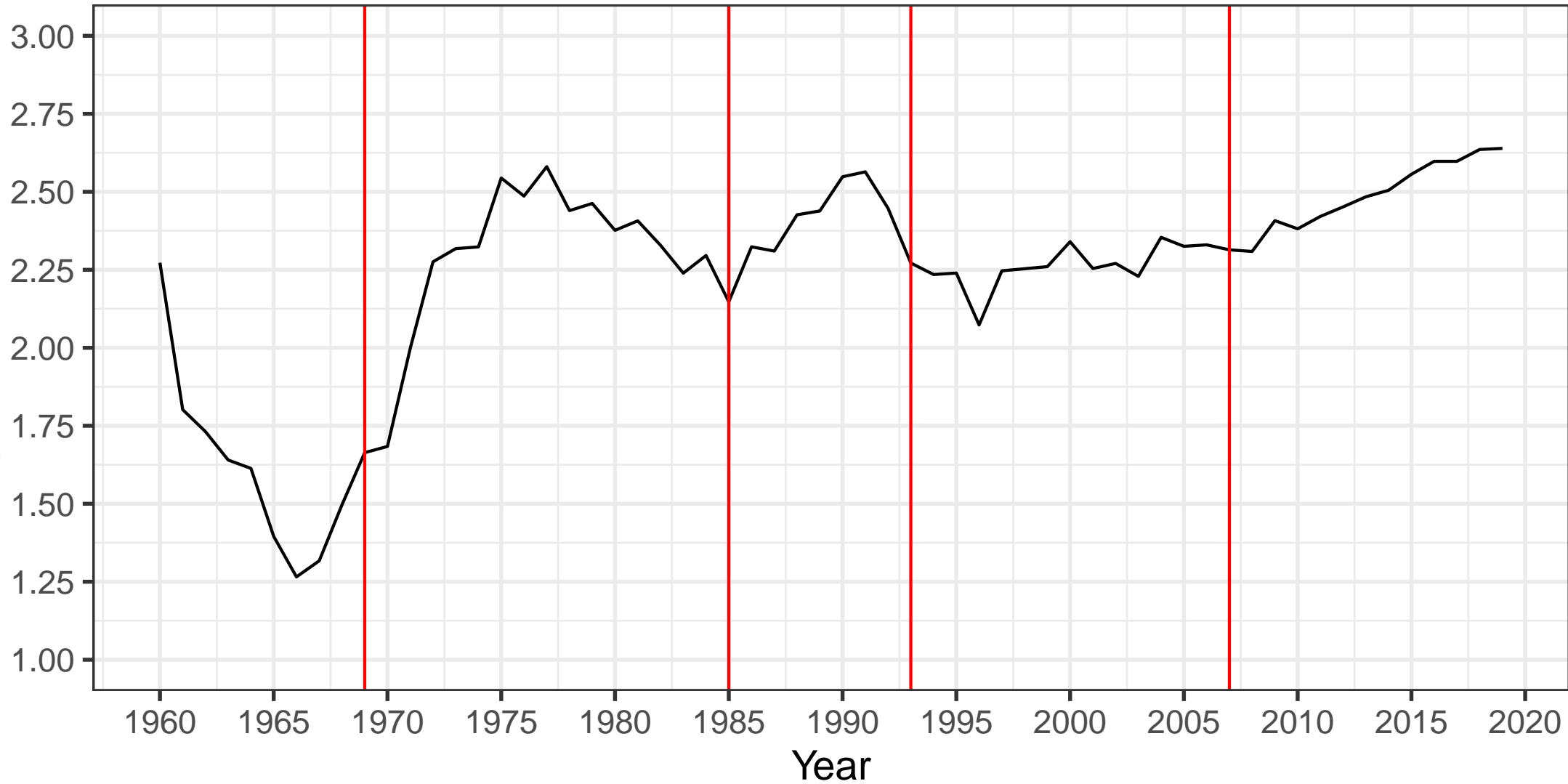

Supplement: Supplementary file 2 — Supplementary file2 (ZIP 112225 kb) [file 11558_2024_9545_MOESM2_ESM.zip › The Defocalizing Effect - Replication/2 Analysis/2.1 R/Figures/Main Figure 5.pdf]

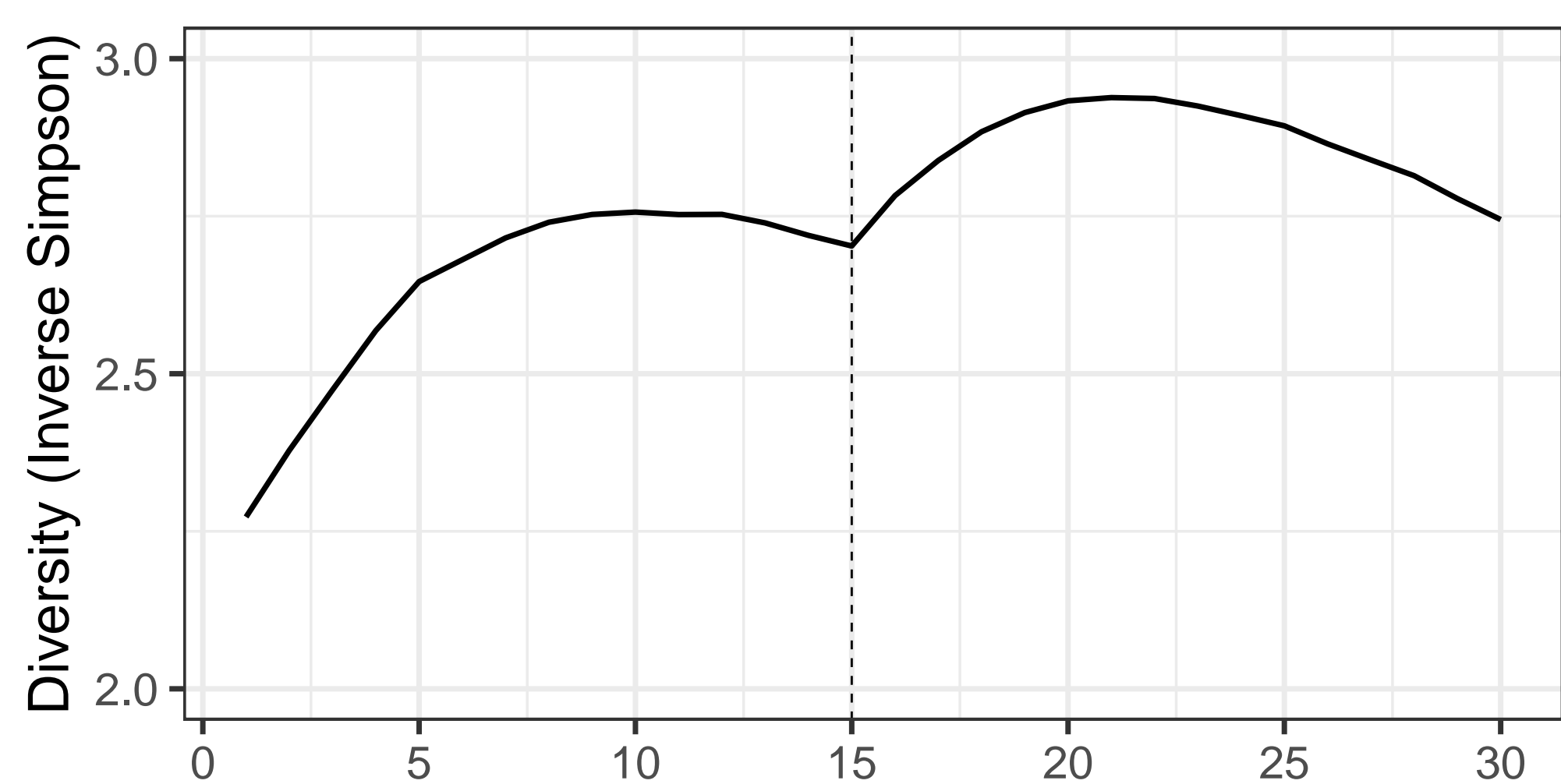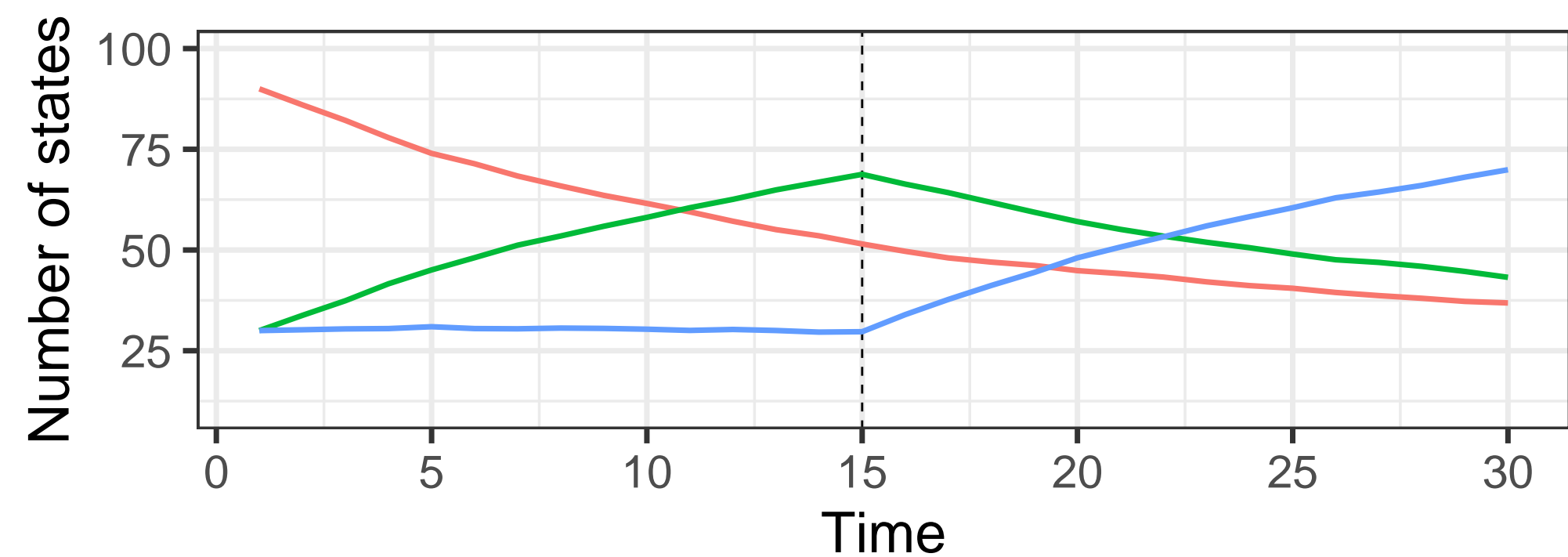

- Policy
- Initially popular rule
  - Promoted rule (until t=15)
  - Promoted rule (from t=15)

Supplement: Supplementary file 2 — Supplementary file2 (ZIP 112225 kb) [file 11558_2024_9545_MOESM2_ESM.zip › The Defocalizing Effect - Replication/2 Analysis/2.1 R/Figures/Main Figure 2b.pdf]

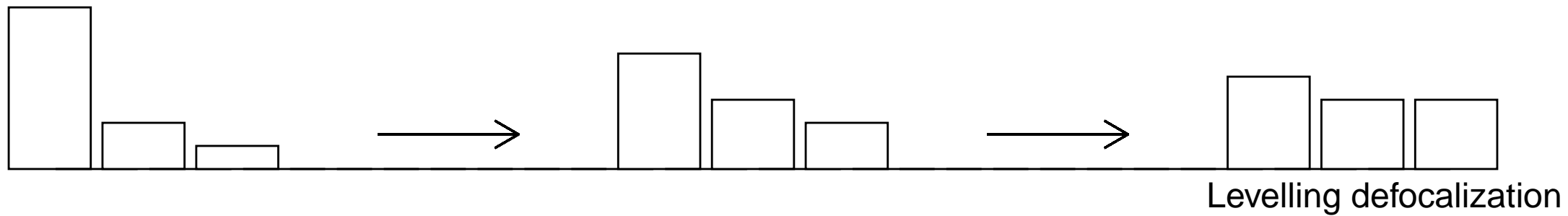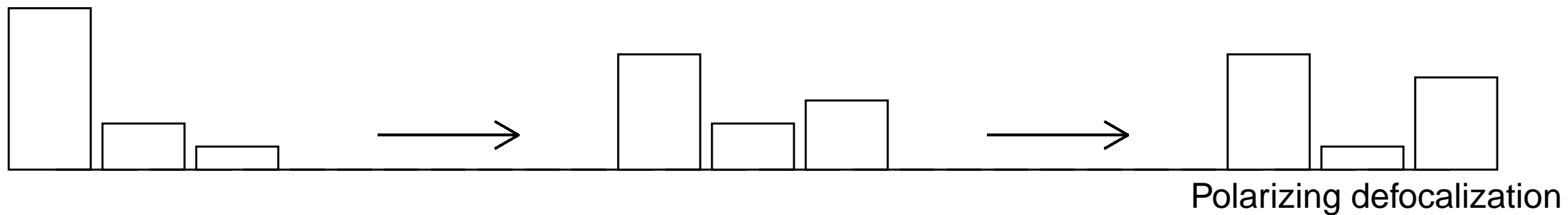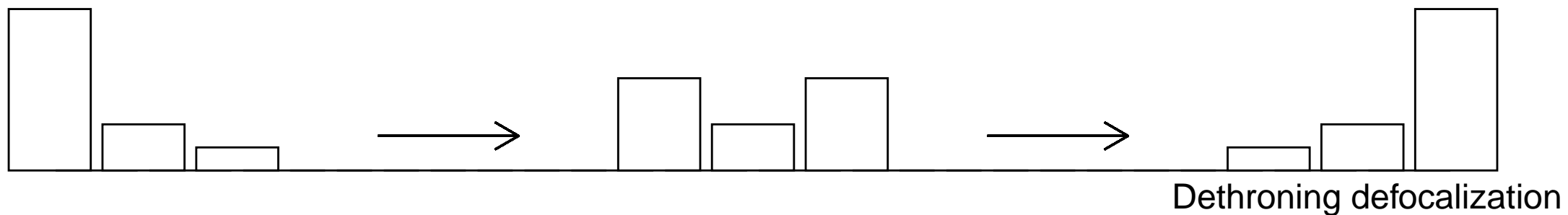

Supplement: Supplementary file 2 — Supplementary file2 (ZIP 112225 kb) [file 11558_2024_9545_MOESM2_ESM.zip › The Defocalizing Effect - Replication/2 Analysis/2.1 R/Figures/Main Figure 1.pdf]

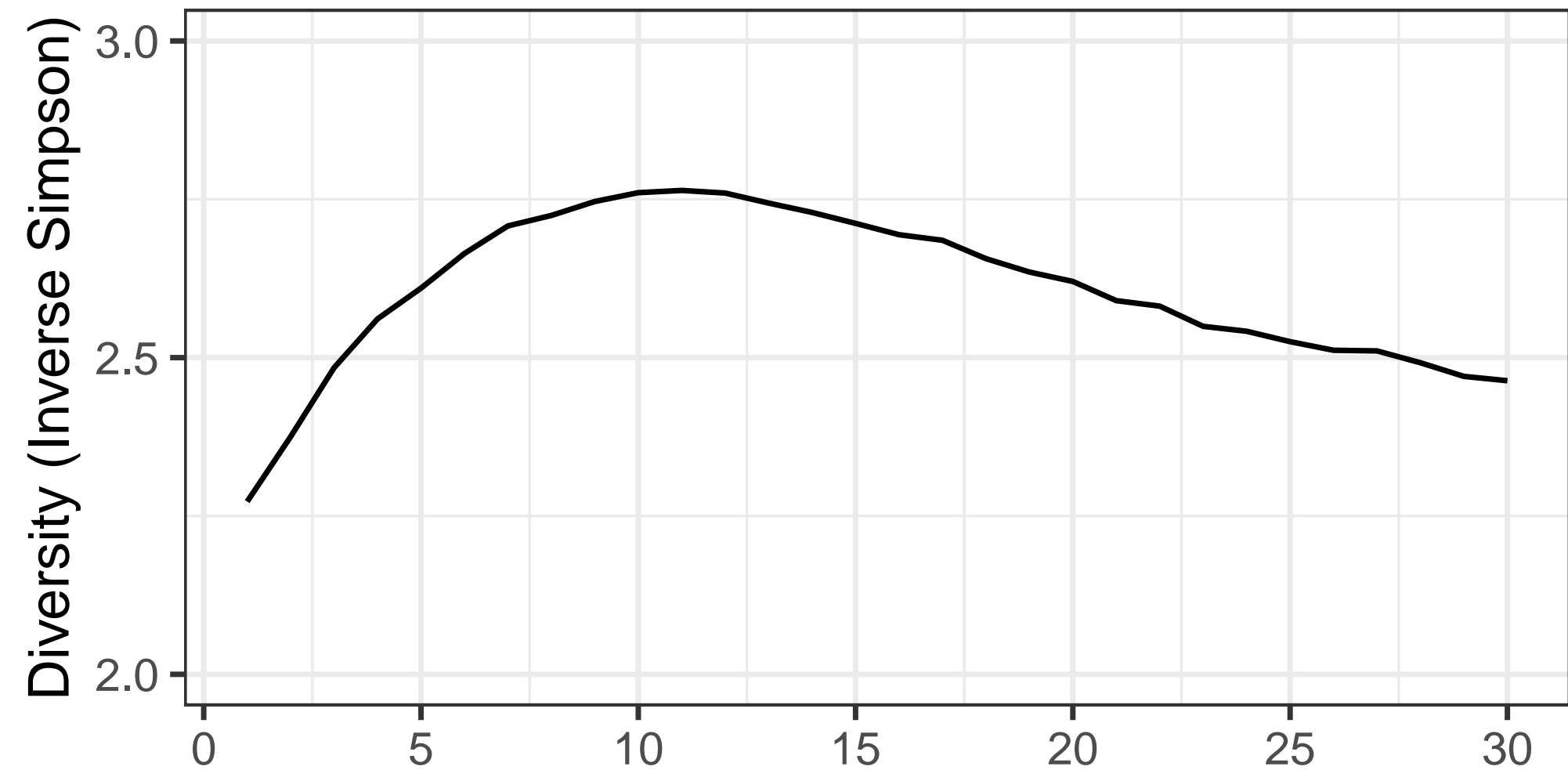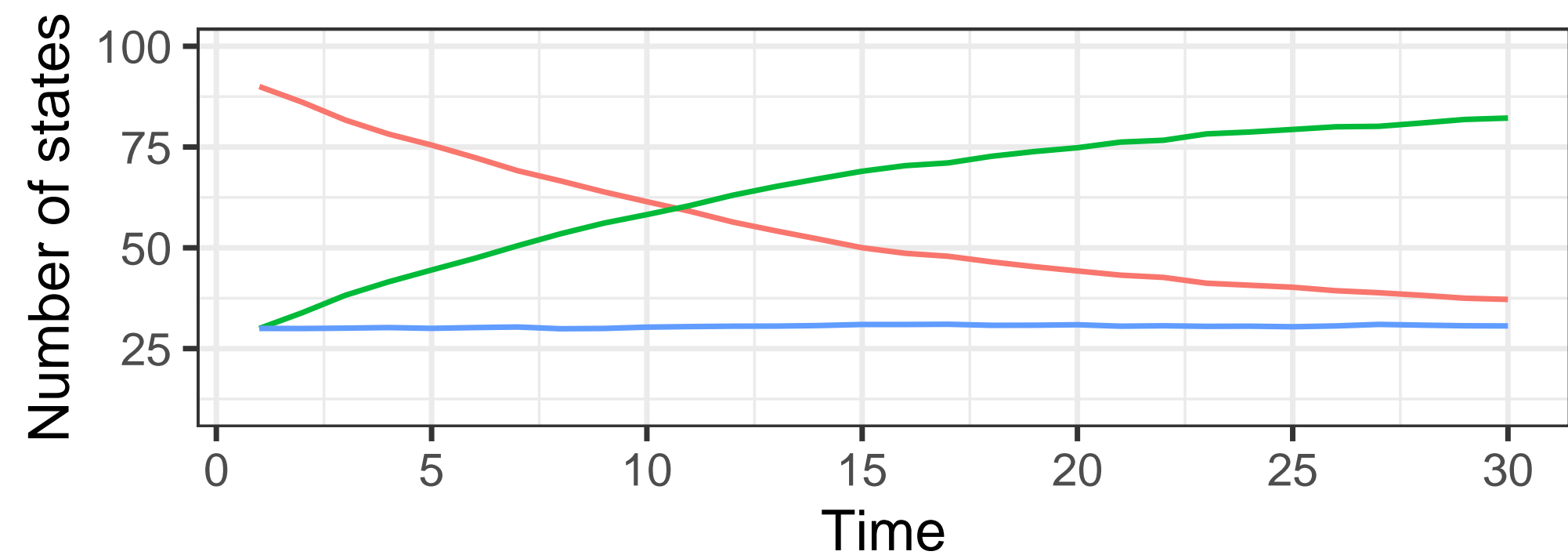

Policy

- Initially popular rule
- Promoted rule
- Third rule

Supplement: Supplementary file 2 — Supplementary file2 (ZIP 112225 kb) [file 11558_2024_9545_MOESM2_ESM.zip › The Defocalizing Effect - Replication/2 Analysis/2.1 R/Figures/Main Figure 2a.pdf]

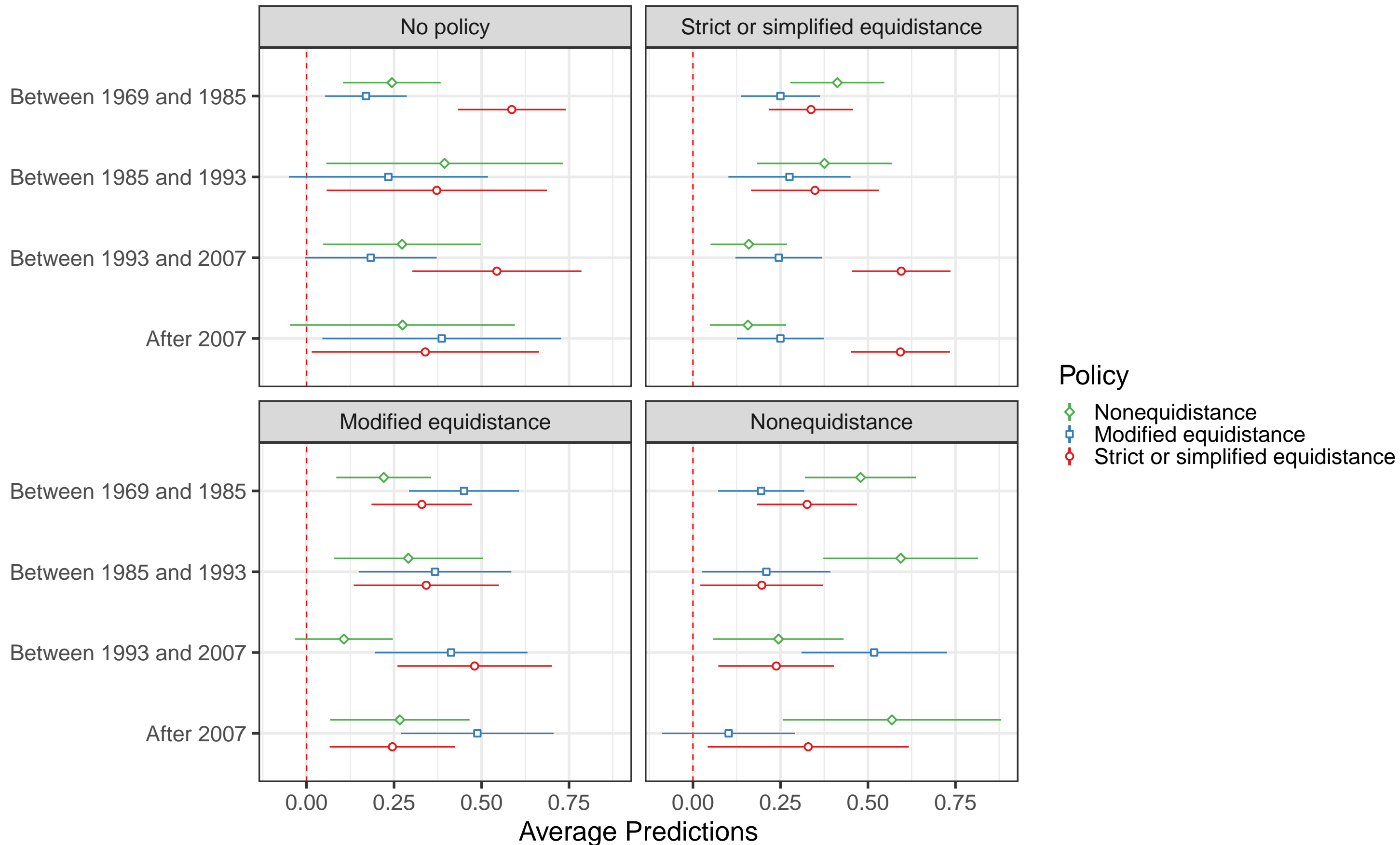

Supplement: Supplementary file 2 — Supplementary file2 (ZIP 112225 kb) [file 11558_2024_9545_MOESM2_ESM.zip › The Defocalizing Effect - Replication/2 Analysis/2.1 R/Figures/Main Figure 7.pdf]

Diversity (inverse Simpson)

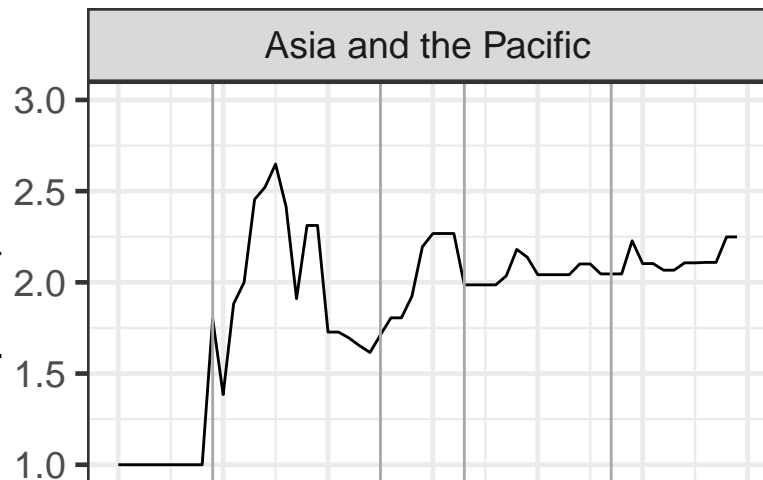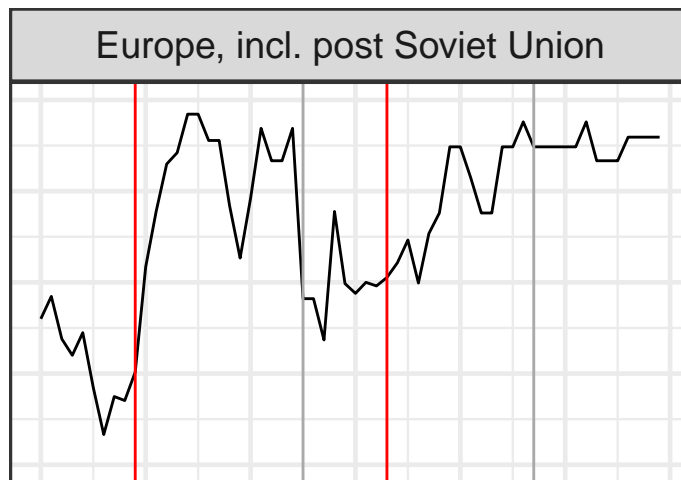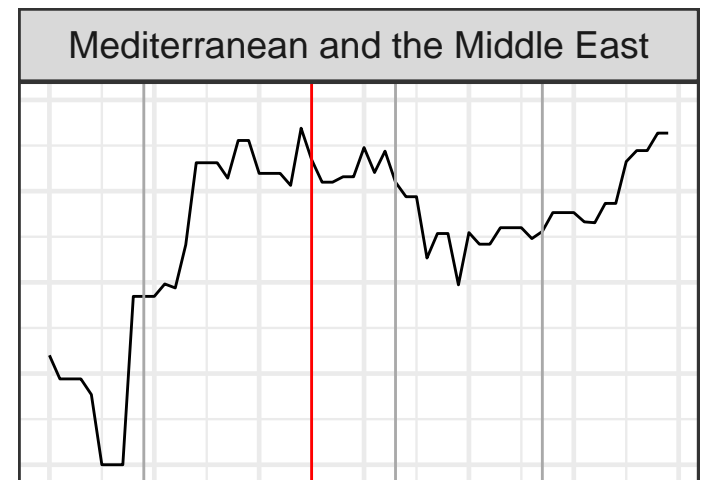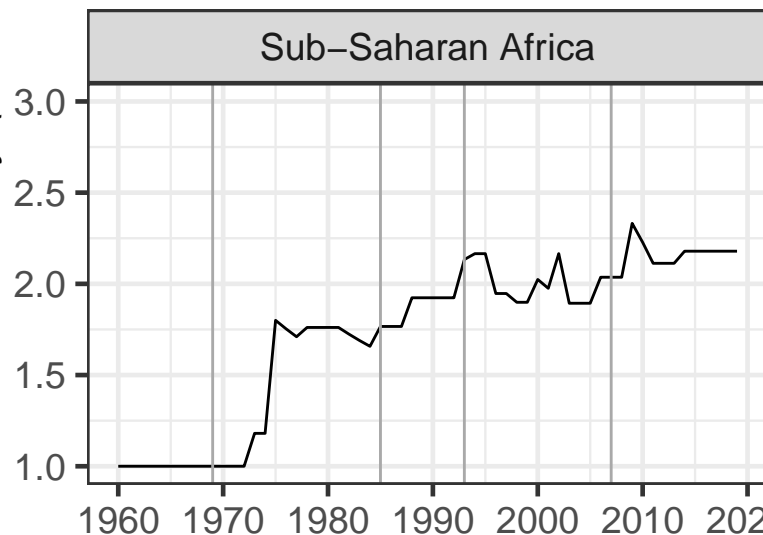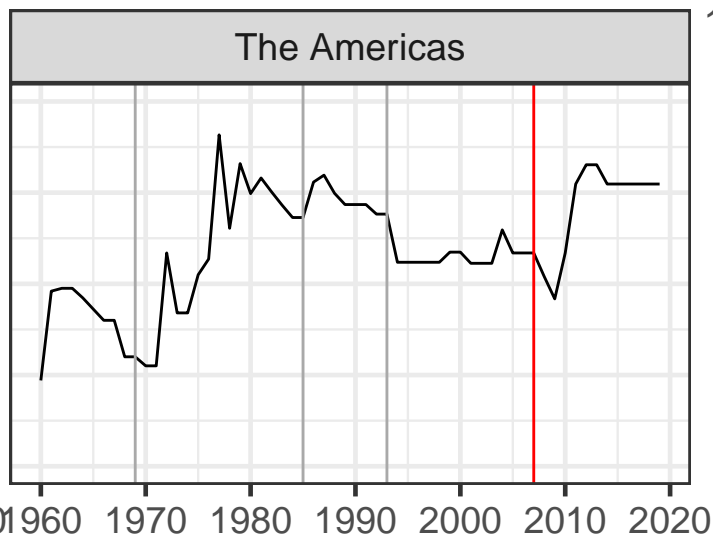

Year

Supplement: Supplementary file 2 — Supplementary file2 (ZIP 112225 kb) [file 11558_2024_9545_MOESM2_ESM.zip › The Defocalizing Effect - Replication/2 Analysis/2.1 R/Figures/Appendix Figure 3.pdf]

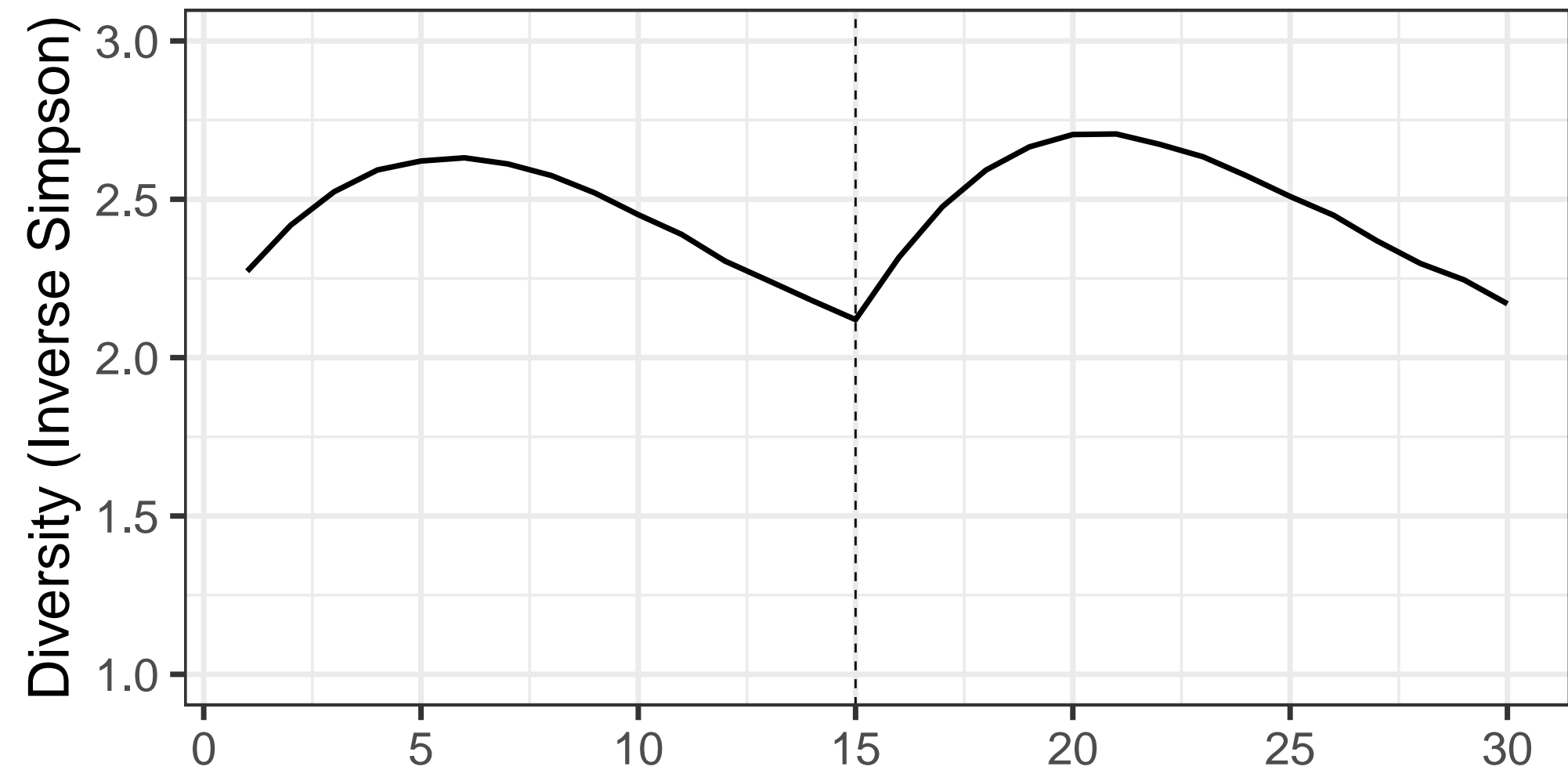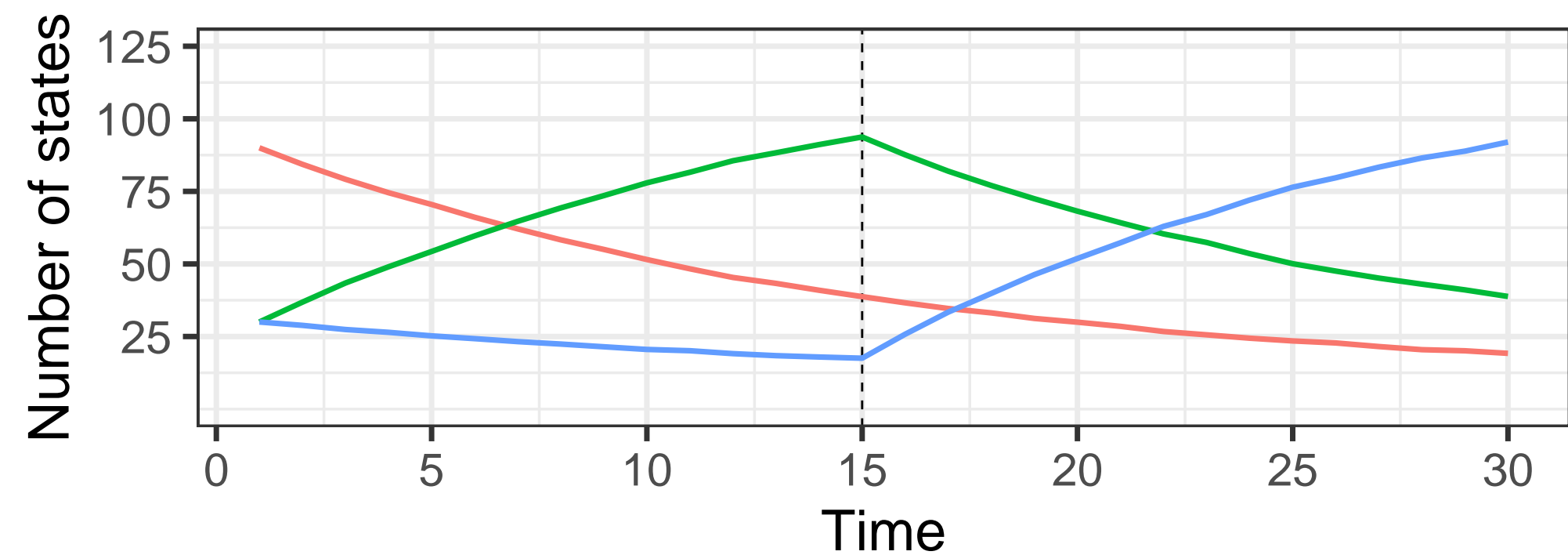

- Policy
- Initially popular rule
  - Promoted rule (until t=15)
  - Promoted rule (from t=15)

Supplement: Supplementary file 2 — Supplementary file2 (ZIP 112225 kb) [file 11558_2024_9545_MOESM2_ESM.zip › The Defocalizing Effect - Replication/2 Analysis/2.1 R/Figures/Appendix Figure 4b.pdf]

Number of states

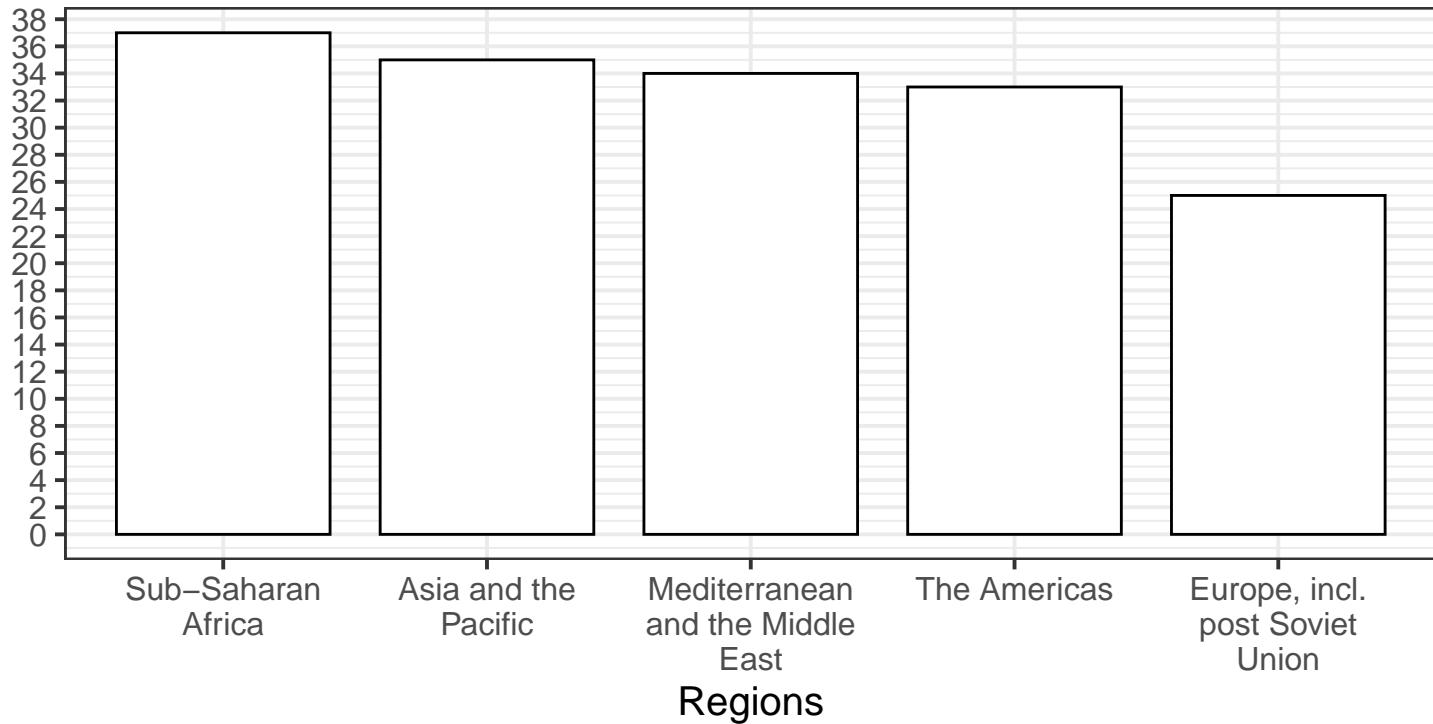

Supplement: Supplementary file 2 — Supplementary file2 (ZIP 112225 kb) [file 11558_2024_9545_MOESM2_ESM.zip › The Defocalizing Effect - Replication/2 Analysis/2.1 R/Figures/Appendix Figure 1.pdf]

# Predictive margins with 95% CIs

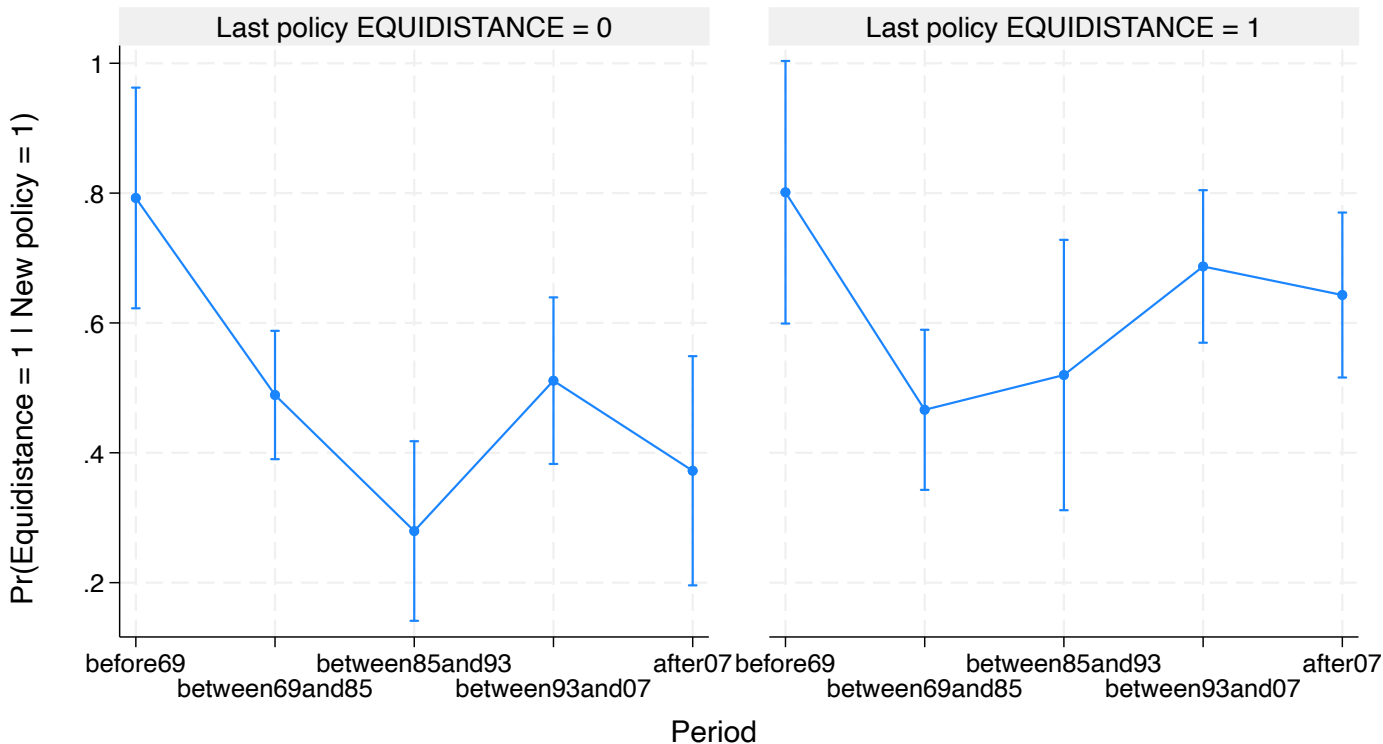

Supplement: Supplementary file 2 — Supplementary file2 (ZIP 112225 kb) [file 11558_2024_9545_MOESM2_ESM.zip › The Defocalizing Effect - Replication/2 Analysis/2.1 R/Figures/Appendix Figure 9.pdf]

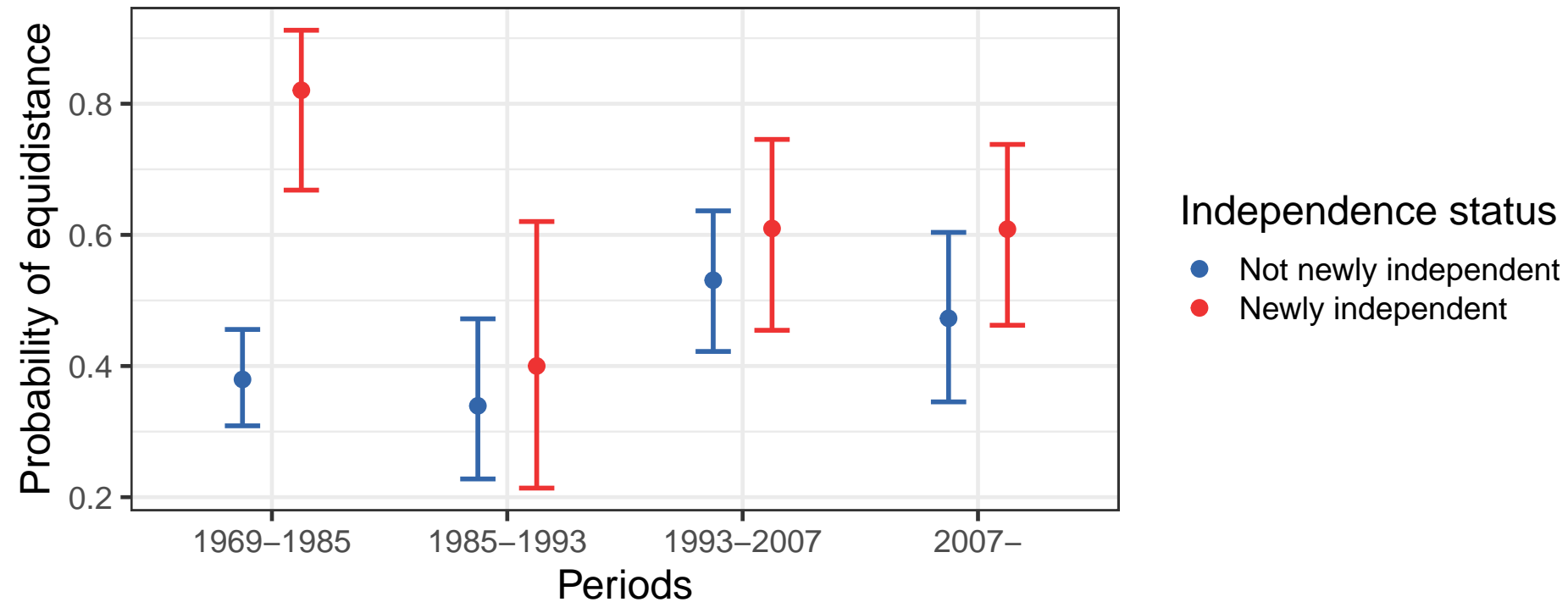

Supplement: Supplementary file 2 — Supplementary file2 (ZIP 112225 kb) [file 11558_2024_9545_MOESM2_ESM.zip › The Defocalizing Effect - Replication/2 Analysis/2.1 R/Figures/Appendix Figure 8.pdf]

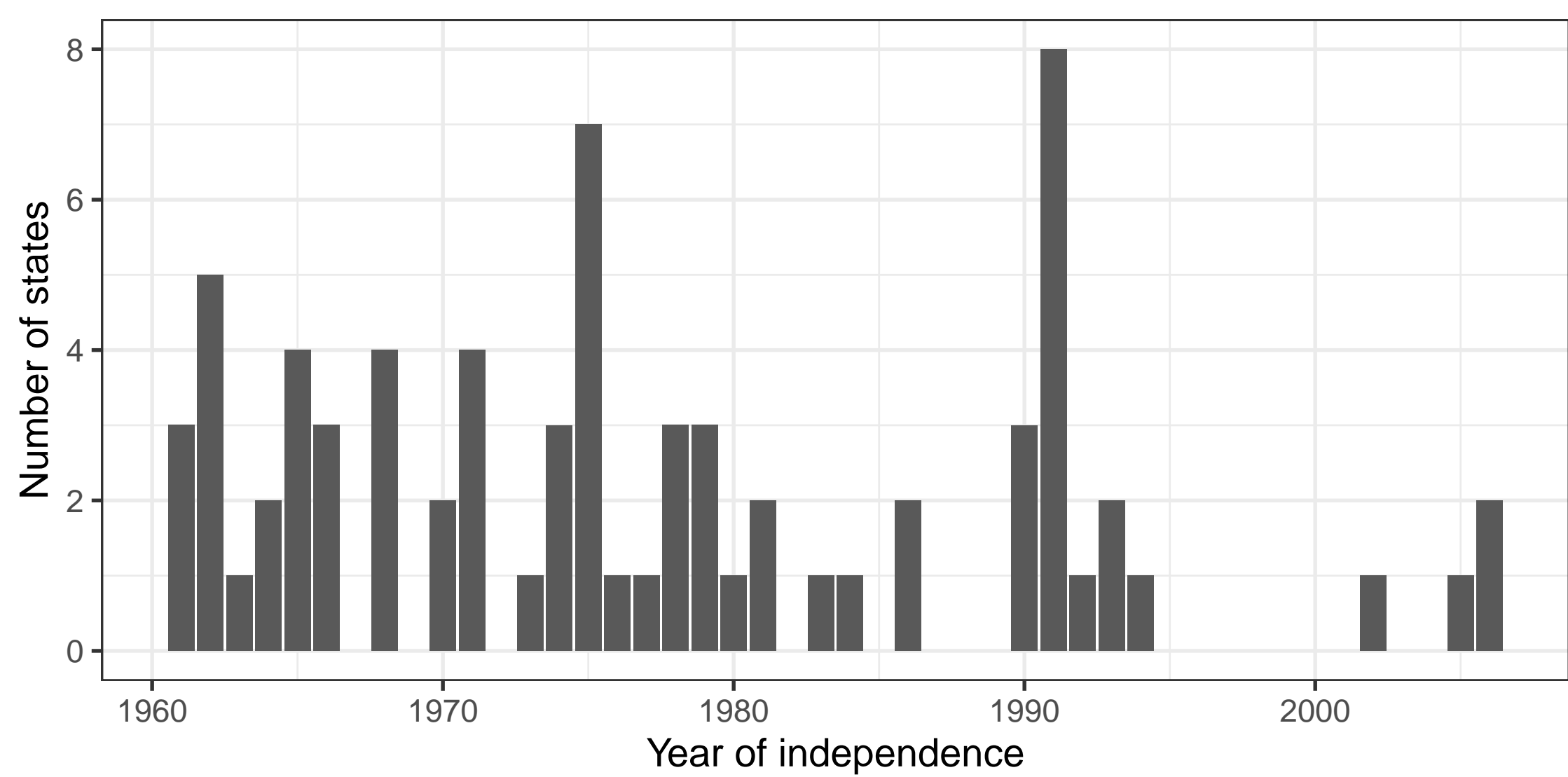

Supplement: Supplementary file 2 — Supplementary file2 (ZIP 112225 kb) [file 11558_2024_9545_MOESM2_ESM.zip › The Defocalizing Effect - Replication/2 Analysis/2.1 R/Figures/Appendix Figure 6.pdf]

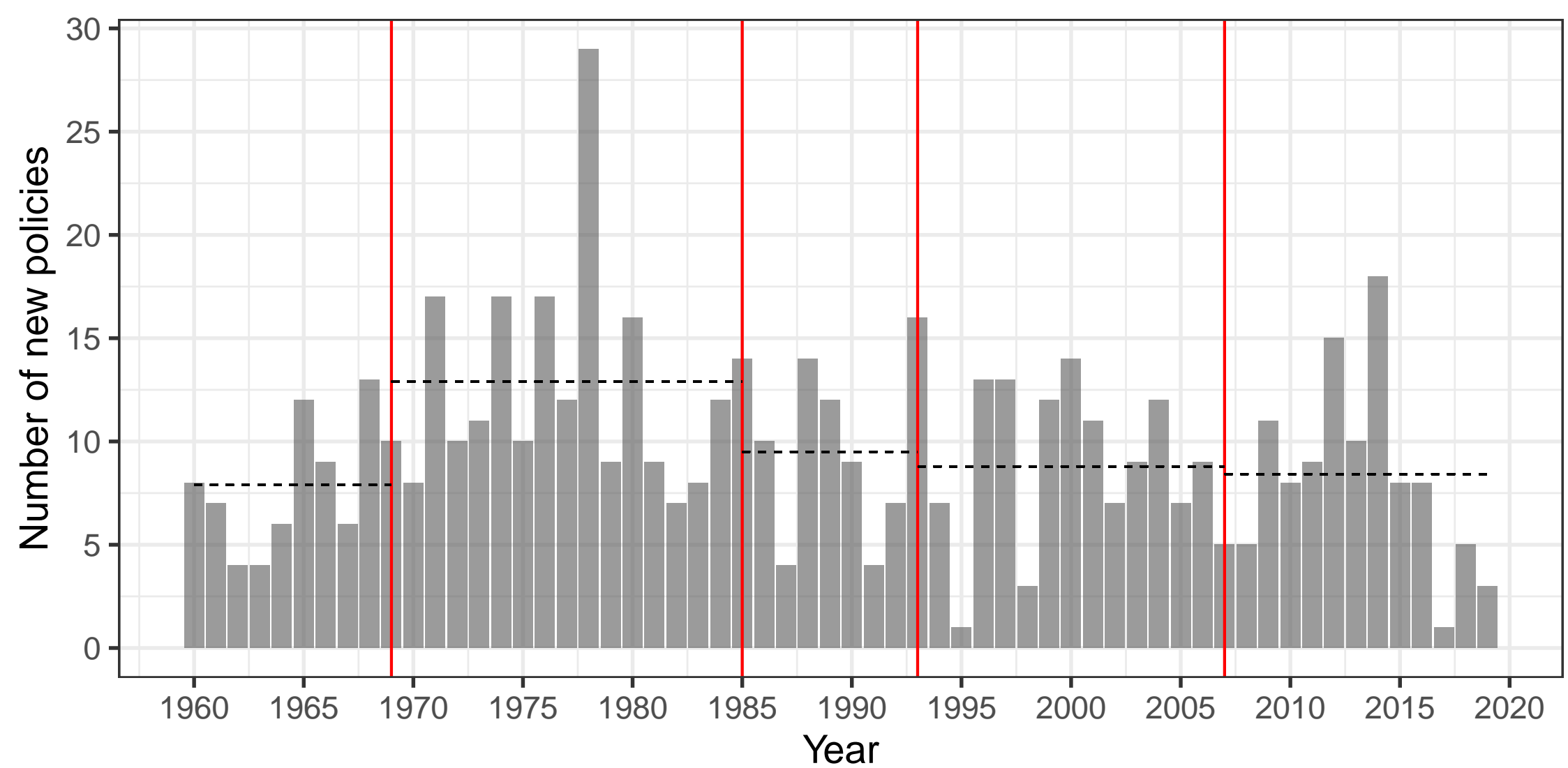

Supplement: Supplementary file 2 — Supplementary file2 (ZIP 112225 kb) [file 11558_2024_9545_MOESM2_ESM.zip › The Defocalizing Effect - Replication/2 Analysis/2.1 R/Figures/Main Figure 3.pdf]

Number of states

40  
30  
20  
10  
0

0 1 2 3 4 5 6 7 8 9 10 11 12 13 14 15 16 17 18 19 20 21 22 23 24 25

Number of new policies

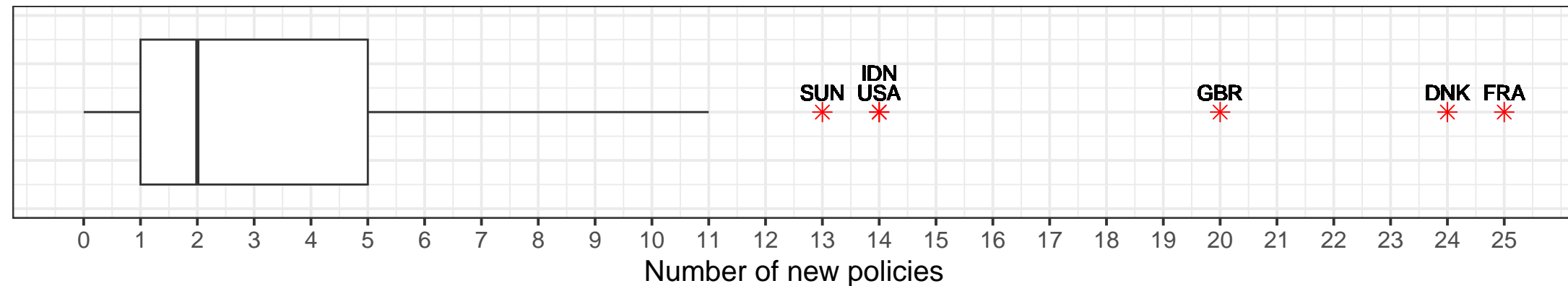

Supplement: Supplementary file 2 — Supplementary file2 (ZIP 112225 kb) [file 11558_2024_9545_MOESM2_ESM.zip › The Defocalizing Effect - Replication/2 Analysis/2.1 R/Figures/Appendix Figure 5.pdf]
